# Exploring the interplay between EBV and autophagy-related gene expression patterns in nasopharyngeal carcinoma

**DOI:** 10.3389/fonc.2025.1588921

**Published:** 2025-06-24

**Authors:** Abdullah E. Alanazi, Maaweya Awadalla, Amani Alowaini, Saad Alamri, Doaa Ali AlGhamdi, Alyaa S. Abdel Halim, Mohamed A. M. Ali, Bandar Alosaimi

**Affiliations:** ^1^ Saudi Food and Drug Authority, Riyadh, Saudi Arabia; ^2^ Research Center, King Fahad Medical City, Riyadh Second Health Cluster, Riyadh, Saudi Arabia; ^3^ Central Military Laboratory and Blood Bank, Prince Sultan Military Medical City, Riyadh, Saudi Arabia; ^4^ Pathology and Clinical Laboratory Medicine Administration, King Fahad Medical City, Riyadh Second Health Cluster, Riyadh, Saudi Arabia; ^5^ Department of Biochemistry, Faculty of Science, Ain Shams University, Cairo, Egypt; ^6^ Department of Biology, College of Science, Imam Mohammad Ibn Saud Islamic University (IMSIU), Riyadh, Saudi Arabia

**Keywords:** autophagy, Epstein-Barr virus, nasopharyngeal carcinoma, prognostic biomarker, clinical outcome

## Abstract

**Introduction/Aim:**

While components of the autophagy pathway have been linked to cancer prognosis, their relationship with clinical outcomes remains unclear. This study investigates the expression levels of autophagy-related genes (ATGs) at both mRNA and protein levels in nasopharyngeal carcinoma (NPC) and their association with Epstein-Barr virus (EBV) infection, clinicopathological characteristics, and clinical outcomes.

**Material and Methods:**

Thirty-five formalin-fixed, paraffin-embedded (FFPE) tissue specimens from NPC patients and five non-cancerous nasopharyngeal mucosa control samples were analyzed. Quantitative PCR (qPCR) was used to assess the mRNA levels of nine ATGs, while protein expression was evaluated by immunohistochemistry.

**Results:**

The results showed that ATG3, ATG4D, ATG4C, ATG4A, ATG2B, and ATG5 expression were significantly higher in EBV-positive NPC, suggesting a notable role of EBV in modulating these genes. Expression of ATG3 and ATG4C proteins was significantly more frequent in EBV-positive NPC patients compared to EBV-negative patients, with a strong correlation between expression of ATG3 or ATG4C and EBV positivity (P = 0.002 for both proteins). Elevated ATG4B and ATG4D expression was significantly associated with reduced distant metastasis in NPC patients (P = 0.019) and within the EBV-positive subgroup (P = 0.014). Reduced ATG4D mRNA levels were also correlated with higher metastasis rates and shorter distant metastasis-free survival (DMFS), highlighting a potential association of ATG4D with DMFS.

**Conclusions:**

Overall, these findings emphasize the importance of assessing autophagy-related gene expression as a valuable tool for predicting clinical outcomes in NPC and underscore the need for further research to validate these results and explore therapeutic implications.

## Graphical Abstract

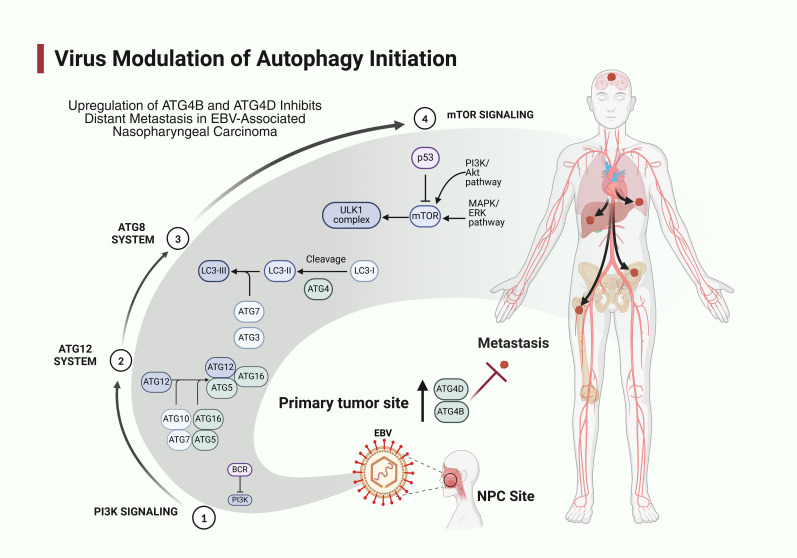

## Introduction

Nasopharyngeal carcinoma (NPC) is an aggressive epithelial carcinoma originating from the nasopharyngeal mucosal lining, presenting a considerable public health challenge. It is a distinct type of head and neck cancer that exhibits a strong association with Epstein-Barr virus (EBV) infection. Epidemiological studies indicate that approximately 80% to 90% of NPC cases in endemic regions, such as Southeast Asia, are associated with EBV, highlighting the virus’s pivotal role in the pathogenesis of this malignancy. In non-endemic regions, while the prevalence of NPC is lower, a significant proportion of cases (about 20% to 30%) still show evidence of EBV involvement. This strong correlation underscores the importance of understanding the molecular mechanisms through which EBV influences NPC development, particularly in relation to autophagy-related gene expression (1). In 2022, the International Agency for Research on Cancer (IARC) reported around 120,416 new cases of NPC, representing 0.6% of all cancer diagnoses that year, and this malignancy accounted for 73,476 cancer-related fatalities globally in 2022 (2).

Nasopharyngeal carcinoma (NPC) exhibits a unique geographical distribution. The continued prevalence of this malignancy in specific geographic areas indicates that genetic and/or consistent environmental risk factors significantly contribute to its emergence. While genetic predisposition accounts for the clustering occurrence of this disease in endemic communities, possibly influenced by environmental variables, EBV infection is arguably the most prevalent causative agent of NPC. EBV, a gamma-herpesvirus, has a double-stranded DNA genome of approximately 172 kb that encodes for more than 85 genes. EBV infection is ubiquitous, with over 90% of the global adult population carrying the virus in a latent form. While asymptomatic in most individuals, EBV is etiologically linked to several malignancies, including Burkitt’s lymphoma, Hodgkin’s lymphoma, gastric carcinoma, and, most notably, NPC, where it shows the strongest association. EBV’s oncogenic potential in NPC stems from its unique latency type II expression pattern, characterized by expression of Epstein-Barr nuclear antigen 1 (EBNA1), latent membrane proteins (LMP1, LMP2A, and LMP2B), EBV-encoded small RNAs (EBERs), and BamHI-A rightward transcripts (BARTs). LMP1 is the principal oncogene, activating multiple signaling pathways, including NF-κB, JNK, and PI3K/Akt, which promote cell proliferation, transformation, and metastasis (3, 4). EBV, the first identified human oncovirus, facilitates the neoplastic transformation of nasopharyngeal epithelial cells by diverse molecular processes, mostly involving the activation of oncogenes and the inactivation of tumor-suppressor genes. EBV infection can induce epigenetic alterations in the infected cells that may promote tumor proliferation through DNA methylation, histone modifications, and chromatin remodeling (5).

Autophagy, an evolutionarily conserved cellular mechanism, is a meticulously regulated system for the breakdown and recycling of intracellular components, vital for sustaining cellular homeostasis and serving as a fundamental pro-survival route. The process involves the formation of double-membrane vesicles called autophagosomes that engulf cytoplasmic constituents and subsequently fuse with lysosomes for degradation. Autophagy can be stimulated by a range of conditions, such as metabolic stress, nutritional scarcity, lack of growth factors and hypoxia, facilitating adaptability to alterations in the microenvironment. It participates in diverse cellular functions such as apoptosis, survival and differentiation, contingent upon the cell types and the degree of process activation (6). The core molecular machinery of autophagy consists of several autophagy-related genes (ATGs) initially identified in yeast but highly conserved in mammals. These include the ULK1 complex (ULK1, ATG13, FIP200), which initiates autophagy; the Beclin-1/class III PI3K complex (Beclin-1, VPS34, ATG14) responsible for nucleation; and two ubiquitin-like conjugation systems (ATG12-ATG5-ATG16L1 and LC3-PE) that mediate autophagosome elongation and completion. ATG3, for instance, is an essential component of the autophagy machinery that facilitates the conjugation of the ubiquitin-like protein LC3 (microtubule-associated protein 1A/1B-light chain 3) to phosphatidyl-ethanolamine, a critical step in autophagosome formation. Similarly, ATG4C is a cysteine protease that plays a vital role in the autophagy process by regulating the lipidation of LC3. It is involved in the cleavage of the precursor form of LC3, enabling its conjugation to phosphatidylcholine. This function is crucial for the maturation of autophagosomes and the subsequent degradation of cellular debris. In NPC, elevated levels of ATG4C may contribute to the dysregulation of autophagy, supporting cancer cell proliferation and survival. ATG4D, another member of the ATG4 family, also participates in the processing of LC3 and has been implicated in the regulation of autophagy dynamics. Its role extends to modulating the fate of autophagosomes, influencing their fusion with lysosomes and the subsequent degradation of their contents. In NPC, altered expression of ATG4D has been associated with changes in metastasis and treatment responses, suggesting that it may act as a potential prognostic biomarker (3, 4, 7). The influence of autophagy on cellular physiology is variable and contingent on the specific conditions of the afflicted cell (7). Autophagy dysregulation is also linked to several diseases (8).

Autophagy is a crucial component of both the innate and adaptive immune systems. It eradicates infections and prepares their remnants for presentation to the immune system. It specifically identifies intracellular microorganisms and directs these pathogens to the autophagy process for destruction. This process, termed xenophagy, involves selective recognition of pathogens through adaptors like p62/SQSTM1, NDP52, and optineurin that bind to ubiquitinated pathogen surfaces and target them for autophagic degradation. Autophagy is crucial in regulating viral infections, and viruses are known to exploit the host’s autophagy mechanisms. Viruses have developed mechanisms to disrupt the host’s autophagic processes to evade elimination and enhance their replication and dissemination (9).

A complex relationship with autophagy has been documented in the specific context of EBV infection. EBV has evolved sophisticated strategies to modulate autophagy to promote viral persistence and oncogenesis. During lytic replication, EBV induces autophagy through several viral proteins, including Rta, which upregulates ATG5 and LC3 expression. Conversely, during latent infection, EBV impairs autophagic flux to prevent viral degradation and enhance cell survival. The viral LMP1 protein activates mTOR signaling, a major negative regulator of autophagy, while simultaneously inducing autophagy through NF-κB activation, creating a delicate balance that benefits viral persistence. Another viral protein, EBNA1, has been shown to suppress autophagy by directly interacting with NAF1, a component of the Beclin-1 complex. Several ATGs have been specifically implicated in EBV-associated NPC pathogenesis. Studies have demonstrated altered expression of key autophagy components, including LC3B, p62/SQSTM1, LAMP1, and ULK1 in NPC tissues compared to adjacent normal nasopharyngeal epithelium. Moreover, EBV-positive NPC cell lines often display distinctive autophagy signatures compared to EBV-negative counterparts, with differential ATG5, ATG7, and BECN1 expression, suggesting EBV-specific modulation of the autophagy machinery. Increased expression of ATG3 has been linked to enhanced autophagic activity, promoting tumor cell survival under stress conditions, which is particularly relevant in the tumor microenvironment of NPC. In the setting of EBV, viral proteins engage with autophagic proteins, thereby hijacking the autophagic process to facilitate viral replication. It has been proposed that the autophagic machinery is altered to restrict the breakdown of viral components, enabling autophagic vesicles to assist in virus packing and release (10).

The function of autophagy in relation to cancer is contentious and seems to differ significantly between pre-malignant and post-malignant conditions. Autophagy exhibits multiple roles in carcinogenesis, functioning as both a tumor promoter and a tumor suppressor. The function of autophagy in carcinogenesis is intricate and may have contradictory effects on tumor viability based on certain pathophysiological conditions. The activation of autophagy may serve as a tumor suppressor by destroying faulty organelles and other cellular constituents. Conversely, cancer cells may employ autophagy to produce nutrients and energy during nutritional deprivation, hypoxia, or other therapeutic stress responses, typically safeguarding against cell death and promoting adaptive survival (11). Growing evidence suggests that autophagy plays a crucial role in regulating cancer formation and progression and influencing the responses of tumor cells to anticancer therapy across various malignancies. Clinical investigations into diverse cancer types indicate that both low and high expression levels of autophagy-related proteins are linked to a poor prognosis. Consequently, the association between autophagy and patient clinical outcomes remains contentious (12). Autophagy dysfunction has been associated with a range of illnesses (13). Numerous studies have demonstrated that autophagy is crucial in the genesis and progression of cancer. The breakdown of oncogenic protein substrates, toxic proteins and dysfunctional organelles is proposed to facilitate the tumor-suppressive effects of autophagy. Conversely, autophagy-mediated intracellular recycling of substrates essential for mitochondrial function has a tumor-promoting effect on cancer cells. While the role of autophagy in cancer, whether promoting or inhibiting, remains contentious, its significance is indisputable (14). While it has been established that components of the autophagy system correlate with patient prognosis in several human malignancies, the relationship between autophagy and clinical outcomes of patients is disputed (15). Recent studies have concentrated on biomarkers that may be utilized in targeted therapies and prognostic predictions. While numerous molecular markers have been recognized to forecast prognosis in NPC, it would significantly benefit NPC patients if new effective biomarkers could be discovered that assist in prognostic prediction, offering promising therapeutic targets for treatment (16). The interplay between EBV infection and autophagy regulation in NPC presents a compelling area for investigation, with potential therapeutic implications. Recent research has explored targeting autophagy as a treatment strategy for EBV-associated malignancies, including NPC. Autophagy inhibitors such as chloroquine and hydroxychloroquine have shown promising results in preclinical models of EBV-positive NPC, enhancing the efficacy of conventional treatments and overcoming therapeutic resistance. Conversely, autophagy inducers like rapamycin and its analogs have demonstrated efficacy in certain contexts by promoting autophagic cell death in EBV-infected cells. Therefore, understanding the precise relationship between EBV and ATG expression patterns is crucial for developing targeted therapeutic approaches for NPC patients (16). Given their emerging role as promising biological targets for cancer therapy, autophagy-related genes and proteins have attracted considerable attention in recent years.

This study aimed to investigate the expression levels of autophagy-related genes (ATGs) at both the mRNA and protein levels in nasopharyngeal carcinoma (NPC). Their association with EBV status, clinicopathological characteristics, and clinical outcomes were also explored.

## Patients and methods

### Study cohort and patients′ tissue specimens

This retrospective analysis comprised 35 patients with a pathologically confirmed diagnosis of primary NPC (30 EBV-positive and 5 EBV-negative) enrolled between January 2021 and December 2023. To verify EBV-associated status, all NPC cases underwent testing for latent EBV infection in formalin-fixed, paraffin-embedded (FFPE) tissue sections by detecting Epstein-Barr virus small non-coding non-polyadenylated nuclear RNA (EBER) through *in situ* hybridization (ISH) assay using a fluorescein-labeled EBER oligonucleotide probe, in conjunction with the Super Sensitive one-Step Polymer-HRP ISH Detection System (BioGenex Laboratories, Fremont, CA, USA), following the manufacturer’s guidelines. The selection of cases was based on the availability of biopsy specimens and follow-up data, excluding any history of radiotherapy, chemotherapy or oncological surgery. The investigation included whole sections of FFPE tissue obtained from 35 NPC patients using endoscopic biopsy at King Fahad Medical City in Riyadh, Saudi Arabia. Five tissue samples of nonmalignant nasopharyngeal mucosa were used as controls. Clinicopathological data of patients were retrospectively obtained from electronic medical records.

### Quantitative assessment of autophagy-related genes

The expression levels of nine autophagy-related genes ATG1/ULK1, ATG2A, ATG2B, ATG3, ATG4A, ATG4B, ATG4C, ATG4D, and ATG5 were quantified in formalin-fixed, paraffin-embedded (FFPE) tissue specimens using quantitative polymerase chain reaction (qPCR). These specific autophagy-related genes were selected based on several critical considerations. First, they represent key components across the sequential stages of the autophagy process: ATG1/ULK1 functions in the initiation phase as a serine/threonine kinase that forms the ULK complex; ATG2A and ATG2B are essential for autophagosome formation and lipid transfer between the endoplasmic reticulum and phagophore; ATG3 serves as an E2-like enzyme crucial for LC3 lipidation during autophagosome elongation; the ATG4 family (ATG4A, ATG4B, ATG4C, and ATG4D) comprises cysteine proteases responsible for processing pro-LC3 into its active form and recycling LC3 from autophagosome membranes; and ATG5 forms part of the ATG5-ATG12-ATG16L1 complex necessary for autophagosome membrane elongation. Second, previous studies have specifically implicated these genes in viral infection responses and EBV-mediated pathogenesis. Third, these genes have demonstrated clinical relevance in nasopharyngeal and other EBV-associated malignancies (17). Total RNA was extracted from the FFPE tissues using the AllPrep DNA/RNA FFPE Kit (Qiagen, Valencia, CA, USA) according to the manufacturer’s instructions. RNA samples were then reverse transcribed into complementary DNA (cDNA) using the QuantiTect Reverse Transcription Kit (Qiagen, Valencia, CA, USA), following the manufacturer’s guidelines. Gene expression levels were quantitatively evaluated using the QuantiTect SYBR Green PCR Kit and QuantiTect Primer Assays (Qiagen, Valencia, CA, USA). Relative gene expression was calculated using the comparative Ct method (2−ΔΔCt), with glyceraldehyde-3-phosphate dehydrogenase (GAPDH) serving as the endogenous control gene.

### Examination of autophagy-related proteins

The FFPE tissue specimens were analyzed for the expression of nine autophagy-related proteins: ATG1/ULK1, ATG2A, ATG2B, ATG3, ATG4A, ATG4B, ATG4C, ATG4D, and ATG5, using immunohistochemistry (IHC). The protein-level analysis of these specific ATGs was conducted to complement and validate the gene expression findings, providing a comprehensive assessment of autophagy dysregulation in NPC at both transcriptional and translational levels. Four-micrometer-thick, non-stained sections were prepared from whole FFPE tissue specimens, and IHC was performed, following standard procedures. The slides were dried in an oven at 60°C for one hour, deparaffinized with xylene, and then dehydrated through a series of graded ethanol solutions. IHC was conducted using the BOND-III Fully Automated IHC and ISH Staining System, along with the BOND Polymer Refine Detection kit, which includes a peroxide block, post-primary reagent, polymer reagent, DAB chromogen, and hematoxylin counterstain (Leica Biosystems, Nussloch, Germany), following the manufacturer’s instructions. Slides were mounted with DPX Mountant for histology (Sigma-Aldrich, Merck KGaA, MA, USA) and analyzed using a brightfield microscope (Olympus BX50; Olympus, Center Valley, PA, USA). Two independent pathologists, blinded to the patients’ clinicopathological data, evaluated the immunoreactivity of the autophagy-related proteins based on staining intensity: 0 (no staining, negative); 1 (light brown staining, weak positive); 2 (medium brown staining, moderate positive); and 3 (dark brown staining, strong positive). All primary antibodies were sourced from MyBioSource (San Diego, CA, USA). The following primary antibodies were used: Rabbit GAPDH Polyclonal Antibody (Catalog #: MBS9610383, 1:200 dilution); Rabbit anti-Human ULK1 Polyclonal Antibody (Catalog #: MBS8574584, 1:50 dilution); Rabbit anti-Human ATG2A Polyclonal Antibody (Catalog #: MBS8306077, 1:50 dilution); Rabbit ATG2B Polyclonal Antibody (Catalog #: MBS9128839, 1:50 dilution); Rabbit ATG3 Polyclonal Antibody (Catalog #: MBS2528893, 1:50 dilution); Rabbit ATG4A Polyclonal Antibody (Catalog #: MBS9240520, 1:50 dilution); Rabbit ATG4B Polyclonal Antibody (Catalog #: MBS9239798, 1:50 dilution); Rabbit ATG4C Polyclonal Antibody (Catalog #: MBS9239799, 1:50 dilution); Rabbit ATG4D Polyclonal Antibody (Catalog #: MBS9240444, 1:50 dilution); and Rabbit ATG5 Polyclonal Antibody (Catalog #: MBS125660, 1:50 dilution).

### Statistical analysis

Categorical variables were presented as the number of cases (%) and analyzed using Pearson’s chi-square (χ^2^) test or Fisher’s exact test, as applicable. Continuous variables were presented as mean ± standard deviation (SD) when normally distributed and compared using the independent Student’s t-test or one-way analysis of variance (ANOVA), where applicable. Conversely, continuous variables were presented as median (interquartile range, IQR: 25th to 75th quartile or minimum–maximum as applicable) when non-normally distributed and analyzed using the non-parametric Mann–Whitney U test or Kruskal-Wallis test, as appropriate. Univariate and multivariate logistic regression models were employed to ascertain independent prognostic factors affecting RFS, DMFS, and OS. The probability of RFS, DMFS, and OS was calculated via the Kaplan-Meier technique and compared across patient groupings using the log-rank test. Recurrence-free survival (RFS) was defined as the interval from the commencement of treatment to tumor recurrence or the final follow-up. Distant metastasis-free survival (DMFS) was defined as the duration from the commencement of treatment to the emergence of distant metastasis or the last follow-up date. Overall survival (OS) was defined as the period from diagnosis to death from any cause or the last follow-up appointment. All statistical studies utilized two-sided P values, with a threshold of <0.05 considered statistically significant. Statistical analyses were conducted utilizing the Statistical Package for the Social Sciences (SPSS Statistics for Windows, Version 25.0; IBM Corp., Armonk, NY, USA).

## Results

### Patient characteristics

The study included 35 patients with nasopharyngeal carcinoma (NPC), of which 14.3% were Epstein-Barr virus (EBV) negative and 85.7% were EBV positive. The cohort comprised 19 males and 16 females, with a mean age of 48.35 ± 18.10 years. Histological analysis, according to the World Health Organization (WHO) classification, revealed that 2 cases (5.7%) were keratinizing squamous cell carcinoma (KSCC), and 33 cases (94.3%) were non-keratinizing squamous cell carcinoma (NKSCC). According to the American Joint Committee on Cancer (AJCC) staging system, 33 patients (94.3%) were diagnosed at an advanced stage (stage III-IV), and 2 patients (5.7%) were detected at an early stage (stage II). The Surveillance, Epidemiology and End Results Program (SEER) classified 4 tumors (11.4%) as localized, 26 tumors (74.3%) as regional, and 5 tumors (14.3%) as distant metastases. The clinicopathological characteristics of the patients are summarized in **Table 1**.

**Table 1:** Demographic and clinical characteristics of the NPC patients.

Characteristics	Feature	All cases (n=35)	EBV− (n=5)	EBV+ (n=30)	*P* value
**Gender**	Male, n (%) Female, n (%)	19 (54.3) 16 (45.7)	2 (40) 3 (60)	17 (56.7) 13 (43.3)	0.489
**Age (years)**	Overall ≤ Mean age, n (%) > Mean age, n (%)	48.35±18.10 12 (34.3) 23 (65.7)	50.80±15.95 1 (20) 4 (80)	51.17±18.05 11 (36.7) 19 (63.3)	0.467
**BMI (kg/m²)**	Overall ≤ Mean BMI, n (%) > Mean BMI, n (%)	26.67 ±5.55 17 (48.6) 18 (51.4)	26.21±5.14 1 (20) 4 (80)	26.91±5.97 16 (53.3) 14 (46.7)	0.167
**EBV status**	EBV−, n (%) EBV+, n (%)	5 (14.3) 30 (85.7)	5 (100)	30 (100)	0
**WHO histological classification **	KSCC, n (%) NKSCC, n (%)	2 (5.7) 33 (94.3)	2 (40) 3 (60)	0 (0) 30 (100)	<0.001*
**Primary tumor (T) category**	T1, n (%) T2, n (%) T3, n (%) T4, n (%)	3 (8.6) 3 (8.6) 15 (42.9) 14 (40.0)	0 (0) 0 (0) 3 (60) 2 (40)	3 (10) 3 (10) 12 (40) 12 (40)	0.706
**Regional lymph nodes (N) category**	N0, n (%) N1, n (%) N2, n (%) N3, n (%)	4 (11.4) 8 (22.9) 19 (54.3) 4 (11.4)	1 (20) 1 (20) 2 (40) 1 (20)	3 (10) 7 (23.3) 17 (56.7) 3 (10)	0.804
**Distant metastasis category**	M0, n (%) M1, n (%)	30 (85.7) 5 (14.3)	5 (100) 0 (0)	25 (83.3) 5 (16.7)	0.324
**AJCC staging**	II, n (%) III, n (%) IVA, n (%) IVB, n (%)	2 (5.7) 15 (42.9) 13 (37.1) 5 (14.3)	0 (0) 2 (40) 3 (60) 0 (0)	2 (6.7) 13 (43.3) 10 (33.3) 5 (16.7)	0.573
**SEER staging**	Localized, n (%) Regional, n (%) Distant, n (%)	4 (11.4) 26 (74.3) 5 (14.3)	1 (20) 4 (80) 0 (0)	3 (10) 22 (73.3) 5 (16.7)	0.540
**Recurrence**	Yes, n (%) No, n (%)	5 (14.3) 30 (85.7)	0 (0) 5 (100)	5 (16.7) 25 (83.3)	0.324
**RFS (months)**	Overall Yes, n (%) No, n (%)	14 (10-35) 30 (85.7) 5 (14.3)	15 (9.5-39) 5 (100) 0 (0)	26 (6.75-34.25) 25 (83.3) 5 (16.7)	0.324
**DMFS (months)**	Overall Yes, n (%) No, n (%)	26 (5.5-30) 30 (85.7) 5 (14.3)	15 (9.5-39) 5 (100) 0 (0)	28 (6.75-34.25) 25 (83.3) 5 (16.7)	0.324
**OS (months)**	Overall Alive, n (%) Dead, n (%)	30 (10-35) 30 (85.7) 5 (14.3)	15 (9.5-39) 5 (100) 0 (0)	29.50 (6.75-35) 25 (83.3) 5 (16.7)	0.324

AJCC: American Joint Committee on Cancer; DMFS: Distant metastasis-free survival; KSCC: Keratinizing squamous cell carcinoma; NKSCC: Non-keratinizing squamous cell carcinoma; NPC: nasopharyngeal carcinoma; OS: overall survival; RFS: Recurrence-free survival; SEER: Surveillance, Epidemiology, and End Results Program.

Qualitative data are represented as the number of cases (%), whereas quantitative data are represented as mean ±SD (range, minimum-maximum) if normally distributed, or as median (range or interquartile range, IQR: 25^th^ quartile to 75^th^ quartile) if non-normally distributed. * indicates a statistical significant difference.

Qualitative data are represented as the number of cases (%), whereas quantitative data are represented as mean ± SD (range, minimum-maximum) if normally distributed, or as median (range or interquartile range, IQR: 25^th^ quartile to 75^th^ quartile) if non-normally distributed. * indicates a statistical significant difference.

### Expression of autophagy-related genes

**Figure 1:**
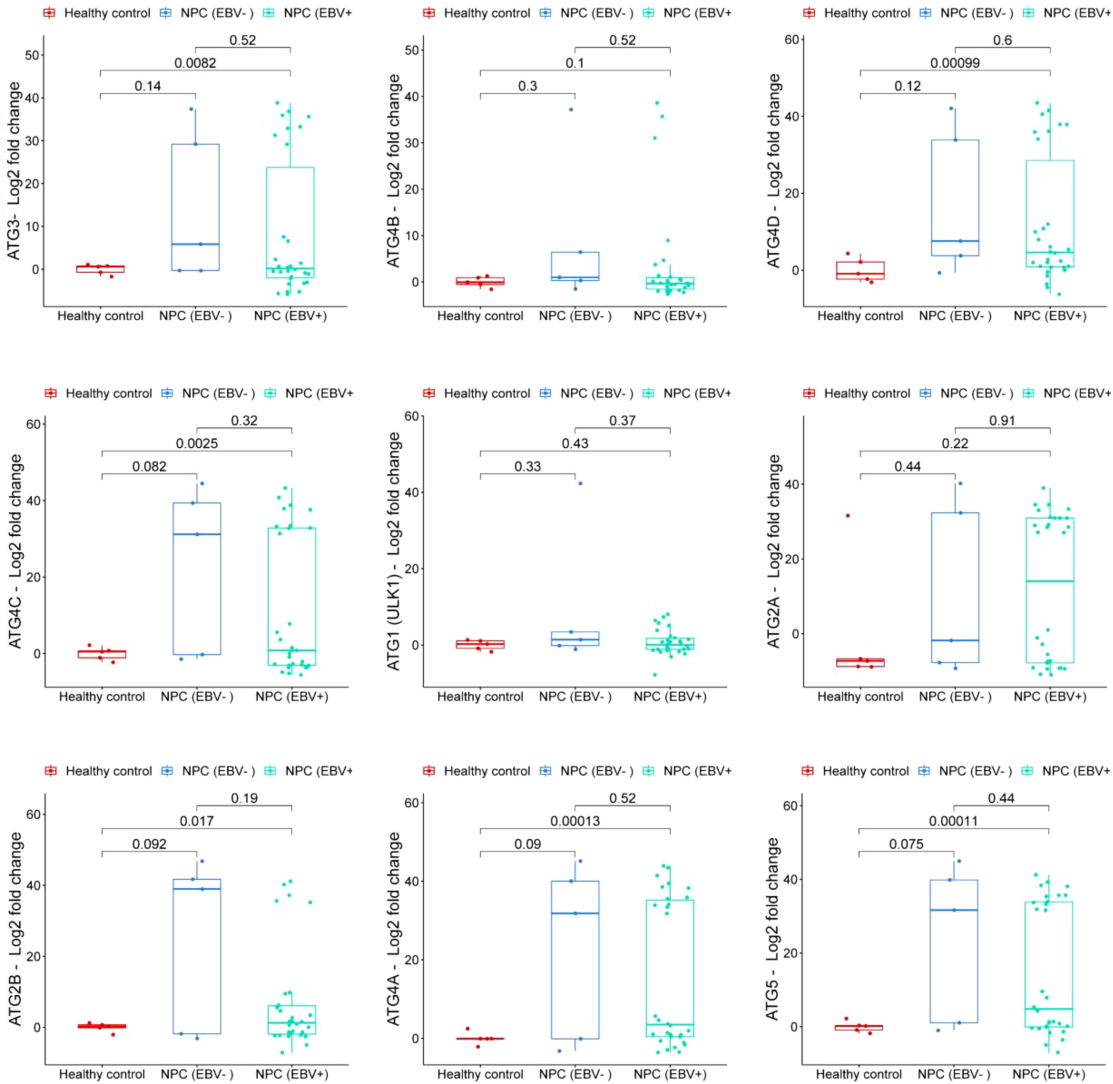
Comparison of Autophagy-Related Gene Expression. Expression profiles of autophagy-related genes in three groups: non-cancerous nasopharyngeal mucosal tissue samples (healthy controls); EBV-positive nasopharyngeal carcinoma tissue samples (NPC (EBV+)); and EBV-negative nasopharyngeal carcinoma tissue samples (NPC (EBV-)). The plot presents log2 fold change in gene expression, with p-values indicating statistical significance between the groups.

Expression levels of autophagy-related genes were analyzed in nasopharyngeal carcinoma (NPC) and patients categorized by Epstein-Barr virus (EBV) status compared to healthy controls. ATG3 expression was significantly higher in EBV-positive NPC compared to healthy controls, indicating moderate upregulation (p = 0.0082). Although ATG3 levels were also elevated in EBV-negative NPC compared to healthy controls, this difference was not significant (p = 0.14). These results suggest that ATG3 expression is notably increased in nasopharyngeal carcinoma, particularly in the EBV-positive group, indicating a potential role for EBV in modulating ATG3 levels (**Figure 1**). ATG4B exhibited a trend of increased expression in EBV-positive NPC compared to EBV-negative NPC, but this was not statistically significant (p = 0.52). Compared to healthy controls, ATG4B levels showed a moderate increase in EBV-positive NPC, although, again, not significant (p = 0.1) (**Figure 1**). ATG4D expression significantly increased compared to healthy controls (p = 0.00099), indicating substantial upregulation in EBV-positive NPC. While ATG4D levels were elevated in EBV-negative NPC, the difference compared to healthy controls was not significant (p = 0.12). This suggests that ATG4D is significantly higher in NPC, especially in the EBV-positive group, with complexities in how EBV influences ATG4D expression (**Figure 1**). ATG4C expression was significantly increased in EBV-positive NPC compared to healthy controls (p = 0.0025). Although ATG4C levels were elevated in EBV-positive NPC, the difference was not significant compared to EBV-negative NPC (p = 0.32). This highlights a significant elevation in ATG4C expression in NPC, particularly in the EBV-positive group, warranting further investigation into EBV’s role in ATG4C modulation (**Figure 1**). ATG1/ULK1 expression did not significantly increase in either EBV-negative or EBV-positive NPC compared to healthy controls (p = 0.33 and p = 0.43, respectively). The levels were slightly higher in EBV-positive NPC but not significant compared to EBV-negative NPC (p = 0.37) (**Figure 1**). ATG2A did not show a significant increase in EBV-negative NPC compared to healthy controls (p = 0.44), and expression levels were slightly higher in EBV-positive NPC compared to EBV-negative NPC, although not significant (p = 0.22) (**Figure 1**). ATG2B levels were significantly elevated in EBV-positive NPC compared to healthy controls (p = 0.017), but the difference compared to EBV-negative NPC was not significant (p = 0.19) (**Figure 1**). ATG4A showed increased expression in EBV-negative NPC compared to healthy controls, although this difference was not statistically significant (p = 0.09). In contrast, ATG4A expression was significantly elevated in EBV-positive NPC compared to healthy controls (p = 0.00013) (**Figure 1**). Lastly, ATG5 expression was significantly higher in EBV-positive NPC compared to healthy controls (p = 0.00011), indicating a marked elevation in this group. However, when comparing EBV-positive NPC to EBV-negative NPC, the difference was not significant (p = 0.44) (**Figure 1**). Overall, these results indicate that EBV may influence the expression of certain autophagy-related genes, particularly ATG3, ATG4D, and ATG4C in NPC.

### Expression of autophagy-related proteins

Normal nasopharyngeal mucosal tissue samples were analyzed through immunohistochemistry (IHC), and protein expression was absent for ATG1, ATG2B, ATG3, ATG4B, ATG4C, and ATG4D. However, ATG4A and ATG5 were expressed in 20% and 60% of cases, respectively. Notably, neither NPC tissues nor normal nasopharyngeal mucosal tissues exhibited positive protein expression for ATG2A.

Among the NPC patients, the counts of those exhibiting positive protein expression were as follows: ATG1 (32 patients, 91.43%); ATG2A (0 patients, 0%); ATG2B (23 patients, 65.71%); ATG3 (30 patients, 85.71%); ATG4A (33 patients, 94.29%); ATG4B (20 patients, 57.14%); ATG4C (22 patients, 62.86%); ATG4D (2 patients, 5.71%); and ATG5 (35 patients, 100%). Except for ATG4D, the positive expression rates for ATG1, ATG2B, ATG3, ATG4A, ATG4B, ATG4C, and ATG5 showed significant differences between NPC tissues and non-cancerous nasopharyngeal mucosa (P < 0.05) (**Table 2**). The protein levels did not show a consistent increase and varied between different samples. These results revealed distinct patterns between mRNA and protein expression levels. While mRNA levels exhibit a consistent increase, protein levels show variable expression levels.

**Table 2:** Expression of autophagy-related proteins in the study cohort.

Autophagy-related proteins	Immunoreactivity score	Non-cancerous (n=5)	Cancerous (n=35)	*P* value	Cancerous EBV− (n=5)	Cancerous EBV+ (n=30)	*P* value
ATG1	Negative, n (%)	5 (100)	3 (8.57)	<0.001*	1 (20)	2 (6.67)	0.324
	Positive, n (%)	0 (0)	32 (91.43)		4 (80)	28 (93.33)	
ATG2A	Negative, n (%)	5 (100)	35 (100)	–	5 (100)	30 (100)	–
	Positive, n (%)	0 (0)	0 (0)		0 (0)	0 (0)	
ATG2B	Negative, n (%)	5 (100)	12 (34.29)	0.005*	3 (60)	9 (30)	0.191
	Positive, n (%)	0 (0)	23 (65.71)		2 (40)	21 (70)	
ATG3	Negative, n (%)	5 (100)	5 (14.29)	<0.001*	3 (60)	2 (6.67)	0.002*
	Positive, n (%)	0 (0)	30 (85.71)		2 (40)	28 (93.33)	
ATG4A	Negative, n (%)	4 (80)	2 (5.71)	<0.001*	1 (20)	1 (3.33)	0.137
	Positive, n (%)	1 (20)	33 (94.29)		4 (80)	29 (96.67)	
ATG4B	Negative, n (%)	5 (100)	15 (42.86)	0.017*	4 (80)	11 (36.67)	0.07
	Positive, n (%)	0 (0)	20 (57.14)		1 (20)	19 (63.33)	
ATG4C	Negative, n (%)	5 (100)	13 (37.14)	0.008*	5 (100)	8 (26.67)	0.002*
	Positive, n (%)	0 (0)	22 (62.86)		0 (0)	22 (73.33)	
ATG4D	Negative, n (%)	5 (100)	33 (94.29)	0.583	5 (100)	28 (93.33)	0.552
	Positive, n (%)	0 (0)	2 (5.71)		0 (0)	2 (6.67)	
ATG5	Negative, n (%)	2 (40)	0 (0)	<0.001*	0 (0)	0 (0)	–
	Positive, n (%)	3 (60)	35 (100)		5 (100)	30 (100)	
ATG1	0 (negative), n (%)	5 (100)	3 (8.57)	<0.001*	1 (20)	2 (6.67)	0.181
	1 (weak positive), n (%)	0 (0)	0 (0)		0 (0)	0 (0)	
	2 (moderate positive), n (%)	0 (0)	2 (5.71)		1 (20)	1 (3.33)	
	3 (strong positive), n (%)	0 (0)	30 (85.71)		3 (60)	27 (90)	
ATG2A	0 (negative), n (%)	5 (100)	35 (100)	–	5 (100)	30 (100)	–
	1 (weak positive), n (%)	0 (0)	0 (0)		0 (0)	0 (0)	
	2 (moderate positive), n (%)	0 (0)	0 (0)		0 (0)	0 (0)	
	3 (strong positive), n (%)	0 (0)	0 (0)		0 (0)	0 (0)	
ATG2B	0 (negative), n (%)	5 (100)	12 (34.29)		3 (60)	9 (30)	0.423
	1 (weak positive), n (%)	0 (0)	8 (22.86)	0.052	0 (0)	8 (26.67)	
	2 (moderate positive), n (%)	0 (0)	13 (37.14)		2 (40)	11 (36.67)	
	3 (strong positive), n (%)	0 (0)	2 (5.71)		0 (0)	2 (6.67)	
ATG3	0 (negative), n (%)	5 (100)	5 (14.29)	0.001*	3 (60)	2 (6.67)	0.016*
	1 (weak positive), n (%)	0 (0)	6 (17.14)		0 (0)	6 (20)	
	2 (moderate positive), n (%)	0 (0)	22 (62.86)		2 (40)	20 (66.67)	
	3 (strong positive), n (%)	0 (0)	2 (5.71)		0 (0)	2 (6.67)	
ATG4A	0 (negative), n (%)	4 (80)	2 (5.71)	<0.0001*	1 (20)	1 (3.33)	0.034*
	1 (weak positive), n (%)	0 (0)	0 (0)		0 (0)	0 (0)	
	2 (moderate positive), n (%)	1 (20)	9 (25.71)		3 (60)	6 (20)	
	3 (strong positive), n (%)	0 (0)	24 (68.57)		1 (20)	23 (76.67)	
ATG4B	0 (negative), n (%)	5 (100)	15 (42.86)	0.126	4 (80)	11 (36.67)	0.296
	1 (weak positive), n (%)	0 (0)	5 (14.29)		0 (0)	5 (16.67)	
	2 (moderate positive), n (%)	0 (0)	10 (28.57)		1 (20)	9 (30)	
	3 (strong positive), n (%)	0 (0)	5 (14.29)		0 (0)	5 (16.67)	
ATG4C	0 (negative), n (%)	5 (100)	13 (37.14)	0.072	5 (100)	8 (26.67)	0.020*
	1 (weak positive), n (%)	0 (0)	14 (40)		0 (0)	14 (46.67)	
	2 (moderate positive), n (%)	0 (0)	7 (20)		0 (0)	7 (23.33)	
	3 (strong positive), n (%)	0 (0)	1 (2.86)		0 (0)	1 (3.33)	
ATG4D	0 (negative), n (%)	5 (100)	33 (94.29)	0.583	5 (100)	28 (93.33)	0.552
	1 (weak positive), n (%)	0 (0)	2 (5.71)		0 (0)	2 (6.67)	
	2 (moderate positive), n (%)	0 (0)	0 (0)		0 (0)	0 (0)	
	3 (strong positive), n (%)	0 (0)	0 (0)		0 (0)	0 (0)	
ATG5	0 (negative), n (%)	2 (40)	0 (0)	<0.0001*	0 (0)	0 (0)	–
	1 (weak positive), n (%)	0 (0)	0 (0)		0 (0)	0 (0)	
	2 (moderate positive), n (%)	0 (0)	0 (0)		0 (0)	0 (0)	
	3 (strong positive), n (%)	3 (60)	35 (100)		5 (100)	30 (100)	

Immunoreactivity (immunostaining) of autophagy-related proteins was scored based on the staining intensity level as follows: 0 (no staining, negative); 1 (light brown staining, weak positive); 2 (medium brown staining, moderate positive); 3 (dark brown staining, strong positive). Data are represented as the number of cases (%). * indicates a statistical significant difference.

**Figure 2:**
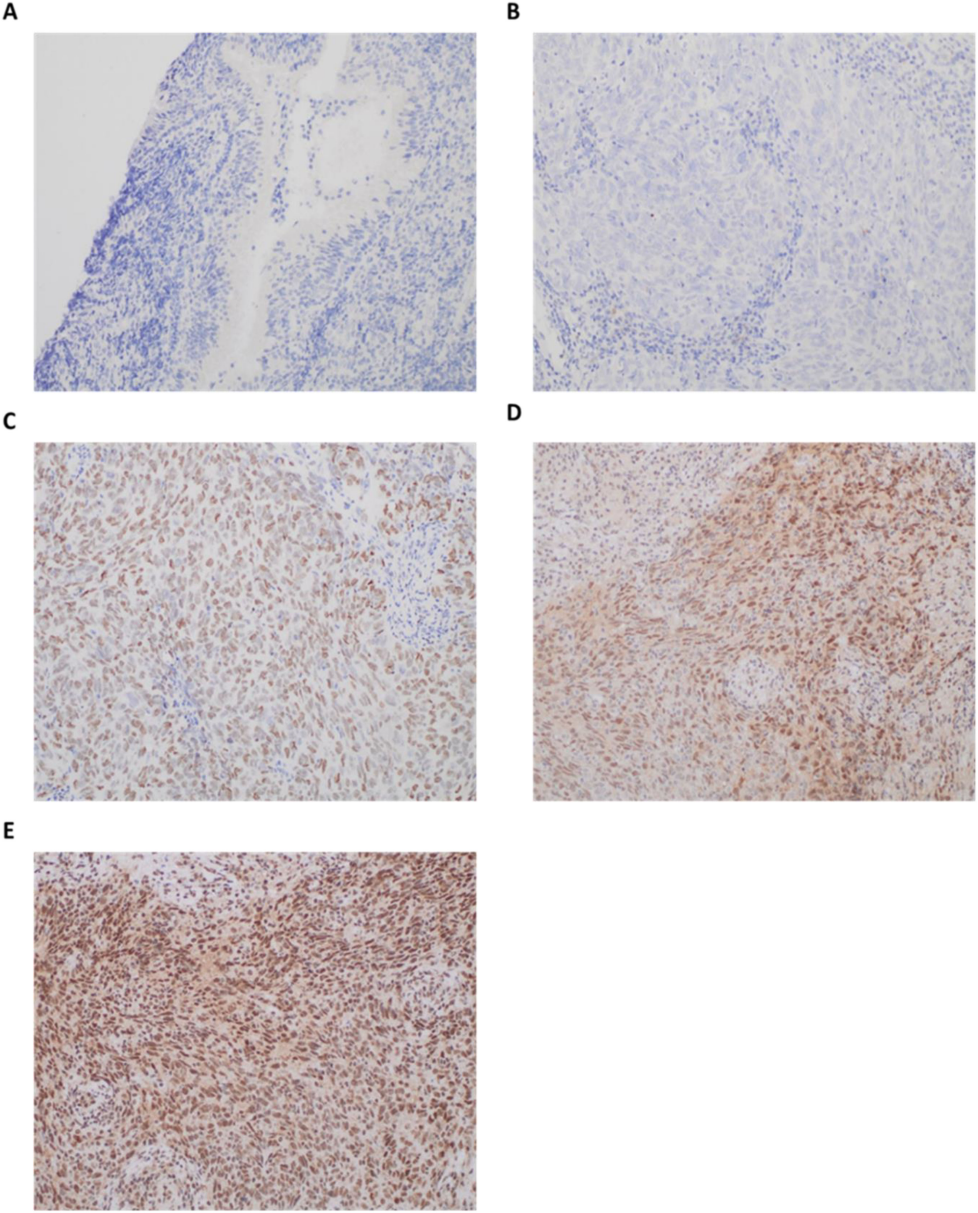
Representative immunohistochemical staining patterns of ULK1 protein expression in nasopharyngeal tissues. Scale bars are shown as approximate values (≈100 µm at 20×; ≈50 µm at 40×), based on typical field-of-view dimensions. **(A)** Negative ULK1 protein staining in normal nasopharyngeal mucosa (20×; scale bar = 100 µm); **(B)** Negative ULK1 protein staining in nasopharyngeal carcinoma (NPC) cells (40×; scale bar = 50 µm); **(C)** Weak expression of ULK1 protein in NPC cells (40×; scale bar = 50 µm); **(D)** Moderate expression of ULK1 protein in NPC cells (40×; scale bar = 50 µm); **(E)** Strong expression of ULK1 protein in NPC cells (40×; scale bar = 50 µm). The immunoreactivity of the autophagy-related proteins was evaluated based on staining intensity: no staining (negative); light brown staining (weak positive); medium brown staining (moderate positive); and dark brown staining (strong positive). Original magnification: 200×. Immunohistochemical staining was performed using Rabbit anti-Human ULK1 Polyclonal Antibody (Catalog #: MBS8574584, 1:50 dilution) as described in the Methods section.

In EBV-negative NPC patients, the positivity rates were: 80% for ATG1 (4 patients); 0% for ATG2A; 40% for ATG2B (2 patients); 40% for ATG3 (2 patients); 80% for ATG4A (4 patients); 20% for ATG4B (1 patient); and 0% for both ATG4C and ATG4D, while ATG5 was positive in 100% (5 patients). Conversely, in EBV-positive NPC patients, the positivity rates were as follows: ATG1 (28 patients, 93.33%); ATG2A (0 patients, 0%); ATG2B (21 patients, 70%); ATG3 (28 patients, 93.33%); ATG4A (29 patients, 96.67%); ATG4B (19 patients, 63.33%); ATG4C (22 patients, 73.33%); ATG4D (2 patients, 6.67%); and ATG5 (30 patients, 100%). Significant differences in positivity rates for ATG3 and ATG4C were observed between EBV-negative and EBV-positive NPC patients (P < 0.05) (**Table 2**). Representative images of ATG1 protein expression are displayed in **Figure 2**.

### Association between autophagy-related gene expression and clinicopathological features

The cohort was categorized into low (≤ median expression value) and high (> median expression value) subgroups based on the median mRNA expression levels. Detailed information regarding the frequencies of low and high autophagy-related gene expression in relation to various clinicopathological characteristics is provided in **Supplementary Tables S1**-**S9**.

The expression levels of ATG1, ATG2A, ATG2B, ATG4A, ATG4C, and ATG5 showed no significant correlation with the assessed clinicopathological features. In contrast, increased expression of ATG3 was significantly associated with a lower body mass index (BMI) in the entire cohort of NPC patients (P = 0.046), although it did not correlate with other patient characteristics. Furthermore, elevated expression levels of both ATG4B and ATG4D were more frequently observed in the No Distant Metastasis (M0) category compared to the Distant Metastasis (M1) category across the entire cohort of NPC patients (P = 0.019 for both ATG4B and ATG4D) and within the EBV-positive subgroup (P = 0.014 for both ATG4B and ATG4D). Within the EBV-positive subgroup, higher expression of ATG4B and ATG4D was more prevalent in regional carcinomas compared to localized or distant carcinomas (P = 0.031 for ATG4B; P = 0.048 for ATG4D), though this trend was not significant in the overall cohort (P = 0.061 for both ATG4B and ATG4D).

### Association between autophagy-related protein expression and clinicopathological characteristics

Based on the immunohistochemistry (IHC) results, the cohort was categorized into negative (absence of staining) and positive (light, medium, or dark brown staining) subgroups. Comprehensive data regarding the expression rates of negative and positive autophagy-related proteins in relation to various clinicopathological factors are summarized in **Supplementary Tables S10**-**S18**.

The expression of the proteins ATG1, ATG2A, ATG2B, ATG4B, and ATG5 showed no significant correlation with the evaluated clinicopathological characteristics. In contrast, positive expression of ATG3 and ATG4C was significantly more frequent in EBV-positive NPC patients compared to EBV-negative patients, with a strong correlation between positive expression of ATG3 or ATG4C and EBV positivity (P = 0.002 for both proteins). However, no correlations were found between positive ATG3 or ATG4C expression and other examined factors.

Positive expression of ATG4A was more prevalent in non-keratinizing squamous cell carcinoma (NKSCC) compared to keratinizing squamous cell carcinoma (KSCC) in the overall cohort of NPC patients (P = 0.005). Among the patients, ATG4A expression was observed more frequently in the N2 category compared to the N0, N1, and N3 categories, although this did not reach statistical significance (*P* = 0.321). However, within the EBV-positive subgroup, ATG4A expression was significantly higher in the N2 category (*P* = 0.025).

A statistically significant correlation was also identified between ATG4D protein expression and the primary tumor (T) category in both the overall cohort of NPC patients (*P* = 0.017) and the EBV-positive subgroup (*P* = 0.036).

### Association between autophagy-related genes/proteins expression and the clinical outcome of NPC patients

**Figure 3:**
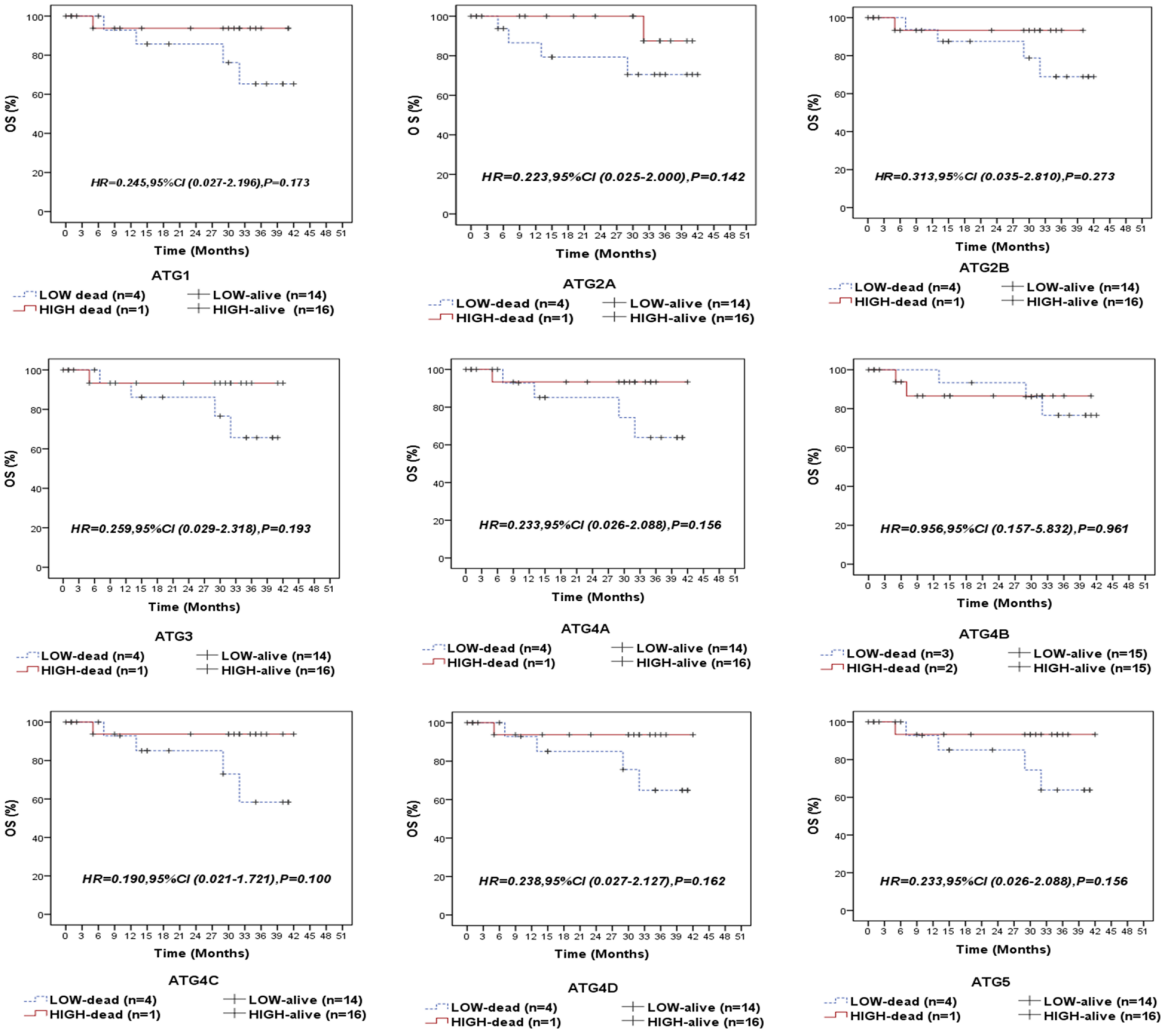
Overall survival (OS) of the entire series of NPC patients stratified by the expression status (low/high) of autophagy-related genes (ATG1, ATG2A, ATG2B, ATG3, ATG4A, ATG4B, ATG4C, ATG4D, and ATG5).

**Figure 4:**
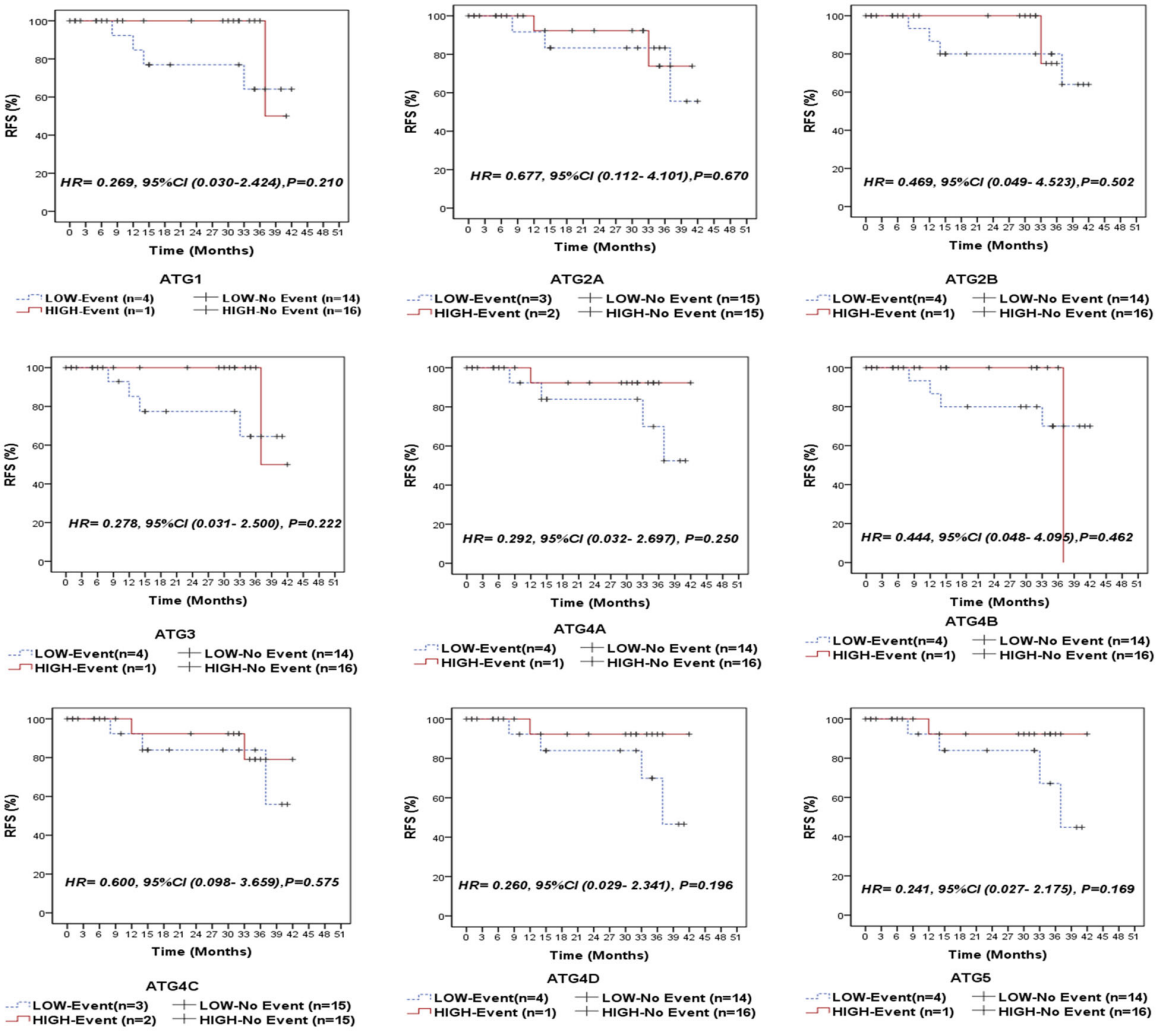
Recurrence-free survival (RFS) of the entire series of NPC patients stratified by the expression status (low/high) of autophagy-related genes (ATG1, ATG2A, ATG2B, ATG3, ATG4A, ATG4B, ATG4C, ATG4D, and ATG5).

Following a median follow-up period of 14 months (range: 5.5–35 months), 5 patients (14.3%) experienced relapse, 5 patients (14.3%) developed metastasis, and 5 patients (14.3%) died due to the disease. We compared the survival outcomes of NPC patients with high autophagy-related gene expression to those with low expression levels at the mRNA level. Kaplan-Meier survival analysis revealed that none of the examined genes was significantly associated with overall survival (OS) in the entire cohort of NPC patients (**Figure 3**) or in the EBV-positive subgroup (**Supplementary Figure S1**). Similarly, no significant associations were found between the analyzed genes and recurrence-free survival (RFS) in the overall cohort (**Figure 4**) or within the EBV-positive subgroup (**Supplementary Figure S2**).

**Figure 5:**
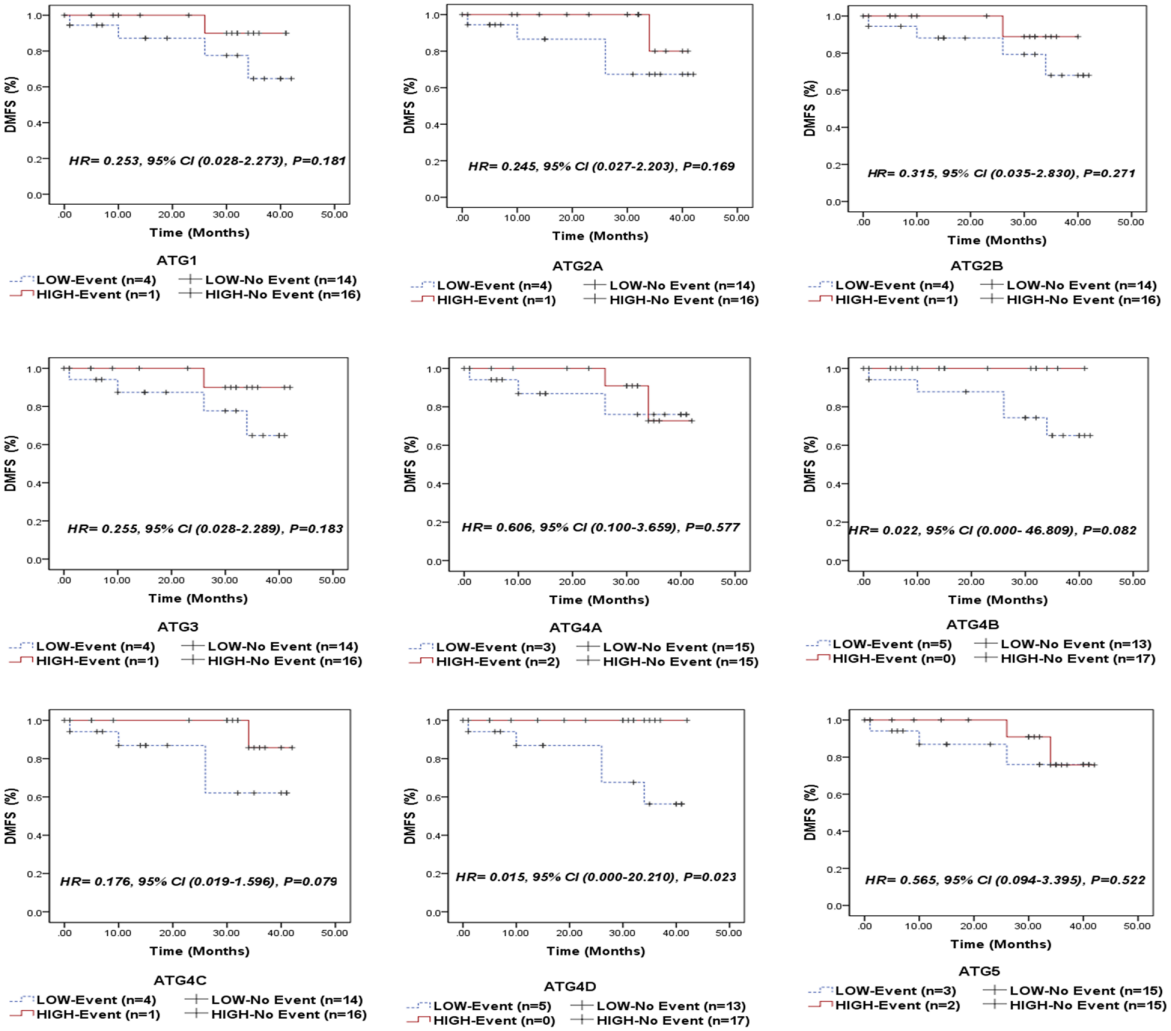
Distant metastasis-free survival (DMFS) of the entire series of NPC patients stratified by the expression status (low/high) of autophagy-related genes (ATG1, ATG2A, ATG2B, ATG3, ATG4A, ATG4B, ATG4C, ATG4D, and ATG5).

Of the analyzed genes, only ATG4D demonstrated a significant correlation with distant metastasis-free survival (DMFS) in both the overall cohort of NPC patients (P = 0.023, **Figure 5**) and the EBV-positive subgroup (P = 0.038, **Supplementary Figure S3**). This suggests that patients with elevated ATG4D expression had superior DMFS compared to those with lower levels.

We also evaluated survival outcomes at the protein level by comparing NPC samples with positive autophagy-related protein expression to those with negative expression. No significant differences were observed in OS (**Supplementary Figures S4**, **S5**), RFS (**Supplementary Figures S6**, **S7**), or DMFS (**Supplementary Figures S8**, **S9**) between the positive and negative patient groups for all examined autophagy-related proteins, both in the entire cohort and in the EBV-positive subgroup. The absence of ATG2A protein expression in all cases prevented the execution of Kaplan-Meier survival analysis comparing the “negative” and “positive” expression cohorts.

## Discussion

Autophagy-related genes (ATGs) play a pivotal role in the biology of NPC. Autophagy is a cellular process that degrades and recycles cellular components, thereby maintaining homeostasis and responding to stress. In the context of NPC, ATGs are crucial for several reasons. They may regulate tumor cell survival and proliferation. Alterations in autophagy can disrupt these processes, contributing to tumorigenesis and tumor progression therapy resistance, and immune evasion underscoring their importance in NPC biology. This study aimed to investigate the expression levels of autophagy-related genes (ATGs) at both the mRNA and protein levels in nasopharyngeal carcinoma (NPC) and their association with EBV status, clinicopathological characteristics, and clinical outcomes. These specific autophagy-related genes were selected based on several critical considerations. First, they represent key components across the sequential stages of the autophagy process: ATG1/ULK1 functions in the initiation phase as a serine/threonine kinase that forms the ULK complex; ATG2A and ATG2B are essential for autophagosome formation and lipid transfer between the endoplasmic reticulum and phagophore; ATG3 serves as an E2-like enzyme crucial for LC3 lipidation during autophagosome elongation; the ATG4 family (ATG4A, ATG4B, ATG4C, and ATG4D) comprises cysteine proteases responsible for processing pro-LC3 into its active form and recycling LC3 from autophagosome membranes; and ATG5 forms part of the ATG5-ATG12-ATG16L1 complex necessary for autophagosome membrane elongation. Second, previous studies have specifically implicated these genes in viral infection responses and EBV-mediated pathogenesis. Third, these genes have demonstrated clinical relevance in nasopharyngeal and other EBV-associated malignancies (17). Our results revealed that ATG3, ATG4D, ATG4C, ATG4A, ATG2B, ATG5 expression was significantly higher in EBV-positive NPC compared to healthy controls, suggesting a notable role of EBV in modulating the levels of these genes. Other ATGs, including ATG4B, ATG1 and ATG2A, did not show significant increases in either NPC subtype. These findings highlight the intricate interplay between EBV and ATGs expression in NPC, suggesting potential implications for therapeutic strategies targeting autophagy in EBV-associated malignancies. The mRNA levels of ATG4D were significantly correlated with higher rates of distant metastasis in the overall cohort of NPC patients and specifically within the EBV-positive subgroup. Patients with low ATG4D gene expression demonstrated a significant trend toward shorter distant metastasis-free survival (DMFS) compared to those with high ATG4D expression, both in the entire cohort and in the EBV-positive subgroup. However, at the protein level, no significant associations were found between the expression of any of the examined proteins and overall survival (OS), recurrence-free survival (RFS), or DMFS.

The progression of autophagy encompasses numerous sequential steps that are meticulously regulated. Induction of autophagy in response to external stimuli, such as nutrient scarcity, activates the ATG1 kinase complex, comprising ATG1, ATG13 (a regulatory protein), and the ATG17-ATG29-ATG31 scaffold subcomplex, through the inhibition of Target of Rapamycin Complex 1 (TORC1) and/or the activation of AMP-activated protein kinase (AMPK). This complex attracts more ATG proteins to the phagophore assembly sites (PAS) and activates downstream proteins by phosphorylation. Thereafter, a class III Ptdlns3K complex I (PI3KC3), comprising Vps34 (lipid kinase), Vps15 (regulatory kinase), Vps30/Atg6, Atg14, and Atg38, is recruited to the phagophore’s PAS. The phosphorylation of PI3 by this complex at the PAS facilitates the recruitment of ATG18 and ATG2, which are essential for the attachment of ATG8, ATG9, and ATG12 to the PAS. ATG12 and ATG8 are two critical ubiquitin-like proteins necessary for the production of autophagosomes. ATG12 is conjugated to ATG5 by the action of ATG7 (an E1-like enzyme) and ATG10 (an E2-like enzyme). The conjugate then associates with ATG16L to create a dimeric ATG12-ATG5-ATG16L E3-like enzyme for ATG8. This is subsequently covalently linked to the lipid phosphatidyl-ethanolamine (ATG8-PE) by the actions of ATG4 (protease), ATG7 (E1-like enzyme), ATG3 (E2-like enzyme), and the dimeric ATG12-ATG5-ATG16L complex (E3-like enzyme). Lipidated ATG8 facilitates membrane elongation, substrate recruitment, and, ultimately, the development of the double membrane-enclosed autophagosome. Subsequently, ATG8-PE is cleaved by ATG4 from the outer autophagosomal membrane to commence the fusion of autophagosomes and lysosomes for the destruction of substrates and the inner autophagosome membrane (17). The relationship between EBV and autophagy has emerged as a critical area in cancer research, particularly in EBV-associated malignancies like NPC. EBV has evolved sophisticated mechanisms to manipulate the autophagy pathway for its benefit throughout its lifecycle. During primary infection, EBV must evade xenophagy, a selective form of autophagy that targets intracellular pathogens. Studies have demonstrated that EBV viral proteins, particularly BILF1 and LMP1, can inhibit autophagosome formation during early infection, preventing viral clearance. In the latent phase, which predominates in NPC, EBV establishes a complex relationship with autophagy. Latent membrane protein 1 (LMP1), the principal oncoprotein of EBV, has been shown to both activate and inhibit autophagy depending on the cellular context. In epithelial cells, LMP1 can induce autophagy through activation of the unfolded protein response (UPR) and JNK signaling pathways. This LMP1-induced autophagy has been suggested to promote cell survival by recycling nutrients and removing harmful cellular components, thereby supporting tumor growth. Conversely, LMP1 can also inhibit autophagic flux at late stages to prevent its degradation, creating a form of “incomplete autophagy” that favors viral persistence and oncogenesis. Latent membrane protein 2A (LMP2A), another key EBV protein expressed in NPC, has been shown to activate the PI3K/Akt/mTOR pathway, a major negative regulator of autophagy. Through this mechanism, LMP2A can suppress autophagy initiation, potentially contributing to the accumulation of cellular damage and genomic instability characteristic of cancer progression. EBV-encoded small RNAs (EBERs), which are abundantly expressed in EBV-positive NPC, have been linked to autophagy modulation through interaction with pattern recognition receptors such as RIG-I and TLR3, leading to type I interferon production and subsequent autophagy induction (18, 19). This complex signaling cascade represents another layer in the intricate relationship between EBV and the autophagy machinery. Recent studies have revealed that EBV-positive cancers, including NPC, often display distinctive autophagy signatures compared to their EBV-negative counterparts. Genomic analyses have identified recurrent mutations and alterations in autophagy-related genes in EBV-associated malignancies, suggesting selective pressure for autophagy dysregulation during EBV-mediated oncogenesis. Furthermore, epigenetic modifications induced by EBV, particularly through the action of LMP1 and EBNA1, have been shown to influence the expression of key autophagy regulators, creating an “autophagy landscape” that favors viral persistence and tumor progression. The therapeutic implications of these interactions are significant. EBV-positive cancers have shown differential responses to autophagy modulators compared to EBV-negative tumors. For instance, chloroquine derivatives, which inhibit autophagosome-lysosome fusion, have demonstrated enhanced efficacy against EBV-positive lymphomas by disrupting the delicate autophagy balance maintained by the virus. Similarly, rapamycin analogs, which induce autophagy through mTOR inhibition, have shown promising results in preclinical models of EBV-associated malignancies by potentially promoting complete autophagic flux and viral clearance (20, 21).

The intricate relationship between EBV and autophagy presents significant implications for our understanding of viral pathogenesis and cancer biology. Building on the current understanding of autophagy in EBV-positive cancers discussed above, our findings provide additional insights into this complex relationship in the context of NPC. The prevailing evidence suggests that EBV, through its encoded proteins, tends to suppress autophagy during both *de novo* infection and reactivation from latency, thereby facilitating immune evasion and promoting oncogenic processes. This suppression undermines the protective role of autophagy in cellular homeostasis, particularly in the context of reactive oxygen species management and organelle integrity, which are critical in preventing neoplastic transformation. Furthermore, the dual role of autophagy in both supporting viral replication and influencing cancer progression underscores its complexity as a biological process (18, 19). The potential for pharmacological modulation of autophagy presents a promising therapeutic avenue for managing EBV-associated malignancies, particularly when combined with apoptosis-inducing agents (20, 21). Future research should focus on elucidating the precise mechanisms by which EBV manipulates autophagy, as well as exploring the therapeutic efficacy of autophagy modulators in clinical settings, to enhance treatment strategies for patients with EBV-related cancers. Several EBV-related proteins have been found to interact with autophagy-related genes (ATGs) and influence their activity. LMP1 is one of the most studied EBV proteins and acts as a functional homolog of a CD40 receptor. It can activate multiple signaling pathways, including NF-κB and MAPK, which are involved in the regulation of autophagy. Studies suggest that LMP1 can promote the expression of certain ATGs, enhancing autophagy and potentially aiding in viral persistence (1, 22). LMP2A has been shown to interfere with cellular signaling pathways that regulate autophagy. It can modulate the PI3K/Akt/mTOR pathway, potentially impacting the levels and activity of ATGs, although the specifics of its interactions with individual ATGs require further investigation (2, 3). BHRF1 has been implicated in the regulation of apoptosis and autophagy. It can modulate the expression of various proteins involved in these pathways, potentially affecting ATG expression and activity (5). EBERs have been associated with the modulation of cellular responses, including autophagy. Their exact role in directly interacting with ATGs is still under investigation, but they are known to influence the host’s immune response and can indirectly affect autophagic processes (6, 7).

The ATG4A demonstrated increased expression in EBV-negative NPC compared to healthy controls. In contrast, ATG4A expression was significantly elevated in EBV-positive NPC, compared to healthy controls. This suggests that while ATG4A may play a role in both subtypes of NPC, its expression is particularly pronounced in the presence of EBV, indicating a potential interaction between EBV and autophagy regulation (8, 10). EBV can alter the immune response in infected cells, which might indirectly influence ATG4A expression. For instance, by evading immune detection, EBV-infected cells may rely more on autophagy for survival, leading to increased ATG4A levels. Increased autophagy driven by ATG4A can create a feedback loop where the virus further alters host cell pathways to maintain a favorable environment for its replication and persistence. This interaction may help the EBV evade the immune response and promote tumor survival (11). These findings suggest that ATG4A expression is significantly altered in nasopharyngeal carcinoma, particularly in the presence of EBV, which may have implications for understanding the role of autophagy in tumor biology and potential therapeutic strategies. ATG4B exhibited a trend of increased expression in EBV-positive NPC, compared to EBV-negative NPC. These findings suggest that while there may be a tendency for ATG4B to be upregulated in the context of EBV, further investigation is needed to clarify its role (12, 17). ATG3 expression was significantly higher in EBV-positive NPC compared to healthy controls, indicating moderate upregulation. ATG3 levels were also elevated in EBV-negative NPC compared to healthy controls. This suggests that ATG3 expression is notably increased in NPC, particularly in the EBV-positive group, highlighting a potential role for EBV in modulating ATG3 levels (12, 17, 18).

In this study, the expression levels of autophagy-related genes in EBV-positive NPC patients were analyzed, revealing higher mRNA expression levels of ATG1 in 13 EBV-positive NPC patients compared to EBV-negative patients, suggesting a potential role for ATG1 in tumor progression. Additionally, ATG2A showed elevated expression in 18 patients, further emphasizing the connection between EBV positivity and autophagy regulation. The highest expression was noted for ATG3 in 21 EBV-positive NPC patients, highlighting its potential as a key player in modulating autophagy in the context of EBV infection. Expression levels of ATG4A were observed in 11 patients, suggesting some modulation by EBV. Six patients exhibited expression of ATG4B, indicating a lesser role compared to other ATGs in NPC. Similar to ATG4A, ATG4D was expressed in 11 patients. Lastly, 9 patients showed expression of ATG5, reinforcing its potential involvement in autophagy processes in NPC. Overall, these results indicate that EBV-positive NPC patients demonstrate higher mRNA expression levels of certain autophagy-related genes, suggesting a role of EBV in modulating autophagy-related gene expression in NPC.

ATG4D expression levels were elevated in EBV-positive NPC; however, the difference compared to EBV-negative NPC was not significant. This suggests that ATG4D is significantly higher in NPC, especially in the EBV-positive group, indicating complexities in how EBV influences ATG4D expression (16, 17). The role of ATG4D in the pathogenesis of EBV-induced NPC presents significant implications for both therapeutic strategies and disease prognostic assessments. As a key regulator of autophagy, ATG4D influences cellular survival and proliferation, particularly in the context of viral infections. Elevated ATG4D expression has been associated with advanced disease and poorer survival rates, suggesting an association with OS, RFS, or DMFS.

Therapeutic Target: Targeting ATG4D could provide a novel approach to enhance the efficacy of existing treatments for NPC. Inhibiting ATG4D may impair the autophagic process that allows NPC cells to thrive, particularly in the presence of EBV, potentially leading to increased sensitivity to chemotherapy and radiotherapy. Further research into specific inhibitors of ATG4D could pave the way for innovative combination therapies that exploit this vulnerability.

ATG4C expression was significantly increased in EBV-positive NPC compared to healthy controls. Although ATG4C levels were elevated in EBV-negative NPC, the difference was not significant compared to healthy controls. This highlights a significant elevation in ATG4C expression in NPC, particularly in the EBV-positive group, warranting further investigation into EBV’s role in ATG4C modulation (16, 19).

ATG5 expression was significantly higher in EBV-positive NPC compared to healthy controls. However, when comparing EBV-positive NPC to EBV-negative NPC, the difference was not significant. This suggests that while ATG5 is upregulated in the presence of EBV, its role may not differ significantly between the two NPC subtypes (18, 20, 21). ATG2B levels were significantly elevated in EBV-positive NPC compared to healthy controls, but the difference compared to EBV-negative NPC was not significant. This indicates a potential role for ATG2B in EBV-positive NPC, although further studies are needed to elucidate its function (12, 13). ATG1 and ATG2A expression did not significantly increase in either EBV-negative or EBV-positive NPC compared to healthy controls, indicating that ATG1 may not play a significant role in the context of NPC (14). LMP1, for instance, can activate pathways like NF-κB, which may lead to changes in autophagy-related gene expression, including ATG4A, ATG4B, ATG3, ATG4D, ATG4C, ATG5, ATG2B, ATG2A and ATG1 (9, 11, 20). Neither nasopharyngeal carcinoma (NPC) nor normal nasopharyngeal mucosal tissues exhibited positive expression of the ATG2A protein. The prevalence of positive expression for autophagy-related proteins among NPC patients was as follows: ATG1 (91.43%); ATG2A (0%); ATG2B (65.71%); ATG3 (85.71%); ATG4A (94.29%); ATG4B (57.14%); ATG4C (62.86%); ATG4D (5.71%); and ATG5 (100%). Except for ATG4D, the positive expression rates of ATG1, ATG2B, ATG3, ATG4A, ATG4B, ATG4C, and ATG5 showed significant differences between NPC tissues and non-cancerous nasopharyngeal mucosal tissues.

In EBV-negative NPC patients, the positivity rates were as follows: ATG1 (80%); ATG2A (0%); ATG2B (40%); ATG3 (40%); ATG4A (80%); ATG4B (20%); ATG4C (0%); ATG4D (0%); and ATG5 (100%). Conversely, in EBV-positive NPC patients, the positivity rates for the same proteins were: ATG1 (93.33%); ATG2A (0%); ATG2B (70%); ATG3 (93.33%); ATG4A (96.67%); ATG4B (63.33%); ATG4C (73.33%); ATG4D (6.67%); and ATG5 (100%). Notably, only the positivity rates of ATG3 and ATG4C exhibited significant differences between EBV-negative and EBV-positive NPC patients. Post-translational modifications may contribute to variations in the expression of autophagy-related proteins (23–25). The differing expression patterns observed among our patient cohort highlight the complex autophagic environment in NPCs, suggesting that NPC may have multiple components of the autophagy machinery.

The results of autophagy-related gene expression indicate that EBV may significantly influence the levels of specific autophagy-related genes, particularly ATG3, ATG4D, and ATG4C, which are notably elevated in EBV-positive NPC patients. In contrast, the expression of autophagy-related proteins shows a marked difference between NPC and normal tissues, with proteins such as ATG1 and ATG5 being highly expressed in NPC. However, the protein levels did not exhibit a consistent increase and varied across different samples. The absence of ATG2A expression in NPC may serve as a potential biomarker for distinguishing cancerous from non-cancerous tissues. Our analysis revealed distinct patterns between mRNA and protein expression levels. While mRNA levels consistently increase in response to treatment, protein levels demonstrate variable responses. This discrepancy suggests that post-transcriptional modifications play a significant role in regulating protein synthesis. Understanding these differences is essential for accurately interpreting biological outcomes and optimizing therapeutic strategies.

No notable differences were observed at the protein level for any of the assessed autophagy-related proteins OS, RFS, or DMFS, between the positive and negative patient cohorts, both in the complete cohort of NPC patients and the EBV-positive subgroup. However, the Kaplan-Meier survival analysis at the mRNA level indicated that none of the analyzed genes exhibited a significant correlation with OS or RFS in the entire cohort of NPC patients or the EBV-positive subgroup. Of the examined genes, only ATG4D had a significant correlation with DMFS in the total cohort of NPC patients and the EBV-positive subgroup, indicating that patients with heightened ATG4D expression levels experienced improved DMFS outcomes relative to those with reduced ATG4D expression. This finding contrasts with previous reports suggesting that increased ATG4D expression is linked to tumor progression and resistance to therapy, which may impact DMFS (26). The discrepancy may be attributed to differences in the tumor microenvironment, genetic backgrounds, or the characteristics of the study cohort. While previous studies have indicated that ATG4D expression can be influenced by the tumor microenvironment, which plays a significant role in cancer metastasis (26, 27). Our results suggest a potentially protective role for ATG4D in NPC. Changes in autophagy regulation, which affect tumor cell interactions with their surroundings and immune evasion, have been highlighted as mechanisms by which ATG4D might influence metastasis (27). Autophagy-related proteins, including ATG4D, have been associated with the regulation of EMT and interactions with key signaling pathways, such as mTOR, that regulate cancer progression (27, 28). These discrepancies emphasize the need for further investigations to clarify the molecular mechanisms of ATG4D in NPC, and assess how targeting this gene might influence DMFS and overall treatment outcomes. The role of Unc-51-Like Autophagy Activating Kinase 1 (ULK1/ATG1) in the regulation of autophagy presents a critical area of investigation, particularly in the context of nasopharyngeal carcinoma (NPC). The dual regulatory mechanisms of ATG1, influenced by nutrient availability and cellular energy status, highlight its significance in both the initiation of autophagy and the survival of malignant cells under stress conditions. Elevated ATG1 expression has been consistently associated with poor clinical outcomes, highlighting its potential association with aggressive tumor behavior and treatment resistance (25, 29, 30). The correlation between increased ATG1 levels and advanced disease stages, as well as the propensity for metastasis, further emphasizes its role in the pathology of NPC. The implications of ATG1 in the development of chemo/radio-resistance suggest that targeting this autophagy-related kinase could offer novel therapeutic strategies in managing NPC (31, 32).

Autophagy can have dual roles in cancer: it can act as a tumor suppressor by removing damaged organelles and proteins, but it can also support cancer cell survival by providing nutrients during stress conditions. In EBV-positive cancers, autophagy may help the virus persist within host cells and evade immune responses. Several studies showed that autophagy is an important mechanism in EBV-positive malignancies. EBV-positive latency stage III B-cell lymphoproliferations displayed constitutively elevated autophagy levels that may be a mechanism of apoptosis-resistance (33). Autophagy was enhanced during EBV lytic activation in Burkitt’s lymphoma cells, but was subsequently suppressed by early viral antigens. Inhibiting autophagy increases EBV lytic gene expression and viral replication (34). In Hodgkin lymphoma, EBV latent membrane protein-1 (LMP1) upregulates autophagy, promoting cell viability and attenuating starvation-induced autophagic stress (35). The EBV oncoprotein EBNA3C elevates autophagy gene transcription through epigenetic modifications, particularly under growth-limiting conditions. This increase had a protective effect, as it could suppress apoptosis and sustain cell growth in EBV-related B-cell lymphomas (36). Autophagy initiation, particularly the ATG5 protein, was required for EBV lytic reactivation in nasopharyngeal carcinoma (37). The EBV-encoded Rta activates autophagy to enhance EBV lytic development, which leads to an increase in the level of autophagy within EBV-positive cells (38). These findings highlight the complex dynamics of the relationship between EBV and autophagy in carcinogenesis.

Despite the originality of our findings, two main limitations must be acknowledged. Firstly, the sample size (n=35 for NPC, n=5 controls) was relatively small, which may have limited the statistical power of our analyses and potentially affected the robustness of some of our findings, particularly regarding the relationships between ATG expression and clinical outcomes. This limitation underscores the need for validation studies with larger cohorts to confirm the prognostic significance of the ATGs identified in our study. Secondly, our study was a single-center design, which may limit the generalizability of our results. Therefore, additional studies involving multicenter cohorts with diverse patient populations are necessary to validate our findings and ensure their applicability across different geographic and demographic contexts. Secondly, our study was a single-center design. Therefore, additional studies involving multicenter cohorts are needed.

Based on our findings and the limitations identified, we propose a comprehensive roadmap for future research: 1) Larger cohort studies: Multicenter prospective studies with larger sample sizes (n > 200) are needed to validate the prognostic significance of ATG4D and other ATGs in NPC. These studies should include stratification by EBV status and standardized clinical follow-up protocols; 2) Functional mechanistic studies: *In vitro* and *in vivo* experiments should be conducted to elucidate the precise molecular mechanisms by which ATG4D influences distant metastasis in NPC. This should include genetic manipulation (knockdown/overexpression) of ATG4D in NPC cell lines, analysis of downstream signaling pathways affected by ATG4D alterations, and investigation of the interaction between ATG4D and EBV proteins, particularly LMP1 and LMP2A; 3) Therapeutic targeting studies: Exploration of ATG4D as a potential therapeutic target through development and testing of specific ATG4D inhibitors or activators, combination approaches with standard chemoradiotherapy protocols, and assessment of synergistic effects with other autophagy modulators; 4) Comprehensive multi-omics analysis: Integration of transcriptomics, proteomics, and metabolomics data to provide a more complete understanding of the autophagy landscape in NPC and its relationship with EBV infection; 5) Liquid biopsy development: Investigation of ATG4D expression in circulating tumor cells or cell-free DNA as a non-invasive biomarker for monitoring disease progression and treatment response; 6) Immunological studies: Examination of the relationship between autophagy-related genes, particularly ATG4D, and the tumor immune microenvironment in NPC, including implications for immunotherapy response.

Our findings have several important clinical implications that warrant consideration in the management of NPC patients: 1) Prognostic stratification: The identification of a potential association of distant metastasis with ATG4D suggests its utility in risk stratification of NPC patients. Low ATG4D expression could identify high-risk patients who might benefit from more aggressive treatment approaches or closer surveillance protocols; 2) Personalized treatment approaches: The differential expression of ATGs based on EBV status indicates that therapeutic strategies targeting autophagy might need to be tailored according to the EBV status of the tumor. This personalized approach could enhance treatment efficacy and reduce unnecessary toxicity; 3) Development of novel therapeutics: Our findings suggest that modulators of autophagy, particularly those targeting ATG4D, could represent a novel class of therapeutic agents for NPC. These could potentially be developed as adjuvants to current standard treatments to improve outcomes, especially in patients at high risk for distant metastasis; 4) Surveillance strategies: Integration of ATG4D expression analysis into post-treatment surveillance protocols could help identify patients at higher risk of distant metastasis, allowing for earlier intervention and potentially improved outcomes; 5) Combined ATGs panels: The complex expression patterns of multiple ATGs observed in our study suggest that a panel approach, rather than single ATG testing, might provide more comprehensive prognostic information. This could involve combining ATG4D with other established biomarkers in NPC.

This study highlights the significant association between autophagy-related gene expression in EBV-positive NPC patients. To further investigate these relationships, future research should focus on conducting multicenter studies with larger cohorts to validate our findings and implement longitudinal studies to track ATG expression over time. Mechanistic studies exploring how specific EBV proteins interact with autophagy pathways are essential to elucidate their roles in cancer progression. Additionally, assessing the therapeutic efficacy of autophagy modulators in combination with standard treatments could provide novel strategies for managing EBV-associated malignancies. The observed correlation between ATG4D expression and distant metastasis-free survival suggests that ATG4D could serve as a prognostic biomarker, helping in identifying patients at higher risk for metastasis and tailoring treatment strategies accordingly. Overall, these insights underscore the need for further exploration of autophagy’s role in NPC to enhance clinical outcomes and develop targeted therapeutic approaches. While the expression patterns of autophagy-related genes provide valuable insights into the intricate interplay between EBV and autophagy in NPC, the absence of significant correlations at the protein level underscores the need for further investigation into the roles of these proteins in clinical outcomes. Future research is crucial to validate these findings and explore the implications of autophagy-related genes for targeted therapeutic strategies. Given the role of EBV in modulating autophagy, targeting autophagy pathways may offer novel therapeutic avenues for managing EBV-associated malignancies. Pharmacological modulation of autophagy, particularly in conjunction with existing treatments, could enhance therapeutic efficacy and overcome resistance.

## Data Availability

The raw data supporting the conclusions of this article will be made available by the authors, without undue reservation.

## References

[1] Wong KC Hui EP Lo K-W Lam WKJ Johnson D Li L. Nasopharyngeal carcinoma: an evolving paradigm. Nat Rev Clin Oncol. (2021) 18:679–95. doi: 10.1038/s41571-021-00524-x 34194007

[2] Bray F Laversanne M Sung H Ferlay J Siegel RL Soerjomataram I. Global cancer statistics 2022: GLOBOCAN estimates of incidence and mortality worldwide for 36 cancers in 185 countries. CA: Cancer J Clin. (2024) 74:229–63. doi: 10.3322/caac.21834 38572751

[3] Vasudevan HN Yom SS. Nasopharyngeal carcinoma and its association with Epstein-Barr virus. Hematology/Oncology Clinics. (2021) 35:963–71. doi: 10.1016/j.hoc.2021.05.007 34187713

[4] Al-Anazi AE Alanazi BS Alshanbari HM Masuadi E Hamed ME Dandachi I. Increased prevalence of EBV infection in nasopharyngeal carcinoma patients: a Six-Year Cross-Sectional study. Cancers. (2023) 15:643. doi: 10.3390/cancers15030643 36765601 PMC9913071

[5] Perri F Scarpati GDV Giuliano M D’Aniello C Gnoni A Cavaliere C. Epstein–Barr virus infection and nasopharyngeal carcinoma: the other side of the coin. Anti-cancer Drugs. (2015) 26:1017–25. doi: 10.1097/CAD.0000000000000276 26241803

[6] Mizushima N Levine B Cuervo AM Klionsky DJ. Autophagy fights disease through cellular self-digestion. nature. (2008) 451:1069–75. doi: 10.1038/nature06639 PMC2670399 18305538

[7] Levine B Kroemer G. Autophagy in the pathogenesis of disease. Cell. (2008) 132:27–42. doi: 10.1016/j.cell.2007.12.018 18191218 PMC2696814

[8] Mizushima N Levine B. Autophagy in human diseases. New Engl J Med. (2020) 383:1564–76. doi: 10.1056/NEJMra2022774 33053285

[9] Deretic V Saitoh T Akira S. Autophagy in infection, inflammation and immunity. Nat Rev Immunol. (2013) 13:722–37. doi: 10.1038/nri3532 PMC5340150 24064518

[10] Granato M Santarelli R Farina A Gonnella R Lotti LV Faggioni A. Epstein-barr virus blocks the autophagic flux and appropriates the autophagic machinery to enhance viral replication. J virology. (2014) 88:12715–26. doi: 10.1128/JVI.02199-14 PMC4248894 25142602

[11] Debnath J Gammoh N Ryan KM. Autophagy and autophagy-related pathways in cancer. Nat Rev Mol Cell Biol. (2023) 24:560–75. doi: 10.1038/s41580-023-00585-z PMC9980873 36864290

[12] Mulcahy Levy JM Thorburn A. Autophagy in cancer: moving from understanding mechanism to improving therapy responses in patients. Cell Death Differentiation. (2020) 27:843–57. doi: 10.1038/s41418-019-0474-7 PMC7206017 31836831

[13] Klionsky DJ Petroni G Amaravadi RK Baehrecke EH Ballabio A Boya P. Autophagy in major human diseases. EMBO J. (2021) 40:e108863. doi: 10.15252/embj.2021108863 34459017 PMC8488577

[14] Folkerts H Hilgendorf S Vellenga E Bremer E Wiersma VR. The multifaceted role of autophagy in cancer and the microenvironment. Medicinal Res Rev. (2019) 39:517–60. doi: 10.1002/med.2019.39.issue-2 PMC6585651 30302772

[15] Levy JMM Towers CG Thorburn A. Targeting autophagy in cancer. Nat Rev Cancer. (2017) 17:528–42. doi: 10.1038/nrc.2017.53 PMC5975367 28751651

[16] Chen Y-P Chan AT Le Q-T Blanchard P Sun Y Ma J. Nasopharyngeal carcinoma. Lancet. (2019) 394:64–80. doi: 10.1016/S0140-6736(19)30956-0 31178151

[17] Levine B Kroemer G. Biological functions of autophagy genes: a disease perspective. Cell. (2019) 176:11–42. doi: 10.1016/j.cell.2018.09.048 30633901 PMC6347410

[18] Santarelli R Granato M Faggioni A Cirone M. Interference with the autophagic process as a viral strategy to escape from the immune control: lesson from gamma herpesviruses. J Immunol Res. (2015) 2015:546063. doi: 10.1155/2015/546063 26090494 PMC4451563

[19] Li X He S Ma B. Autophagy and autophagy-related proteins in cancer. Mol cancer. (2020) 19:12. doi: 10.1186/s12943-020-1138-4 31969156 PMC6975070

[20] Thorburn A Thamm DH Gustafson DL. Autophagy and cancer therapy. Mol Pharmacol. (2014) 85:830–8. doi: 10.1124/mol.114.091850 PMC4014668 24574520

[21] Yiu SPT Hui KF Münz C Lo K-W Tsao SW Kao RYT. Autophagy-dependent reactivation of epstein-barr virus lytic cycle and combinatorial effects of autophagy-dependent and independent lytic inducers in nasopharyngeal carcinoma. Cancers. (2019) 11:1871. doi: 10.3390/cancers11121871 31769432 PMC6966612

[22] Alanazi AE Alhumaidy AA Almutairi H Awadalla ME Alkathiri A Alarjani M. Evolutionary analysis of LMP-1 genetic diversity in EBV-associated nasopharyngeal carcinoma: Bioinformatic insights into oncogenic potential. Infection Genet Evolution. (2024) 120:105586. doi: 10.1016/j.meegid.2024.105586 38508363

[23] Kim J Kundu M Viollet B Guan KL. AMPK and mTOR regulate autophagy through direct phosphorylation of Ulk1. Nat Cell Biol. (2011) 13:132–41. doi: 10.1038/ncb2152 PMC3987946 21258367

[24] Papinski D Kraft C. Regulation of autophagy by signaling through the Atg1/ULK1 complex. J Mol Biol. (2016) 428:1725–41. doi: 10.1016/j.jmb.2016.03.030 27059781

[25] Gil J Ramsey D Pawlowski P Szmida E Leszczynski P Bebenek M. The influence of tumor microenvironment on ATG4D gene expression in colorectal cancer patients. Med Oncol. (2018) 35:159. doi: 10.1007/s12032-018-1220-6 30374741 PMC6208841

[26] Poh SS Chua MLK Wee JT. Carcinogenesis of nasopharyngeal carcinoma: an alternate hypothetical mechanism. Chin J Cancer. (2016) 35:1–9. doi: 10.1007/s12032-018-1220-6 26738743 PMC4704291

[27] Liao L-J Hsu W-L Chen C-J Chiu Y-L. Feature reviews of the molecular mechanisms of nasopharyngeal carcinoma. Biomedicines. (2023) 11:1528. doi: 10.1186/s40880-015-0068-9 37371623 PMC10295754

[28] Rong Z Zheng K Chen J Jin X. Function and regulation of ULK1: From physiology to pathology. Gene. (2022) 840:146772. doi: 10.1016/j.gene.2022.146772 35905845

[29] Yun M Bai H-Y Zhang J-X Rong J Weng H-W Zheng Z-S. ULK1: a promising biomarker in predicting poor prognosis and therapeutic response in human nasopharygeal carcinoma. PloS One. (2015) 10:e0117375. doi: 10.1371/journal.pone.0117375 25714809 PMC4340914

[30] Shi D Zhang Y Mao T Liu D Liu W Luo B. MiR-BART2-3p targets Unc-51-like kinase 1 and inhibits cell autophagy and migration in Epstein-Barr virus-associated gastric cancer. Virus Res. (2021) 305:198567. doi: 10.1016/j.virusres.2021.198567 34555439

[31] Yun M. Bai H. Y. Zhang J. X. Rong J. Weng H. W. Zheng Z. S.. ULK1: a promising biomarker in predicting poor prognosis and therapeutic response in human nasopharygeal carcinoma. PloS one. (2015) 10(2):e0117375. doi: 10.1371/journal.pone.0117375 25714809 PMC4340914

[32] Shi D. Zhang Y. Mao T. Liu D. Liu W. Luo B. MiR-BART2-3p targets Unc-51-like kinase 1 and inhibits cell autophagy and migration in Epstein-Barr virus-associated gastric cancer. Virus research. (2021) 305:198567. doi: 10.1016/j.virusres.2021.198567 34555439

[33] Yang ZJ Chee CE Huang S Sinicrope FA. The role of autophagy in cancer: therapeutic implications. Mol Cancer Ther. (2011) 10(9):1533–41. doi: 10.1158/1535-7163.MCT-11-0047 PMC3170456 21878654

[34] Yun CW Lee SH. The roles of autophagy in cancer. Int J Mol Sci. (2018) 19(11):3466. doi: 10.3390/ijms19113466 30400561 PMC6274804

[35] Lin HC Chang Y Chen RY. Epstein-Barr virus latent membrane protein-1 upregulates autophagy and promotes viability in Hodgkin lymphoma: Implications for targeted therapy. Cancer Sci. (2021) 112(4):1589–602. doi: 10.1111/cas.14833 PMC8019199 33525055

[36] Bhattacharjee S Bose P Patel K. Transcriptional and epigenetic modulation of autophagy promotes EBV oncoprotein EBNA3C induced B-cell survival. Cell Death Dis. (2018) 9(6):605. doi: 10.1038/s41419-018-0668-9 29789559 PMC5964191

[37] Yiu SPT Dorothea M Hui KF Chiang AKS. Lytic induction therapy against Epstein–Barr virus-associated malignancies: Past, present, and future. Cancers. (2020) 12:2142. doi: 10.3390/cancers12082142 32748879 PMC7465660

[38] Hung CH Chen LW Wang WH. Regulation of autophagic activation by Rta of Epstein-Barr virus via the extracellular signal-regulated kinase pathway. J Virol. (2014) 88(20):12133–45. doi: 10.1128/JVI.02033-14 PMC4178756 25122800

