# Supplementary material for: Exploring the interplay between EBV and autophagy-related gene expression patterns in nasopharyngeal carcinoma
Source: Front Oncol. 2025 Jun 24;15:1588921. doi: 10.3389/fonc.2025.1588921 (PMC12234470; doi:10.3389/fonc.2025.1588921)
Supplement: Supplementary file 1 [file DataSheet1.docx]

**
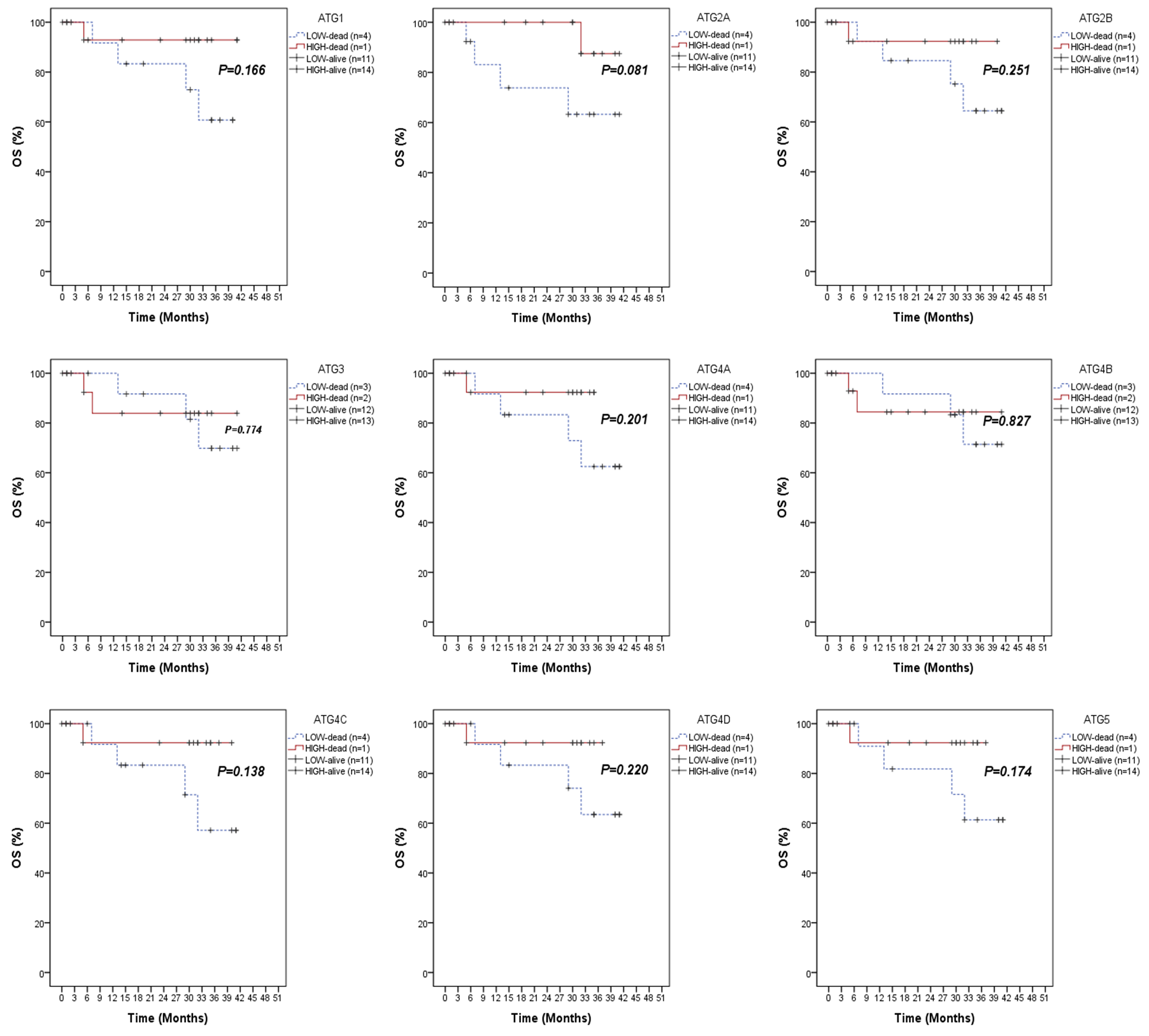
**

**Figure S1.** Overall survival (OS) of EBV+ NPC patients stratified by autophagy-related genes (ATG1, ATG2A, ATG2B, ATG3, ATG4A, ATG4B, ATG4C, ATG4D, and ATG5) expression status (low/high).

**
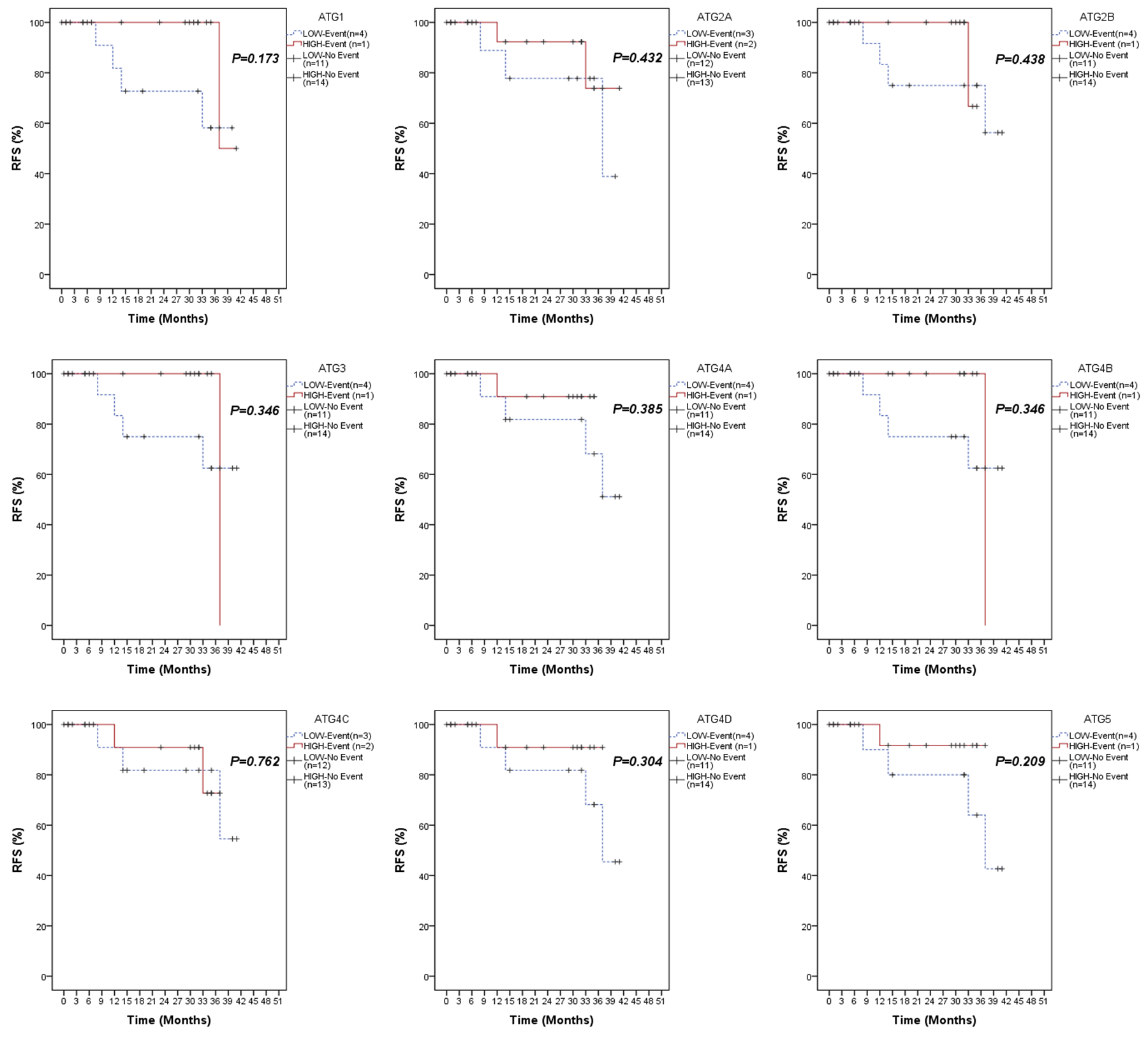
**

**Figure S2.** Recurrence-free survival (RFS) of EBV+ NPC patients stratified by autophagy-related genes (ATG1, ATG2A, ATG2B, ATG3, ATG4A, ATG4B, ATG4C, ATG4D, and ATG5) expression status (low/high).

**
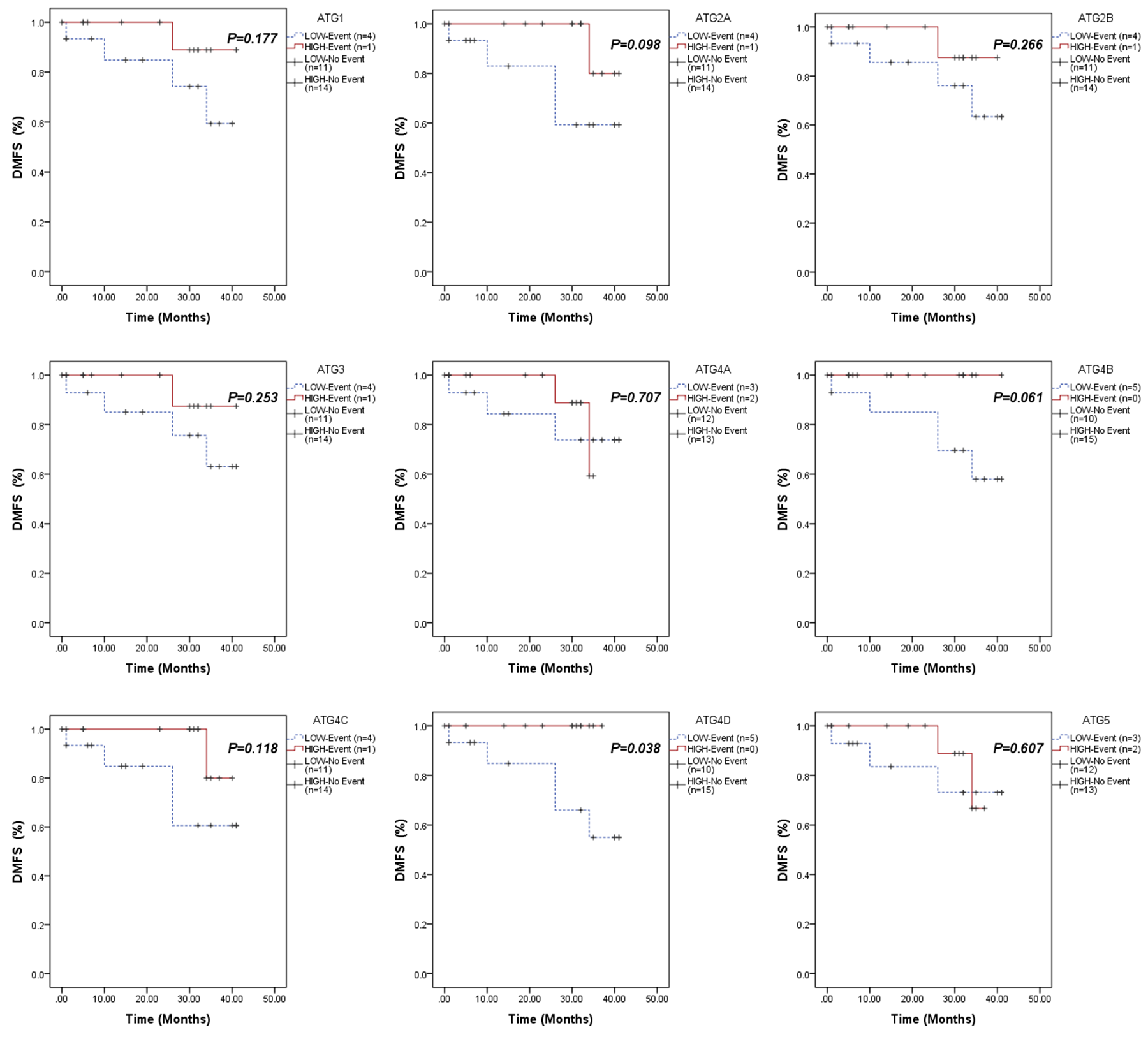
**

**Figure S3.** Distant metastasis-free survival (DMFS) of EBV+ NPC patients stratified by autophagy-related genes (ATG1, ATG2A, ATG2B, ATG3, ATG4A, ATG4B, ATG4C, ATG4D, and ATG5) expression status (low/high).

**
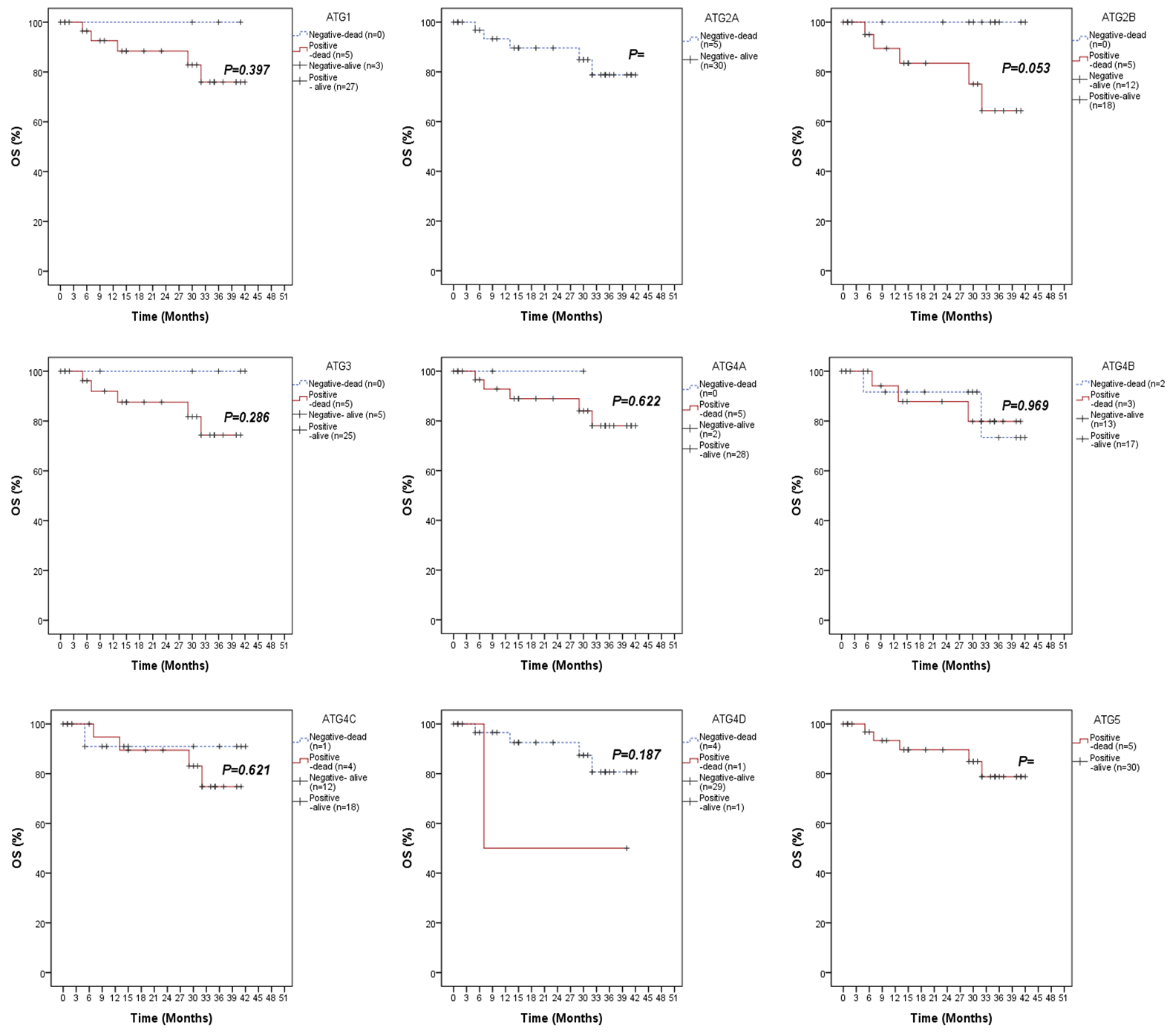
**

**Figure S4.** Overall survival (OS) of the entire series of NPC patients stratified by autophagy-related proteins (ATG1, ATG2A, ATG2B, ATG3, ATG4A, ATG4B, ATG4C, ATG4D, and ATG5) expression status (negative/positive).

**
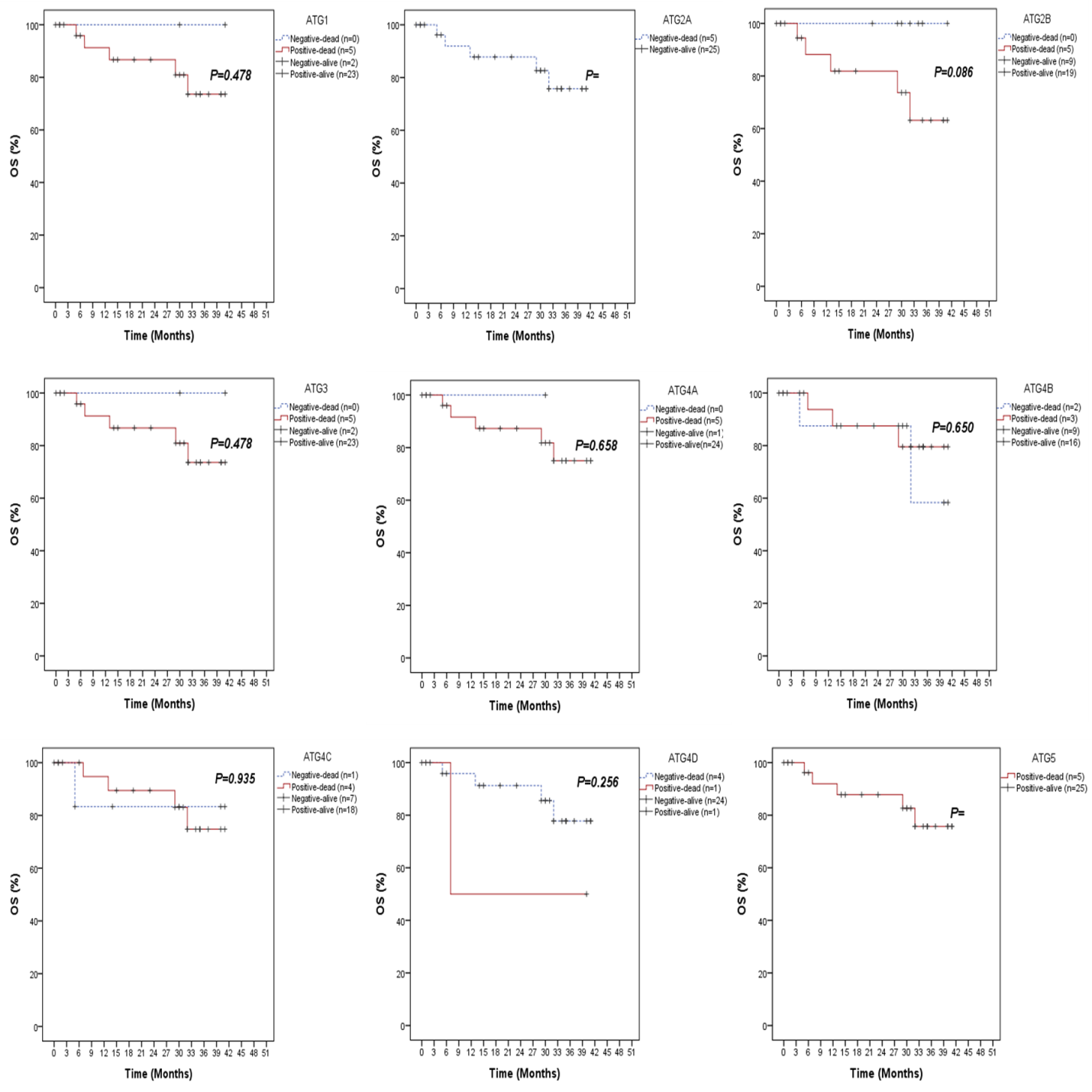
**

**Figure S5.** Overall survival (OS) of EBV+ NPC patients stratified by autophagy-related proteins (ATG1, ATG2A, ATG2B, ATG3, ATG4A, ATG4B, ATG4C, ATG4D, and ATG5) expression status (negative/positive).

**
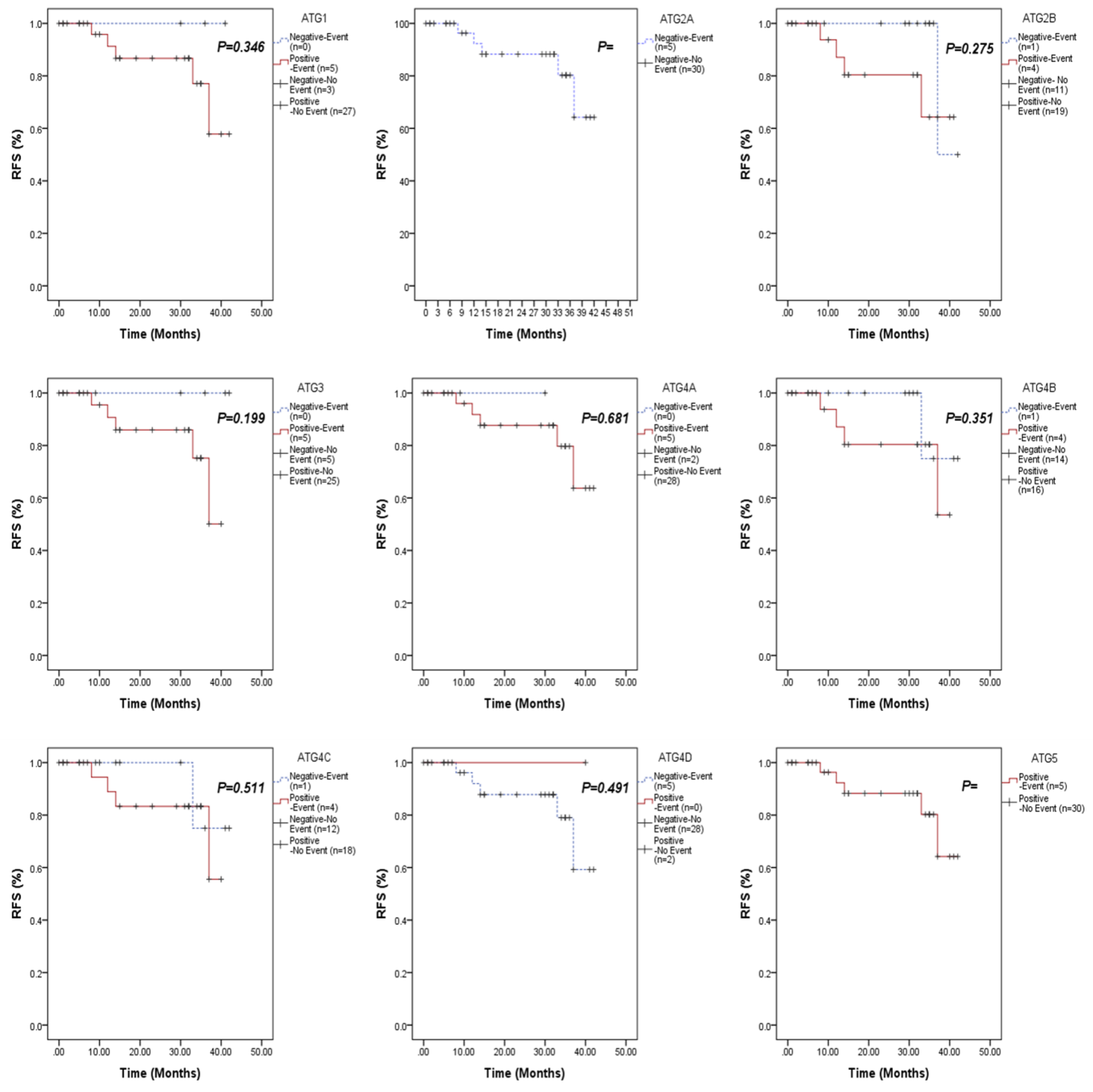
**

**Figure S6.** Recurrence-free survival (RFS) of the entire series of NPC patients stratified by autophagy-related proteins (ATG1, ATG2A, ATG2B, ATG3, ATG4A, ATG4B, ATG4C, ATG4D, and ATG5) expression status (negative/positive).

**
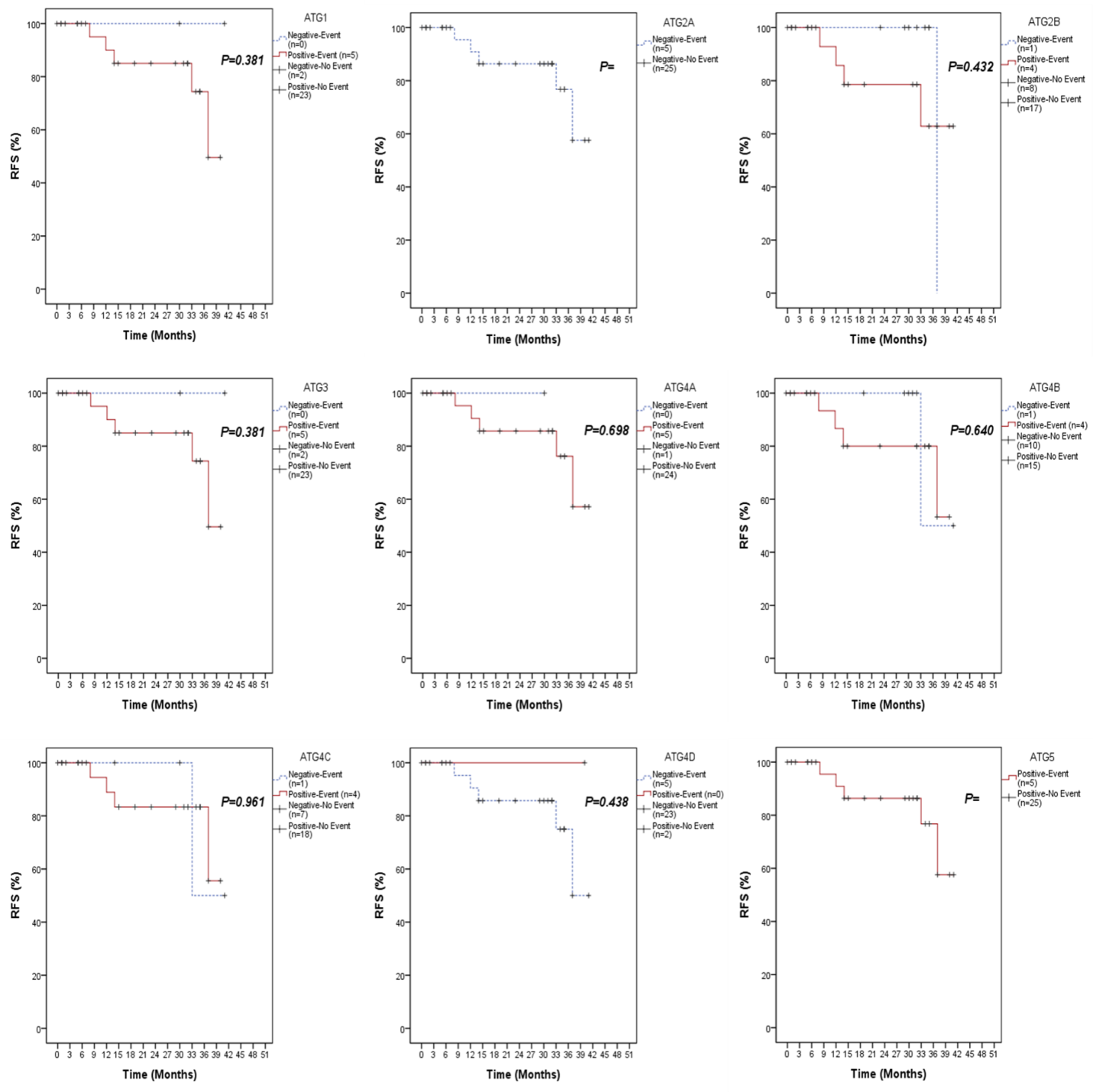
**

**Figure S7.** Recurrence-free survival (RFS) of EBV+ NPC patients stratified by autophagy-related proteins (ATG1, ATG2A, ATG2B, ATG3, ATG4A, ATG4B, ATG4C, ATG4D, and ATG5) expression status (negative/positive).

**
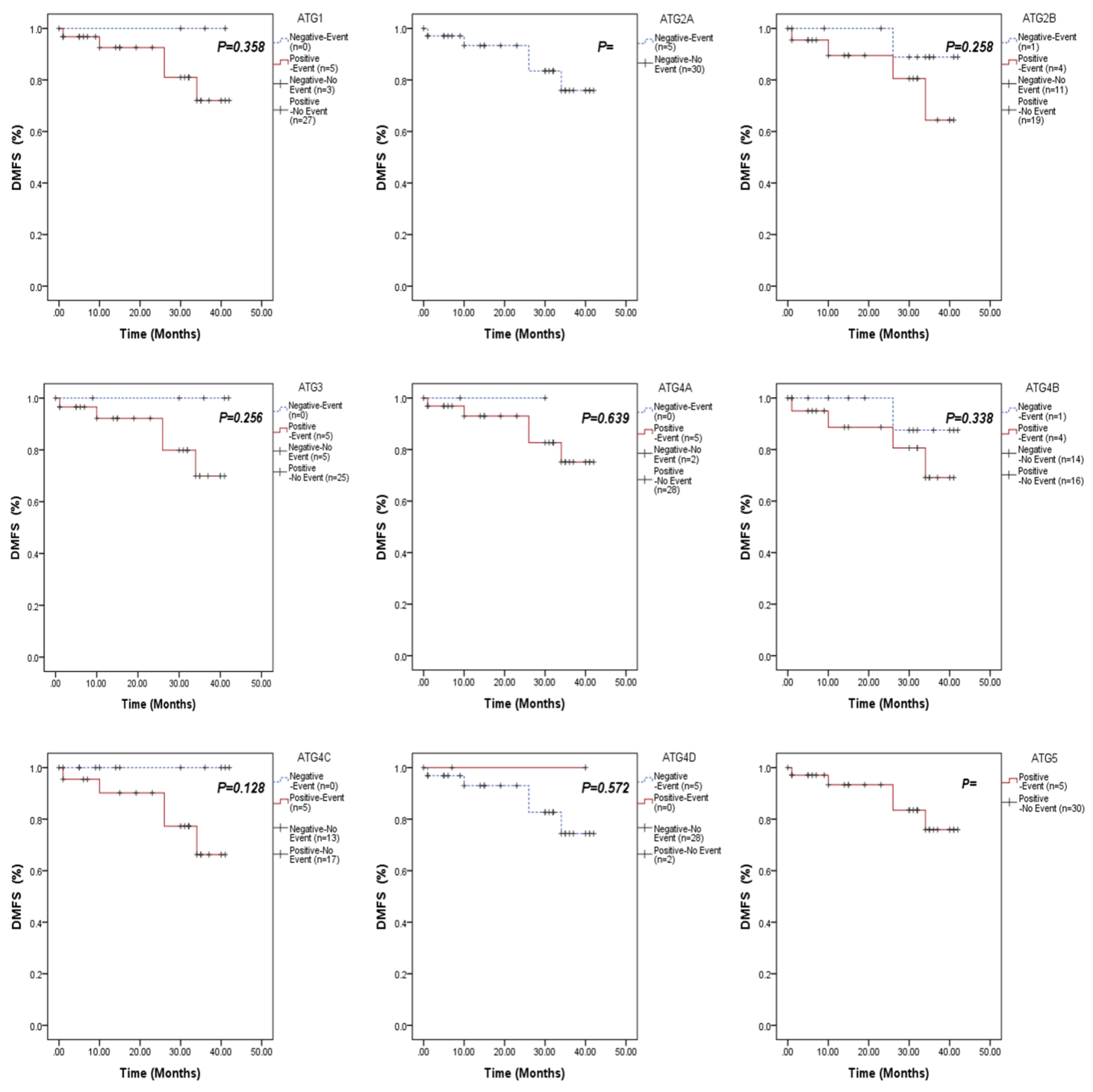
**

**Figure S8.** Distant metastasis-free survival (DMFS) of the entire series of NPC patients stratified by autophagy-related proteins (ATG1, ATG2A, ATG2B, ATG3, ATG4A, ATG4B, ATG4C, ATG4D, and ATG5) expression status (negative/positive).

**
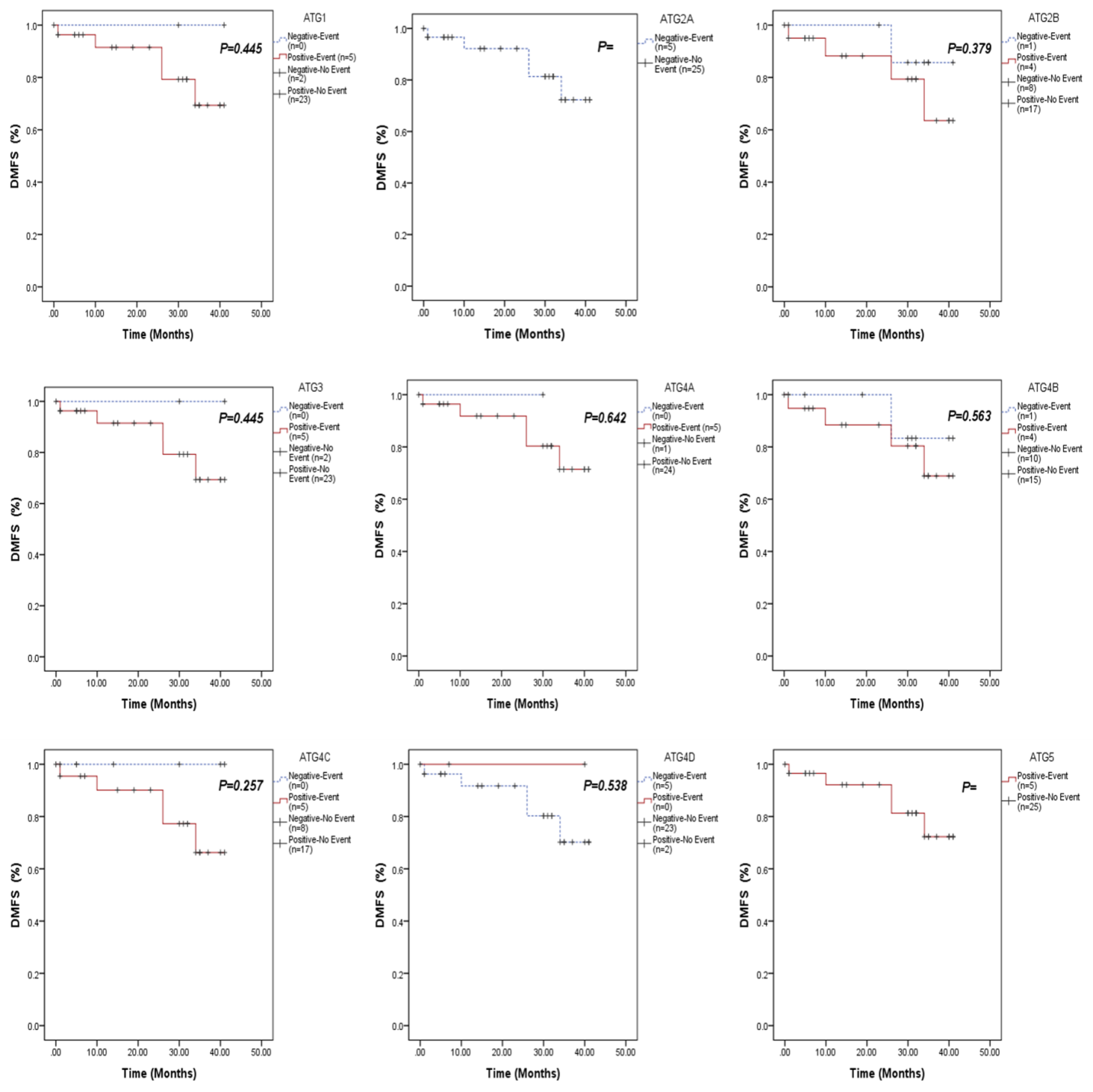
**

**Figure S9.** Distant metastasis-free survival (DMFS) of EBV+ NPC patients stratified by autophagy-related proteins (ATG1, ATG2A, ATG2B, ATG3, ATG4A, ATG4B, ATG4C, ATG4D, and ATG5) expression status (negative/positive).
