# Supplementary material for: Exploring the interplay between EBV and autophagy-related gene expression patterns in nasopharyngeal carcinoma
Source: Front Oncol. 2025 Jun 24;15:1588921. doi: 10.3389/fonc.2025.1588921 (PMC12234470; doi:10.3389/fonc.2025.1588921)
Supplement: Supplementary file 2 [file Table1.docx]

**Table S1.** Comparison of demographic and clinical characteristics with ATG1 gene expression in NPC patients

| **Characteristic** | **ATG1 gene expression** | | | | | | | | |
| --- | --- | --- | --- | --- | --- | --- | --- | --- | --- |
|  | **Entire series of NPC patients**  **(n=35)** | | | **NPC EBV−**  **(n=5)** | | | **NPC EBV+**  **(n=30)** | | |
|  | **Low†**  **(n=18)** | **High†**  **(n=17)** | ***P* value** | **Low†**  **(n= 3)** | **High†**  **(n=2)** | ***P* value** | **Low†**  **(n=15)** | **High†**  **(n=15)** | ***P* value** |
| **Sex**  Male, n (%)  Female, n (%) | 9(50)  9(50) | 10(58.82)  7(41.18) | 0.600 | 0(0)  3(100) | 2(100)  0(0) | 0.025* | 8(53.33)  7(46.67) | 9(60)  6(40) | 0.713 |
| **Age (years)** | 52.00**±**14.27 | 50.18**±**20.88 | 0.764 | 46.67±20.98 | 57±2.83 | 0.558 | 53.53±13.54 | 48.8±21.9 | 0.482 |
| **BMI (kg/m²)** | 27.00**±**5.38 | 26.61**±**6.36 | 0.846 | 25.31±7.06 | 27.55±0.24 | 0.699 | 27.51±5.28 | 26.31±6.72 | 0.589 |
| **EBV status**  EBV−, n (%)  EBV+, n (%) | 2(11.11)  16(88.89) | 3(17.65)  14(82.35) | 0.581 |  |  |  |  |  |  |
| **WHO histological classification**  Keratinizing SCC (K-NPC), n (%)  Non-keratinizing SCC (NK-NPC), n (%) | 0(0) 18(100) | 2(11.76)  15(88.24) | 0.134 | 1(33.33)  2(66.67) | 1(50)  1(50) | 0.709 | 0(0)  15(100) | 0(0)  15(100) | **-** |
| **Primary tumor (T) category**  T1, n (%)  T2, n (%)  T3, n (%)  T4, n (%) | 2(11.11)  1(5.56)  5(27.78)  10(55.56) | 1(5.88)  2(11.76)  10(58.82)  4(23.53) | 0.181 | 0(0)  0(0) 2(66.67)  1(33.33) | 0(0)  0(0) 1(50)  1(50) | 0.709 | 2(13.33)  1(6.67)  4(26.67)  8(53.33) | 1(6.67)  2(13.33)  8(53.33)  4(26.67) | 0.343 |
| **Regional lymph nodes (N) category**  N0, n (%)  N1, n (%)  N2, n (%)  N3, n (%) | 1(5.56)  5(27.78)  10(55.56)  2(11.11) | 3(17.65)  3(17.65)  9(52.94)  2(11.76) | 0.676 | 0(0)  1(33.33)  1(33.33)  1(33.33) | 1(50)  0(0)  1(50)  0(0) | 0.405 | 1(6.67)  4(26.67)  9(60)  1(6.67) | 2(13.33)  3(20)  8(53.33)  2(13.33) | 0.833 |
| **Distant metastasis (M1) category**  **No distant metastasis (M0) category**  M0, n (%)  M1, n (%) | 14(77.78)  4(22.22) | 16(94.12)  1(5.88) | 0.167 | 3(100)  0(0) | 2(100)  0(0) | **-** | 11(73.33)  4(26.67) | 14(93.33)  1(6.67) | 0.142 |
| **AJCC staging**  II, n (%)  III, n (%)  IVA, n (%)  IVB, n (%) | 0(0)  6(33.33)  8(44.44)  4(22.22) | 2(11.76)  9(52.94)  5(29.41)  1(5.88) | 0.167 | 0(0)  1(33.33)  2(66.67) 0(0) | 0(0)  1(50)  1(50) 0(0) | 0.709 | 0(0)  6(40)  5(33.33) 4(26.67) | 2(13.33)  7(46.67)  5(33.33) 1(6.67) | 0.275 |
| **SEER stage**  Localized, n (%)  Regional, n (%)  Distant, n (%) | 1(5.56)  13(72.22)  4(22.22) | 3(17.65)  13(76.47)  1(5.88) | 0.250 | 0(0)  3(100)  0(0) | 1(50)  1(50)  0(0) | 0.171 | 1(6.67)  10(66.67)  4(26.67) | 2(13.33)  12(80)  1(6.67) | 0.314 |
| **Recurrence**  Yes, n (%)  No, n (%) | 4(22.22) 14(77.78) | 1(5.88) 16(94.12) | 0.167 | 0(0)  3(100) | 0(0)  2(100) | **-** | 4(26.67)  11(73.33) | 1(6.67)  14(93.33) | 0.142 |
| **RFS (months)**  Yes, n (%)  No, n (%) | 14(77.78)  4(22.22) | 16(94.12)  1(5.88) | 0.167 | 3(100)  0(0) | 2(100)  0(0) | **-** | 11(73.33)  4(26.67) | 14(93.33)  1(6.67) | 0.142 |
| **DMFS (months)**  Yes, n (%)  No, n (%) | 14(77.78)  4(22.22) | 16(94.12)  1(5.88) | 0.167 | 3(100)  0(0) | 2(100)  0(0) | **-** | 11(73.33)  4(26.67) | 14(93.33)  1(6.67) | 0.142 |
| **OS (months)**  Alive, n (%)  Dead, n (%) | 14(77.78) 4(22.22) | 16(94.12)  1(5.88) | 0.167 | 3(100)  0(0) | 2(100)  0(0) | **-** | 11(73.33)  4(26.67) | 14(93.33)  1(6.67) | 0.142 |

Qualitative data are represented as the number of cases (%), whereas quantitative data are represented as mean ±SD (range, minimum-maximum) if normally distributed or as median (range or interquartile range, IQR: 25^th^ quartile to 75^th^ quartile) if non-normally distributed. * indicates a statistical significant difference. ^†^ The median expression value was used as a cut point to dichotomize patients into high and low expressers of autophagy-related genes.

**Table S2.** Comparison of demographic and clinical characteristics with ATG2A gene expression in NPC patients

| **Characteristic** | **ATG2A gene expression** | | | | | | | | |
| --- | --- | --- | --- | --- | --- | --- | --- | --- | --- |
|  | **Entire series of NPC patients**  **(n=35)** | | | **NPC EBV−**  **(n=5)** | | | **NPC EBV+**  **(n=30)** | | |
|  | **Low†**  **(n=18)** | **High†**  **(n=17)** | ***P* value** | **Low†**  **(n=3)** | **High†**  **(n=2)** | ***P* value** | **Low†**  **(n=15)** | **High†**  **(n=15)** | ***P* value** |
| **Sex**  Male, n (%)  Female, n (%) | 9(50) 9(50) | 10(58.82) 7(41.18) | 0.600 | 1(33.33)  2(66.67) | 1(50)  1(50) | 0.71 | 8(53.33)  7(46.67) | 9(60)  6(40) | 0.71 |
| **Age (years)** | 52.94±17.04 | 49.18±18.38 | 0.533 | 44±18.19 | 61±2.83 | 0.301 | 54.73±16.88 | 47.6±19.05 | 0.287 |
| **BMI (kg/m²)** | 25.24**±**6.05 | 28.46**±**5.17 | 0.101 | 24.34±6.17 | 29.01±1.82 | 0.393 | 25.43±6.23 | 28.39±5.5 | 0.178 |
| **EBV status**  EBV−, n (%)  EBV+, n (%) | 3(16.67)  15(83.33) | 2(11.76)  15(88.24) | 0.679 |  |  |  |  |  |  |
| **WHO histological classification**  Keratinizing SCC (K-NPC), n (%)  Non-keratinizing SCC (NK-NPC), n (%) | 0(0)  18(100) | 2(11.76)  15(88.24) | 0.134 | 0(0)  3(100) | 2(100)  0(0) | 0.03* | (0)  15(100) | (0)  15(100) | **-** |
| **Primary tumor (T) category**  T1, n (%)  T2, n (%)  T3, n (%)  T4, n (%) | 1(5.56)  2(11.11)  5(27.78)  10(55.56) | 2(11.76)  1(5.88)  10(58.82)  4(23.53) | 0.181 | 0(0) 0(0) 2(66.67)  1(33.33) | 0(0) 0(0) 1(50)  1(50) | 0.71 | 1(6.67)  2(13.33)  3(20)  9(60) | 2(13.33)  1(6.67)  9(60)  3(20) | 0.08 |
| **Regional lymph nodes (N) category**  N0, n (%)  N1, n (%)  N2, n (%)  N3, n (%) | 2(11.11)  3(16.67)  11(61.11)  2(11.11) | 2(11.76)  5(29.41)  8(47.06)  2(11.76) | 0.814 | 1(33.33)  1(33.33)  0(0)  1(33.33) | 0(0)  0(0)  2(100)  0(0) | 0.17 | 1(6.67)  2(13.33)  11(73.33)  1(6.67) | 2(13.33)  5(33.33)  6(40)  2(13.33) | 0.33 |
| **Distant metastasis (M1) category**  **No distant metastasis (M0) category**  M0, n (%)  M1, n (%) | 14(77.78)  4(22.22) | 16(94.12)  1(5.88) | 0.167 | 3(100)  (0) | 2(100)  (0) | **-** | 11(73.33)  4(26.67) | 14(93.33)  1(6.67) | 0.14 |
| **AJCC staging**  II, n (%)  III, n (%)  IVA, n (%)  IVB, n (%) | 1(5.56)  6(33.33)  7(38.89)  4(22.22) | 1(5.88)  9(52.94)  6(35.29)  1(5.88) | 0.484 | (0)  1(33.33)  2(66.67) (0) | (0)  1(50)  1(50) (0) | 0.71 | 1(6.67)  5(33.33)  5(33.33)  4(26.67) | 1(6.67)  8(53.33)  5(33.33)  1(6.67) | 0.48 |
| **SEER stage**  Localized, n (%)  Regional, n (%)  Distant, n (%) | 2(11.11)  12(66.67)  4(22.22) | 2(11.76)  14(82.35)  1(5.88) | 0.382 | 1(33.33)  2(66.67)  (0) | 0(0)  2(100)  (0) | 0.36 | 1(6.67)  10(66.67)  4(26.67) | 2(13.33)  12(80)  1(6.67) | 0.31 |
| **Recurrence**  Yes, n (%)  No, n (%) | 3(16.67)  15(83.33) | 2(11.76)  15(88.24) | 0.679 | (0)  3(100) | (0)  2(100) | **-** | 3(20)  12(80) | 2(13.33)  13(86.67) | 0.62 |
| **RFS (months)**  Yes, n (%)  No, n (%) | 15(83.33)  3(16.67) | 15(88.24)  2(11.76) | 0.679 | 3(100)  (0) | 2(100)  (0) | **-** | 12(80)  3(20) | 13(86.67)  2(13.33) | 0.62 |
| **DMFS (months)**  Yes, n (%)  No, n (%) | 14(77.78)  4(22.22) | 16(94.12)  1(5.88) | 0.167 | 3(100)  (0) | 2(100)  (0) | **-** | 11(73.33)  4(26.67) | 14(93.33)  1(6.67) | 0.14 |
| **OS (months)**  Alive, n (%)  Dead, n (%) | 14(77.78)  4(22.22) | 16(94.12)  1(5.88) | 0.167 | 3(100)  (0) | 2(100)  (0) | **-** | 11(73.33)  4(26.67) | 14(93.33)  1(6.67) | 0.14 |

Qualitative data are represented as the number of cases (%), whereas quantitative data are represented as mean ±SD (range, minimum-maximum) if normally distributed or as median (range or interquartile range, IQR: 25^th^ quartile to 75^th^ quartile) if non-normally distributed. * indicates a statistical significant difference. ^†^ The median expression value was used as a cut point to dichotomize patients into high and low expressers of autophagy-related genes.

**Table S3.** Comparison of demographic and clinical characteristics with ATG2B gene expression in NPC patients

| **Characteristic** | **ATG2B gene expression** | | | | | | | | |
| --- | --- | --- | --- | --- | --- | --- | --- | --- | --- |
|  | **Entire series of NPC patients (n=35)** | | | **NPC EBV−**  **(n=5)** | | | **NPC EBV+**  **(n=30)** | | |
|  | **Low†**  **(n=18 )** | **High†**  **(n= 17 )** | ***P* value** | **Low†**  **(n= 3 )** | **High†**  **(n= 2 )** | ***P* value** | **Low†**  **(n= 15 )** | **High†**  **(n=15 )** | ***P* value** |
| **Sex**  Male, n (%)  Female, n (%) | 9(50)  9(50) | 10(58.82)  7(41.18) | 0.600 | 0(0)  3(100) | 2(100)  0(0) | 0.025* | 8(53.33)  7(46.67) | 9(60)  6(40) | 0.713 |
| **Age (years)** | 51.67±17.44 | 50.53±18.18 | 0.851 | 46.67±20.98 | 57±2.83 | 0.558 | 51.47±15.77 | 50.87±20.65 | 0.929 |
| **BMI (kg/m²)** | 26.85±5.99 | 26.76±5.76 | 0.963 | 25.31±7.06 | 27.55±0.24 | 0.699 | 27.14±5.98 | 26.68±6.16 | 0.839 |
| **EBV status**  EBV−, n (%)  EBV+, n (%) | 2(11.11)  16(88.89) | 3(17.65)  14(82.35) | 0.581 |  |  |  |  |  |  |
| **WHO histological classification**  Keratinizing SCC (K-NPC), n (%)  Non-keratinizing SCC (NK-NPC), n (%) | 0(0)  18(100) | 2(11.76)  15(88.24) | 0.134 | 1(33.33)  2(66.67) | 1(50)  1(50) | 0.709 | 0(0)  15(100) | 0(0)  15(100) | **-** |
| **Primary tumor (T) category**  T1, n (%)  T2, n (%)  T3, n (%)  T4, n (%) | 2(11.11)  2(11.11)  5(27.78)  9(50) | 1(5.88)  1(5.88)  10(58.82)  5(29.41) | 0.327 | 0(0)  0(0)  2(66.67)  1(33.33) | 0(0)  0(0)  1(50)  1(50) | 0.709 | 2(13.33)  2(13.33)  3(20)  8(53.33) | 1(6.67)  1(6.67)  9(60)  4(26.67) | 0.172 |
| **Regional lymph nodes (N) category**  N0, n (%)  N1, n (%)  N2, n (%)  N3, n (%) | 1(5.56)  6(33.33)  9(50)  2(11.11) | 3(17.65)  2(11.76)  10(58.82)  2(11.76) | 0.388 | 0(0)  1(33.33)  1(33.33)  1(33.33) | 1(50)  0(0)  1(50)  0(0) | 0.405 | 1(6.67)  4(26.67)  9(60)  1(6.67) | 2(13.33)  3(20)  8(53.33)  2(13.33) | 0.833 |
| **Distant metastasis (M1) category**  **No distant metastasis (M0) category**  M0, n (%)  M1, n (%) | 14(77.78)  4(22.22) | 16(94.12)  1(5.88) | 0.167 | 3(100)  0(0) | 2(100)  0(0) | **-** | 11(73.33)  4(26.67) | 14(93.33)  1(6.67) | 0.142 |
| **AJCC staging**  II, n (%)  III, n (%)  IVA, n (%)  IVB, n (%) | 1(5.56)  6(33.33)  7(38.89)  4(22.22) | 1(5.88)  9(52.94)  6(35.29)  1(5.88) | 0.484 | 0(0)  1(33.33)  2(66.67)  0(0) | 0(0)  1(50)  1(50)  0(0) | 0.709 | 1(6.67)  5(33.33)  5(33.33)  4(26.67) | 1(6.67)  8(53.33)  5(33.33)  1(6.67) | 0.477 |
| **SEER stage**  Localized, n (%)  Regional, n (%)  Distant, n (%) | 1(5.56)  13(72.22)  4(22.22) | 3(17.65)  13(76.47)  1(5.88) | 0.250 | 0(0)  3(100)  0(0) | 1(50)  1(50)  0(0) | 0.171 | 1(6.67)  10(66.67)  4(26.67) | 2(13.33)  12(80)  1(6.67) | 0.314 |
| **Recurrence**  Yes, n (%)  No, n (%) | 4(22.22)  14(77.78) | 1(5.88)  16(94.12) | 0.167 | 0(0)  3(100) | 0(0)  2(100) | **-** | 4(26.67)  11(73.33) | 1(6.67)  14(93.33) | 0.142 |
| **RFS (months)**  Yes, n (%)  No, n (%) | 14(77.78)  4(22.22) | 16(94.12)  1(5.88) | 0.167 | 3(100)  0(0) | 2(100)  0(0) | **-** | 11(73.33)  4(26.67) | 14(93.33)  1(6.67) | 0.142 |
| **DMFS (months)**  Yes, n (%)  No, n (%) | 14(77.78)  4(22.22) | 16(94.12)  1(5.88) | 0.167 | 3(100)  0(0) | 2(100)  0(0) | **-** | 11(73.33)  4(26.67) | 14(93.33)  1(6.67) | 0.142 |
| **OS (months)**  Alive, n (%)  Dead, n (%) | 14(77.78)  4(22.22) | 16(94.12)  1(5.88) | 0.167 | 3(100)  0(0) | 2(100)  0(0) | **-** | 11(73.33)  4(26.67) | 14(93.33)  1(6.67) | 0.142 |

Qualitative data are represented as the number of cases (%), whereas quantitative data are represented as mean ±SD (range, minimum-maximum) if normally distributed or as median (range or interquartile range, IQR: 25^th^ quartile to 75^th^ quartile) if non-normally distributed. * indicates a statistical significant difference. ^†^ The median expression value was used as a cut point to dichotomize patients into high and low expressers of autophagy-related genes.

**Table S4.** Comparison of demographic and clinical characteristics with ATG3 gene expression in NPC patients

| **Characteristic** | **ATG3 gene expression** | | | | | | | | |
| --- | --- | --- | --- | --- | --- | --- | --- | --- | --- |
|  | **Entire series of NPC patients**  **(n=35)** | | | **NPC EBV−**  **(n=5)** | | | **NPC EBV+**  **(n=30)** | | |
|  | **Low†**  **(n=18)** | **High†**  **(n=17)** | ***P* value** | **Low†**  **(n=3)** | **High†**  **(n=2)** | ***P* value** | **Low†**  **(n=15)** | **High†**  **(n=15)** | ***P* value** |
| **Sex**  Male, n (%)  Female, n (%) | 9(50)  9(50) | 10(58.82)  7(41.18) | 0.600 | 1(33.33)  2(66.67) | 1(50)  1(50) | 0.709 | 9(60)  6(40) | 8(53.33)  7(46.67) | 0.713 |
| **Age (years)** | 54.39±14.37 | 47.65±20.25 | 0.262 | 58.67±4.51 | 39±22.63 | 0.431 | 53.6±15.62 | 48.73±20.46 | 0.470 |
| **BMI (kg/m²)** | 28.69±5.1 | 24.81±5.96 | 0.046* | 28.81±1.34 | 22.31±7.17 | 0.418 | 27.97±4.93 | 25.85±6.86 | 0.340 |
| **EBV status**  EBV−, n (%)  EBV+, n (%) | 2(11.11)  16(88.89) | 3(17.65)  14(82.35) | 0.581 |  |  |  |  |  |  |
| **WHO histological classification**  Keratinizing SCC (K-NPC), n (%)  Non-keratinizing SCC (NK-NPC), n (%) | 1(5.56)  17(94.44) | 1(5.88)  16(94.12) | 0.967 | 2(66.67)  1(33.33) | 0(0)  2(100) | 0.136 | 0(0)  15(100) | 0(0)  15(100) | **-** |
| **Primary tumor (T) category**  T1, n (%)  T2, n (%)  T3, n (%)  T4, n (%) | 2(11.11)  2(11.11)  6(33.33)  8(44.44) | 1(5.88)  1(5.88)  9(52.94)  6(35.29) | 0.676 | 0(0)  0(0)  2(66.67)  1(33.33) | 0(0)  0(0)  1(50)  1(50) | 0.709 | 2(13.33)  1(6.67)  4(26.67)  8(53.33) | 1(6.67)  2(13.33)  8(53.33)  4(26.67) | 0.343 |
| **Regional lymph nodes (N) category**  N0, n (%)  N1, n (%)  N2, n (%)  N3, n (%) | 2(11.11)  4(22.22)  10(55.56)  2(11.11) | 2(11.76)  4(23.53)  9(52.94)  2(11.76) | 0.999 | 0(0)  0(0)  2(66.67)  1(33.33) | 1(50)  1(50)  0(0)  0(0) | 0.172 | 2(13.33)  4(26.67)  8(53.33)  1(6.67) | 1(6.67)  3(20)  9(60)  2(13.33) | 0.833 |
| **Distant metastasis (M1) category**  **No distant metastasis (M0) category**  M0, n (%)  M1, n (%) | 14(77.78)  4(22.22) | 16(94.12)  1(5.88) | 0.167 | 3(100)  0(0) | 2(100)  0(0) | **-** | 11(73.33)  4(26.67) | 14(93.33)  1(6.67) | 0.142 |
| **AJCC staging**  II, n (%)  III, n (%)  IVA, n (%)  IVB, n (%) | 1(5.56)  7(38.89)  6(33.33)  4(22.22) | 1(5.88)  8(47.06)  7(41.18)  1(5.88) | 0.590 | 0(0)  1(33.33)  2(66.67)  0(0) | 0(0)  1(50)  1(50)  0(0) | 0.709 | 1(6.67)  5(33.33)  5(33.33)  4(26.67) | 1(6.67)  8(53.33)  5(33.33)  1(6.67) | 0.477 |
| **SEER stage**  Localized, n (%)  Regional, n (%)  Distant, n (%) | 2(11.11)  12(66.67)  4(22.22) | 2(11.76)  14(82.35)  1(5.88) | 0.382 | 0(0)  3(100)  0(0) | 1(50)  1(50)  0(0) | 0.171 | 2(13.33)  9(60)  4(26.67) | 1(6.67)  13(86.67)  1(6.67) | 0.239 |
| **Recurrence**  Yes, n (%)  No, n (%) | 4(22.22)  14(77.78) | 1(5.88)  16(94.12) | 0.167 | 0(0)  3(100) | 0(0)  2(100) | **-** | 4(26.67)  11(73.33) | 1(6.67)  14(93.33) | 0.142 |
| **RFS (months)**  Yes, n (%)  No, n (%) | 14(77.78)  4(22.22) | 16(94.12)  1(5.88) | 0.167 | 3(100)  0(0) | 2(100)  0(0) | **-** | 11(73.33)  4(26.67) | 14(93.33)  1(6.67) | 0.142 |
| **DMFS (months)**  Yes, n (%)  No, n (%) | 14(77.78)  4(22.22) | 16(94.12)  1(5.88) | 0.167 | 3(100)  0(0) | 2(100)  0(0) | **-** | 11(73.33)  4(26.67) | 14(93.33)  1(6.67) | 0.142 |
| **OS (months)**  Alive, n (%)  Dead, n (%) | 14(77.78)  4(22.22) | 16(94.12)  1(5.88) | 0.167 | 3(100)  0(0) | 2(100)  0(0) | **-** | 12(80)  3(20) | 13(86.67)  2(13.33) | 0.624 |

Qualitative data are represented as the number of cases (%), whereas quantitative data are represented as mean ±SD (range, minimum-maximum) if normally distributed or as median (range or interquartile range, IQR: 25^th^ quartile to 75^th^ quartile) if non-normally distributed. * indicates a statistical significant difference. ^†^ The median expression value was used as a cut point to dichotomize patients into high and low expressers of autophagy-related genes.

**Table S5.** Comparison of demographic and clinical characteristics with ATG4A gene expression in NPC patients

| **Characteristic** | **ATG4A gene expression** | | | | | | | | |
| --- | --- | --- | --- | --- | --- | --- | --- | --- | --- |
|  | **Entire series of NPC patients**  **(n=35)** | | | **NPC EBV−**  **(n=5)** | | | **NPC EBV+**  **(n=30)** | | |
|  | **Low†**  **(n=18)** | **High†**  **(n=17)** | ***P* value** | **Low†**  **(n= 3)** | **High†**  **(n=2)** | ***P* value** | **Low†**  **(n=15)** | **High†**  **(n= 15)** | ***P* value** |
| **Sex**  Male, n (%)  Female, n (%) | 9(50)  9(50) | 10(58.82)  7(41.18) | 0.600 | 0(0)  3(100) | 2(100)  0(0) | 0.025* | 8(53.33)  7(46.67) | 9(60)  6(40) | 0.713 |
| **Age (years)** | 54.56±17.48 | 47.47±17.38 | 0.238 | 46.67±20.98 | 57±2.83 | 0.558 | 53.93±19.11 | 48.4±17.12 | 0.411 |
| **BMI (kg/m²)** | 26.93±5.93 | 26.68±5.82 | 0.903 | 25.31±7.06 | 27.55±0.24 | 0.699 | 26.56±6.45 | 27.26±5.66 | 0.753 |
| **EBV status**  EBV−, n (%)  EBV+, n (%) | 2(11.11)  16(88.89) | 3(17.65)  14(82.35) | 0.581 |  |  |  |  |  |  |
| **WHO histological classification**  Keratinizing SCC (K-NPC), n (%)  Non-keratinizing SCC (NK-NPC), n (%) | 1(5.56)  17(94.44) | 1(5.88)  16(94.12) | 0.967 | 1(33.33)  2(66.67) | 1(50)  1(50) | 0.709 | 0(0)  15(100) | 0(0)  15(100) |  |
| **Primary tumor (T) category**  T1, n (%)  T2, n (%)  T3, n (%)  T4, n (%) | 1(5.56)  2(11.11)  9(50)  6(33.33) | 2(11.76)  1(5.88)  6(35.29)  8(47.06) | 0.676 | 0(0)  0(0)  2(66.67)  1(33.33) | 0(0)  0(0)  1(50)  1(50) | 0.709 | 1(6.67)  2(13.33)  7(46.67)  5(33.33) | 2(13.33)  1(6.67)  5(33.33)  7(46.67) | 0.721 |
| **Regional lymph nodes (N) category**  N0, n (%)  N1, n (%)  N2, n (%)  N3, n (%) | 2(11.11)  5(27.78)  9(50)  2(11.11) | 2(11.76)  3(17.65)  10(58.82)  2(11.76) | 0.913 | 0(0)  1(33.33)  1(33.33)  1(33.33) | 1(50)  0(0)  1(50)  0(0) | 0.405 | 2(13.33)  5(33.33)  7(46.67)  1(6.67) | 1(6.67)  2(13.33)  10(66.67)  2(13.33) | 0.479 |
| **Distant metastasis (M1) category**  **No distant metastasis (M0) category**  M0, n (%)  M1, n (%) | 15(83.33)  3(16.67) | 15(88.24)  2(11.76) | 0.679 | 3(100)  0(0) | 2(100)  0(0) | **-** | 12(80)  3(20) | 13(86.67)  2(13.33) | 0.624 |
| **AJCC staging**  II, n (%)  III, n (%)  IVA, n (%)  IVB, n (%) | 1(5.56)  10(55.56)  4(22.22)  3(16.67) | 1(5.88)  5(29.41)  9(52.94)  2(11.76) | 0.288 | 0(0)  1(33.33)  2(66.67)  0(0) | 0(0)  1(50)  1(50)  0(0) | 0.709 | 1(6.67)  9(60)  2(13.33)  3(20) | 1(6.67)  4(26.67)  8(53.33)  2(13.33) | 0.126 |
| **SEER stage**  Localized, n (%)  Regional, n (%)  Distant, n (%) | 2(11.11)  13(72.22)  3(16.67) | 2(11.76)  13(76.47)  2(11.76) | 0.918 | 0(0)  3(100)  0(0) | 1(50)  1(50)  0(0) | 0.171 | 2(13.33)  10(66.67)  3(20) | 1(6.67)  12(80)  2(13.33) | 0.699 |
| **Recurrence**  Yes, n (%)  No, n (%) | 4(22.22)  14(77.78) | 1(5.88)  16(94.12) | 0.167 | 0(0)  3(100) | 0(0)  2(100) | **-** | 4(26.67)  11(73.33) | 1(6.67)  14(93.33) | 0.142 |
| **RFS (months)**  Yes, n (%)  No, n (%) | 14(77.78)  4(22.22) | 16(94.12)  1(5.88) | 0.167 | 3(100)  0(0) | 2(100)  0(0) | **-** | 11(73.33)  4(26.67) | 14(93.33)  1(6.67) | 0.142 |
| **DMFS (months)**  Yes, n (%)  No, n (%) | 15(83.33)  3(16.67) | 15(88.24)  2(11.76) | 0.679 | 3(100)  0(0) | 2(100)  0(0) | **-** | 12(80)  3(20) | 13(86.67)  2(13.33) | 0.624 |
| **OS (months)**  Alive, n (%)  Dead, n (%) | 14(77.78)  4(22.22) | 16(94.12)  1(5.88) | 0.167 | 3(100)  0(0) | 2(100)  0(0) | **-** | 11(73.33)  4(26.67) | 14(93.33)  1(6.67) | 0.142 |

Qualitative data are represented as the number of cases (%), whereas quantitative data are represented as mean ±SD (range, minimum-maximum) if normally distributed or as median (range or interquartile range, IQR: 25^th^ quartile to 75^th^ quartile) if non-normally distributed. * indicates a statistical significant difference. ^†^ The median expression value was used as a cut point to dichotomize patients into high and low expressers of autophagy-related genes.

**Table S6.** Comparison of demographic and clinical characteristics with ATG4B gene expression in NPC patients

| **Characteristic** | **ATG4B gene expression** | | | | | | | | |
| --- | --- | --- | --- | --- | --- | --- | --- | --- | --- |
|  | **Entire series of NPC patients**  **(n=35)** | | | **NPC EBV−**  **(n=5)** | | | **NPC EBV+**  **(n=30)** | | |
|  | **Low†**  **(n=18)** | **High†**  **(n=17)** | ***P* value** | **Low†**  **(n=3)** | **High†**  **(n=2)** | ***P* value** | **Low†**  **(n=15)** | **High†**  **(n=15)** | ***P* value** |
| **Sex**  Male, n (%)  Female, n (%) | 9(50)  9(50) | 10(58.82)  7(41.18) | 0.600 | 0(0)  3(100) | 2(100)  0(0) | 0.025* | 7(46.67)  8(53.33) | 10(66.67)  5(33.33) | 0.269 |
| **Age (years)** | 50.17±16.11 | 52.12±19.4 | 0.748 | 46.67±20.98 | 57±2.83 | 0.558 | 52.27±15.92 | 50.07±20.47 | 0.745 |
| **BMI (kg/m²)** | 27.99±5.59 | 25.56±5.91 | 0.220 | 25.31±7.06 | 27.55±0.24 | 0.699 | 28.53±5.38 | 25.29±6.26 | 0.140 |
| **EBV status**  EBV−, n (%)  EBV+, n (%) | 1(5.56)  17(94.44) | 4(23.53)  13(76.47) | 0.129 |  |  |  |  |  |  |
| **WHO histological classification**  Keratinizing SCC (K-NPC), n (%)  Non-keratinizing SCC (NK-NPC), n (%) | 0(0)  18(100) | 2(11.76)  15(88.24) | 0.134 | 1(33.33)  2(66.67) | 1(50)  1(50) | 0.709 | 0(0)  15(100) | 0(0)  15(100) | **-** |
| **Primary tumor (T) category**  T1, n (%)  T2, n (%)  T3, n (%)  T4, n (%) | 2(11.11)  1(5.56)  6(33.33)  9(50) | 1(5.88)  2(11.76)  9(52.94)  5(29.41) | 0.497 | 0(0)  0(0)  2(66.67)  1(33.33) | 0(0)  0(0)  1(50)  1(50) | 0.709 | 2(13.33)  1(6.67)  5(33.33)  7(46.67) | 1(6.67)  2(13.33)  7(46.67)  5(33.33) | 0.721 |
| **Regional lymph nodes (N) category**  N0, n (%)  N1, n (%)  N2, n (%)  N3, n (%) | 2(11.11)  6(33.33)  8(44.44)  2(11.11) | 2(11.76)  2(11.76)  11(64.71)  2(11.76) | 0.485 | 0(0)  1(33.33)  1(33.33)  1(33.33) | 1(50)  0(0)  1(50)  0(0) | 0.405 | 2(13.33)  4(26.67)  7(46.67)  2(13.33) | 1(6.67)  3(20)  10(66.67)  1(6.67) | 0.720 |
| **Distant metastasis (M1) category**  **No distant metastasis (M0) category**  M0, n (%)  M1, n (%) | 13(72.22)  5(27.78) | 17(100)  0(0) | 0.019* | 3(100)  0(0) | 2(100)  0(0) | **-** | 10(66.67)  5(33.33) | 15(100)  0(0) | 0.014* |
| **AJCC staging**  II, n (%)  III, n (%)  IVA, n (%)  IVB, n (%) | 1(5.56)  6(33.33)  6(33.33)  5(27.78) | 1(5.88)  9(52.94)  7(41.18)  0(0) | 0.130 | 0(0)  1(33.33)  2(66.67)  0(0) | 0(0)  1(50)  1(50)  0(0) | 0.709 | 1(6.67)  5(33.33)  4(26.67)  5(33.33) | 1(6.67)  8(53.33)  6(40)  0(0) | 0.107 |
| **SEER stage**  Localized, n (%)  Regional, n (%)  Distant, n (%) | 2(11.11)  11(61.11)  5(27.78) | 2(11.76)  15(88.24)  0(0) | 0.061 | 0(0)  3(100)  0(0) | 1(50)  1(50)  0(0) | 0.171 | 2(13.33)  8(53.33)  5(33.33) | 1(6.67)  14(93.33)  0(0) | 0.031* |
| **Recurrence**  Yes, n (%)  No, n (%) | 4(22.22)  14(77.78) | 1(5.88)  16(94.12) | 0.167 | 0(0)  3(100) | 0(0)  2(100) | **-** | 4(26.67)  11(73.33) | 1(6.67)  14(93.33) | 0.142 |
| **RFS (months)**  Yes, n (%)  No, n (%) | 14(77.78)  4(22.22) | 16(94.12)  1(5.88) | 0.167 | 3(100)  0(0) | 2(100)  0(0) | **-** | 11(73.33)  4(26.67) | 14(93.33)  1(6.67) | 0.142 |
| **DMFS (months)**  Yes, n (%)  No, n (%) | 13(72.22)  5(27.78) | 17(100)  0(0) | 0.019* | 3(100)  0(0) | 2(100)  0(0) | **-** | 10(66.67)  5(33.33) | 15(100)  0(0) | 0.014* |
| **OS (months)**  Alive, n (%)  Dead, n (%) | 15(83.33)  3(16.67) | 15(88.24)  2(11.76) | 0.679 | 3(100)  0(0) | 2(100)  0(0) | **-** | 12(80)  3(20) | 13(86.67)  2(13.33) | 0.624 |

Qualitative data are represented as the number of cases (%), whereas quantitative data are represented as mean ±SD (range, minimum-maximum) if normally distributed or as median (range or interquartile range, IQR: 25^th^ quartile to 75^th^ quartile) if non-normally distributed. * indicates a statistical significant difference. ^†^ The median expression value was used as a cut point to dichotomize patients into high and low expressers of autophagy-related genes.

**Table S7.** Comparison of demographic and clinical characteristics with ATG4C gene expression in NPC patients

| **Characteristic** | **ATG4C gene expression** | | | | | | | | |
| --- | --- | --- | --- | --- | --- | --- | --- | --- | --- |
|  | **Entire series of NPC patients**  **(n=35)** | | | **NPC EBV−**  **(n=5)** | | | **NPC EBV+**  **(n=30)** | | |
|  | **Low†**  **(n=18)** | **High†**  **(n=17)** | ***P* value** | **Low†**  **(n=3)** | **High†**  **(n=2)** | ***P* value** | **Low†**  **(n=15)** | **High†**  **(n=15)** | ***P* value** |
| **Sex**  Male, n (%)  Female, n (%) | 9(50)  9(50) | 10(58.82)  7(41.18) | 0.600 | 0(0)  3(100) | 2(100)  0(0) | 0.025* | 9(60)  6(40) | 8(53.33)  7(46.67) | 0.713 |
| **Age (years)** | 56.11±16.1 | 45.82±17.91 | 0.083 | 46.67±20.98 | 57±2.83 | 0.558 | 54.33±16.54 | 48±19.49 | 0.346 |
| **BMI (kg/m²)** | 27.6±5.67 | 25.97±5.98 | 0.413 | 25.31±7.06 | 27.55±0.24 | 0.699 | 26.68±5.47 | 27.14±6.62 | 0.838 |
| **EBV status**  EBV−, n (%)  EBV+, n (%) | 2(11.11)  16(88.89) | 3(17.65)  14(82.35) | 0.581 |  |  |  |  |  |  |
| **WHO histological classification**  Keratinizing SCC (K-NPC), n (%)  Non-keratinizing SCC (NK-NPC), n (%) | 1(5.56)  17(94.44) | 1(5.88)  16(94.12) | 0.967 | 1(33.33)  2(66.67) | 1(50)  1(50) | 0.709 | 0(0)  15(100) | 0(0)  15(100) | **-** |
| **Primary tumor (T) category**  T1, n (%)  T2, n (%)  T3, n (%)  T4, n (%) | 1(5.56)  2(11.11)  7(38.89)  8(44.44) | 2(11.76)  1(5.88)  8(47.06)  6(35.29) | 0.803 | 0(0)  0(0)  2(66.67)  1(33.33) | 0(0)  0(0)  1(50)  1(50) | 0.709 | 1(6.67)  2(13.33)  4(26.67)  8(53.33) | 2(13.33)  1(6.67)  8(53.33)  4(26.67) | 0.343 |
| **Regional lymph nodes (N) category**  N0, n (%)  N1, n (%)  N2, n (%)  N3, n (%) | 2(11.11)  4(22.22)  10(55.56)  2(11.11) | 2(11.76)  4(23.53)  9(52.94)  2(11.76) | 0.999 | 0(0)  1(33.33)  1(33.33)  1(33.33) | 1(50)  0(0)  1(50)  0(0) | 0.405 | 1(6.67)  4(26.67)  9(60)  1(6.67) | 2(13.33)  3(20)  8(53.33)  2(13.33) | 0.833 |
| **Distant metastasis (M1) category**  **No distant metastasis (M0) category**  M0, n (%)  M1, n (%) | 14(77.78)  4(22.22) | 16(94.12)  1(5.88) | 0.167 | 3(100)  0(0) | 2(100)  0(0) | **-** | 11(73.33)  4(26.67) | 14(93.33)  1(6.67) | 0.142 |
| **AJCC staging**  II, n (%)  III, n (%)  IVA, n (%)  IVB, n (%) | 1(5.56)  8(44.44)  5(27.78)  4(22.22) | 1(5.88)  7(41.18)  8(47.06)  1(5.88) | 0.469 | 0(0)  1(33.33)  2(66.67)  0(0) | 0(0)  1(50)  1(50)  0(0) | 0.709 | 1(6.67)  6(40)  4(26.67)  4(26.67) | 1(6.67)  7(46.67)  6(40)  1(6.67) | 0.517 |
| **SEER stage**  Localized, n (%)  Regional, n (%)  Distant, n (%) | 2(11.11)  12(66.67)  4(22.22) | 2(11.76)  14(82.35)  1(5.88) | 0.382 | 0(0)  3(100)  0(0) | 1(50)  1(50)  0(0) | 0.171 | 1(6.67)  10(66.67)  4(26.67) | 2(13.33)  12(80)  1(6.67) | 0.314 |
| **Recurrence**  Yes, n (%)  No, n (%) | 3(16.67)  15(83.33) | 2(11.76)  15(88.24) | 0.679 | (0)  3(100) | (0)  2(100) | **-** | 3(20)  12(80) | 2(13.33)  13(86.67) | 0.62 |
| **RFS (months)**  Yes, n (%)  No, n (%) | 15(83.33)  3(16.67) | 15(88.24)  2(11.76) | 0.679 | 3(100)  (0) | 2(100)  (0) | **-** | 12(80)  3(20) | 13(86.67)  2(13.33) | 0.62 |
| **DMFS (months)**  Yes, n (%)  No, n (%) | 14(77.78)  4(22.22) | 16(94.12)  1(5.88) | 0.167 | 3(100)  0(0) | 2(100)  0(0) | - | 11(73.33)  4(26.67) | 14(93.33)  1(6.67) | 0.142 |
| **OS (months)**  Alive, n (%)  Dead, n (%) | 14(77.78)  4(22.22) | 16(94.12)  1(5.88) | 0.167 | 3(100)  0(0) | 2(100)  0(0) | **-** | 11(73.33)  4(26.67) | 14(93.33)  1(6.67) | 0.142 |

Qualitative data are represented as the number of cases (%), whereas quantitative data are represented as mean ±SD (range, minimum-maximum) if normally distributed or as median (range or interquartile range, IQR: 25^th^ quartile to 75^th^ quartile) if non-normally distributed. * indicates a statistical significant difference. ^†^ The median expression value was used as a cut point to dichotomize patients into high and low expressers of autophagy-related genes.

**Table S8.** Comparison of demographic and clinical characteristics with ATG4D gene expression in NPC patients

| **Characteristic** | **ATG4D gene expression** | | | | | | | | |
| --- | --- | --- | --- | --- | --- | --- | --- | --- | --- |
|  | **Entire series of NPC patients**  **(n=35)** | | | **NPC EBV−**  **(n=5)** | | | **NPC EBV+**  **(n=30)** | | |
|  | **Low†**  **(n=18)** | **High†**  **(n= 17)** | ***P* value** | **Low†**  **(n=3)** | **High†**  **(n=2)** | ***P* value** | **Low†**  **(n=15)** | **High†**  **(n=15)** | ***P* value** |
| **Sex**  Male, n (%)  Female, n (%) | 8(44.44)  10(55.56) | 11(64.71)  6(35.29) | 0.229 | 1(33.33)  2(66.67) | 1(50)  1(50) | 0.709 | 8(53.33)  7(46.67) | 9(60)  6(40) | 0.713 |
| **Age (years)** | 53.22±15.95 | 48.88±19.33 | 0.473 | 58.67±4.51 | 39±22.63 | 0.431 | 50.87±15.89 | 51.47±20.55 | 0.929 |
| **BMI (kg/m²)** | 27.46±6.17 | 26.11±5.47 | 0.499 | 28.81±1.34 | 22.31±7.17 | 0.418 | 26.51±6.06 | 27.3±6.06 | 0.724 |
| **EBV status**  EBV−, n (%)  EBV+, n (%) | 2(11.11)  16(88.89) | 3(17.65)  14(82.35) | 0.581 |  |  |  |  |  |  |
| **WHO histological classification**  Keratinizing SCC (K-NPC), n (%)  Non-keratinizing SCC (NK-NPC), n (%) | 1(5.56)  17(94.44) | 1(5.88)  16(94.12) | 0.967 | 2(66.67)  1(33.33) | 0(0)  2(100) | 0.136 | 0(0)  15(100) | 0(0)  15(100) | **-** |
| **Primary tumor (T) category**  T1, n (%)  T2, n (%)  T3, n (%)  T4, n (%) | 2(11.11)  2(11.11)  7(38.89)  7(38.89) | 1(5.88)  1(5.88)  8(47.06)  7(41.18) | 0.872 | 0(0)  0(0)  2(66.67)  1(33.33) | 0(0)  0(0)  1(50)  1(50) | 0.709 | 2(13.33)  2(13.33)  4(26.67)  7(46.67) | 1(6.67)  1(6.67)  8(53.33)  5(33.33) | 0.506 |
| **Regional lymph nodes (N) category**  N0, n (%)  N1, n (%)  N2, n (%)  N3, n (%) | 2(11.11)  3(16.67)  11(61.11)  2(11.11) | 2(11.76)  5(29.41)  8(47.06)  2(11.76) | 0.814 | 0(0)  0(0)  2(66.67)  1(33.33) | 1(50)  1(50)  0(0)  0(0) | 0.172 | 1(6.67)  3(20)  10(66.67)  1(6.67) | 2(13.33)  4(26.67)  7(46.67)  2(13.33) | 0.720 |
| **Distant metastasis (M1) category**  **No distant metastasis (M0) category**  M0, n (%)  M1, n (%) | 13(72.22)  5(27.78) | 17(100)  0(0) | 0.019* | 3(100)  0(0) | 2(100)  0(0) |  | 10(66.67)  5(33.33) | 15(100)  0(0) | 0.014* |
| **AJCC staging**  II, n (%)  III, n (%)  IVA, n (%)  IVB, n (%) | 1(5.56)  8(44.44)  4(22.22)  5(27.78) | 1(5.88)  7(41.18)  9(52.94)  0(0) | 0.073 | 0(0)  1(33.33)  2(66.67) 0(0) | 0(0)  1(50)  1(50) 0(0) | 0.709 | 1(6.67)  6(40)  3(20)  5(33.33) | 1(6.67)  7(46.67)  7(46.67)  0(0) | 0.083 |
| **SEER stage**  Localized, n (%)  Regional, n (%)  Distant, n (%) | 2(11.11)  11(61.11)  5(27.78) | 2(11.76)  15(88.24)  0(0) | 0.061 | 0(0)  3(100)  0(0) | 1(50)  1(50)  0(0) | 0.171 | 1(6.67)  9(60)  5(33.33) | 2(13.33)  13(86.67)  0(0) | 0.048* |
| **Recurrence**  Yes, n (%)  No, n (%) | 4(22.22)  14(77.78) | 1(5.88)  16(94.12) | 0.167 | 0(0)  3(100) | 0(0)  2(100) | **-** | 4(26.67)  11(73.33) | 1(6.67)  14(93.33) | 0.142 |
| **RFS (months)**  Yes, n (%)  No, n (%) | 14(77.78)  4(22.22) | 16(94.12)  1(5.88) | 0.167 | 3(100)  0(0) | 2(100)  0(0) | **-** | 11(73.33)  4(26.67) | 14(93.33)  1(6.67) | 0.142 |
| **DMFS (months)**  Yes, n (%)  No, n (%) | 13(72.22)  5(27.78) | 17(100)  0(0) | 0.019 | 3(100)  0(0) | 2(100)  0(0) | **-** | 10(66.67)  5(33.33) | 15(100)  0(0) | 0.014* |
| **OS (months)**  Alive, n (%)  Dead, n (%) | 14(77.78)  4(22.22) | 16(94.12)  1(5.88) | 0.167 | 3(100)  0(0) | 2(100)  0(0) | **-** | 11(73.33)  4(26.67) | 14(93.33)  1(6.67) | 0.142 |

Qualitative data are represented as the number of cases (%), whereas quantitative data are represented as mean ±SD (range, minimum-maximum) if normally distributed or as median (range or interquartile range, IQR: 25^th^ quartile to 75^th^ quartile) if non-normally distributed. * indicates a statistical significant difference. ^†^ The median expression value was used as a cut point to dichotomize patients into high and low expressers of autophagy-related genes.

**Table S9.** Comparison of demographic and clinical characteristics with ATG5 gene expression in NPC patients

| **Characteristic** | **ATG5 gene expression** | | | | | | | | |
| --- | --- | --- | --- | --- | --- | --- | --- | --- | --- |
|  | **Entire series of NPC patients (n=35)** | | | **NPC EBV−**  **(n=5)** | | | **NPC EBV+**  **(n=30)** | | |
|  | **Low†**  **(n=18)** | **High†**  **(n=17)** | ***P* value** | **Low†**  **(n=3)** | **High†**  **(n=2)** | ***P* value** | **Low†**  **(n=15)** | **High†**  **(n=15)** | ***P* value** |
| **Sex**  Male, n (%)  Female, n (%) | 9(50)  9(50) | 10(58.82)  7(41.18) | 0.600 | 0(0)  3(100) | 2(100)  0(0) | 0.025* | 9(60)  6(40) | 8(53.33)  7(46.67) | 0.713 |
| **Age (years)** | 50.5±18.8 | 51.76±16.67 | 0.835 | 46.67±20.98 | 57±2.83 | 0.558 | 49.27±20.37 | 53.07±15.89 | 0.573 |
| **BMI (kg/m²)** | 26.28±5.88 | 27.37±5.82 | 0.586 | 25.31±7.06 | 27.55±0.24 | 0.699 | 25.98±6.34 | 27.83±5.63 | 0.405 |
| **EBV status**  EBV−, n (%)  EBV+, n (%) | 2(11.11)  16(88.89) | 3(17.65)  14(82.35) | 0.581 |  |  |  |  |  |  |
| **WHO histological classification**  Keratinizing SCC (K-NPC), n (%)  Non-keratinizing SCC (NK-NPC), n (%) | 1(5.56)  17(94.44) | 1(5.88)  16(94.12) | 0.967 | 1(33.33)  2(66.67) | 1(50)  1(50) | 0.709 | 0(0)  15(100) | 0(0)  15(100) | **-** |
| **Primary tumor (T) category**  T1, n (%)  T2, n (%)  T3, n (%)  T4, n (%) | 2(11.11)  2(11.11)  9(50)  5(27.78) | 1(5.88)  1(5.88)  6(35.29)  9(52.94) | 0.497 | 0(0)  0(0)  2(66.67)  1(33.33) | 0(0)  0(0)  1(50)  1(50) | 0.709 | 1(6.67)  2(13.33)  7(46.67)  5(33.33) | 2(13.33)  1(6.67)  5(33.33)  7(46.67) | 0.721 |
| **Regional lymph nodes (N) category**  N0, n (%)  N1, n (%)  N2, n (%)  N3, n (%) | 2(11.11)  3(16.67)  10(55.56)  3(16.67) | 2(11.76)  5(29.41)  9(52.94)  1(5.88) | 0.676 | 0(0)  1(33.33)  1(33.33)  1(33.33) | 1(50)  0(0)  1(50)  0(0) | 0.405 | 2(13.33)  3(20)  9(60)  1(6.67) | 1(6.67)  4(26.67)  8(53.33)  2(13.33) | 0.833 |
| **Distant metastasis (M1) category**  **No distant metastasis (M0) category**  M0, n (%)  M1, n (%) | 15(83.33)  3(16.67) | 15(88.24)  2(11.76) | 0.679 | 3(100)  0(0) | 2(100)  0(0) | **-** | 12(80)  3(20) | 13(86.67)  2(13.33) | 0.624 |
| **AJCC staging**  II, n (%)  III, n (%)  IVA, n (%)  IVB, n (%) | 1(5.56)  10(55.56)  4(22.22)  3(16.67) | 1(5.88)  5(29.41)  9(52.94)  2(11.76) | 0.288 | 0(0)  1(33.33)  2(66.67)  0(0) | 0(0)  1(50)  1(50)  0(0) | 0.709 | 1(6.67)  9(60)  2(13.33)  3(20) | 1(6.67)  4(26.67)  8(53.33)  2(13.33) | 0.126 |
| **SEER stage**  Localized, n (%)  Regional, n (%)  Distant, n (%) | 2(11.11)  13(72.22)  3(16.67) | 2(11.76)  13(76.47)  2(11.76) | 0.918 | 0(0)  3(100)  0(0) | 1(50)  1(50)  0(0) | 0.171 | 2(13.33)  10(66.67)  3(20) | 1(6.67)  12(80)  2(13.33) | 0.699 |
| **Recurrence**  Yes, n (%)  No, n (%) | 4(22.22)  14(77.78) | 1(5.88)  16(94.12) | 0.167 | (0)  3(100) | (0)  2(100) | **-** | 4(26.67)  11(73.33) | 1(6.67)  14(93.33) | 0.142 |
| **RFS (months)**  Yes, n (%)  No, n (%) | 14(77.78)  4(22.22) | 16(94.12)  1(5.88) | 0.167 | 3(100)  (0) | 2(100)  (0) | **-** | 11(73.33)  4(26.67) | 14(93.33)  1(6.67) | 0.142 |
| **DMFS (months)**  Yes, n (%)  No, n (%) | 15(83.33)  3(16.67) | 15(88.24)  2(11.76) | 0.679 | 3(100)  0(0) | 2(100)  0(0) | - | 12(80) 3(20) | 13(86.67) 2(13.33) | 0.624 |
| **OS (months)**  Alive, n (%)  Dead, n (%) | 14(77.78)  4(22.22) | 16(94.12)  1(5.88) | 0.167 | 3(100)  0(0) | 2(100)  0(0) | **-** | 11(73.33)  4(26.67) | 14(93.33)  1(6.67) | 0.142 |

Qualitative data are represented as the number of cases (%), whereas quantitative data are represented as mean ±SD (range, minimum-maximum) if normally distributed or as median (range or interquartile range, IQR: 25^th^ quartile to 75^th^ quartile) if non-normally distributed. * indicates a statistical significant difference. ^†^ The median expression value was used as a cut point to dichotomize patients into high and low expressers of autophagy-related genes.

**Table S10.** Comparison of demographic and clinical characteristics with ATG1 protein immunoreactivity in NPC patients

| **Characteristic** | **ATG1 protein immunoreactivity** | | | | | | | | |
| --- | --- | --- | --- | --- | --- | --- | --- | --- | --- |
|  | **Entire series of NPC patients (n=35)** | | | **NPC EBV−**  **(n=5)** | | | **NPC EBV+**  **(n=30)** | | |
|  | **Negative†**  **(n=3)** | **Positive†**  **(n=32)** | ***P***  **value** | **Negative†**  **(n=1)** | **Positive†**  **(n=4)** | ***P* value** | **Negative†**  **(n=2)** | **Positive†**  **(n=28)** | ***P* value** |
| **Sex**  Male, n (%)  Female, n (%) | 2(66.67) 1(33.33) | 17(53.13) 15(46.88) | 0.653 | 1(100) 0(0) | 1(25) 3(75) | 0.171 | 1(50) 1(50) | 16(57.1) 12(42.9) | 0.844 |
| **Age (years)** | 43.67±9.87 | 51.81±18.04 | 0.450 | 55±0 | 49.75±18.21 | 0.813 | 38±1.41 | 52.11±18.34 | 0.294 |
| **BMI (kg/m²)** | 30.61±5.67 | 26.45±5.76 | 0.240 | 27.38±0 | 25.92±5.89 | 0.838 | 32.23±6.97 | 26.53±5.85 | 0.197 |
| **EBV status**  EBV−, n (%)  EBV+, n (%) | 1(33.33) 2(66.67) | 4(12.5) 28(87.5) | 0.324 |  |  |  |  |  |  |
| **WHO histological classification**  Keratinizing SCC (K-NPC), n (%)  Non-keratinizing SCC (NK-NPC), n (%) | 0(0) 3(100) | 2(6.25) 30(93.75) | 0.656 | 0(0) 1(100) | 2(50) 2(50) | 0.361 | 0(0) 2(100) | 0(0) 28(100) | **-** |
| **Primary tumor (T) category**  T1, n (%)  T2, n (%)  T3, n (%)  T4, n (%) | 0(0) 1(33.33) 2(66.67) 0(0) | 3(9.38) 2(6.25) 13(40.63) 14(43.75) | 0.224 | 0(0)  0(0) 1(100) 0(0) | 0(0)  0(0) 2(50) 2(50) | 0.361 | 0(0) 1(50) 1(50) 0(0) | 3(10.71) 2(7.14) 11(39.29) 12(42.86) | 0.361 |
| **Regional lymph nodes (N) category**  N0, n (%)  N1, n (%)  N2, n (%)  N3, n (%) | 1(33.33) 1(33.33) 0(0) 1(33.33) | 3(9.38) 7(21.88) 19(59.38) 3(9.38) | 0.196 | 1(100) 0(0) 0(0) 0(0) | 0(0) 1(25) 2(50) 1(25) | 0.172 | 0(0) 1(50) 0(0) 1(50) | 3(10.71) 6(21.43) 17(60.71) 2(7.14) | 0.138 |
| **Distant metastasis (M1) category**  **No distant metastasis (M0) category**  M0, n (%)  M1, n (%) | 3(100) 0(0) | 27(84.38) 5(15.63) | 0.460 | 1(100) 0(0) | 4(100) 0(0) | **-** | 2(100) 0(0) | 23(82.14) 5(17.86) | 0.513 |
| **AJCC staging**  II, n (%)  III, n (%)  IVA, n (%)  IVB, n (%) | 1(33.33) 1(33.33) 1(33.33) 0(0) | 1(3.13) 14(43.75) 12(37.5) 5(15.63) | 0.177 | 0(0) 1(100) 0(0) 0(0) | 0(0) 1(25) 3(75) 0(0) | 0.171 | 1(50) 0(0) 1(50) 0(0) | 1(3.57) 13(46.43) 9(32.14) 5(17.86) | 0.058 |
| **SEER stage**  Localized, n (%)  Regional, n (%)  Distant, n (%) | 1(33.33) 2(66.67) 0(0) | 3(9.38) 24(75) 5(15.63) | 0.392 | 1(100) 0(0)  0(0) | 0(0) 4(100) 0(0) | 0.025* | 0(0) 2(100) 0(0) | 3(10.71) 20(71.43) 5(17.86) | 0.677 |
| **Recurrence**  Yes, n (%)  No, n (%) | 0(0) 3(100) | 5(15.63) 27(84.38) | 0.460 | 0(0) 1(100) | 0(0) 4(100) | **-** | 0(0) 2(100) | 5(17.86) 23(82.14) | 0.513 |
| **RFS (months)**  Yes, n (%)  No, n (%) | 3(100) 0(0) | 27(84.38) 5(15.63) | 0.460 | 1(100) 0(0) | 4(100) 0(0) | **-** | 2(100) 0(0) | 23(82.14) 5(17.86) | 0.513 |
| **DMFS (months)**  Yes, n (%)  No, n (%) | 3(100) 0(0) | 27(84.38) 5(15.63) | 0.460 | 1(100) 0(0) | 4(100) 0(0) | **-** | 2(100) 0(0) | 23(82.14) 5(17.86) | 0.513 |
| **OS (months)**  Alive, n (%)  Dead, n (%) | 3(100) 0(0) | 27(84.38) 5(15.63) | 0.460 | 1(100) 0(0) | 4(100) 0(0) | **-** | 2(100) 0(0) | 23(82.14) 5(17.86) | 0.513 |

Immunoreactivity (immunostaining) of autophagy-related proteins was scored based on the staining intensity level as follows: 0 (no staining, negative), 1 (light brown staining, weak positive), 2 (medium brown staining, moderate positive), 3 (dark brown staining, strong positive). Data are represented as the number of cases (%). * indicates a statistical significant difference.

**Table S11.** Comparison of demographic and clinical characteristics with ATG2A protein immunoreactivity in NPC patients

| **Characteristic** | **ATG2A protein immunoreactivity** | | | | | | | | |
| --- | --- | --- | --- | --- | --- | --- | --- | --- | --- |
|  | **Entire series of NPC patients**  **(n=35)** | | | **NPC EBV−**  **(n=5)** | | | **NPC EBV+**  **(n=30)** | | |
|  | **Negative†**  **(n=35)** | **Positive†**  **(n=0)** | ***P* value** | **Negative†**  **(n=5)** | **Positive†**  **(n=0)** | ***P* value** | **Negative†**  **(n=30)** | **Positive†**  **(n=0)** | ***P* value** |
| **Sex**  Male, n (%)  Female, n (%) | 19(54.29) 16(45.71) |  |  | 2(40) 3(60) |  |  | 17(56.7) 13(43.3) |  |  |
| **Age (years)** | 51.11±17.55 |  |  | 50.8±15.94 |  |  | 51.17±18.05 |  |  |
| **BMI (kg/m²)** | 26.81±5.79 |  |  | 26.21±5.14 |  |  | 26.91±5.97 |  |  |
| **EBV status**  EBV−, n (%)  EBV+, n (%) | 5(14.29) 30(85.71) |  |  |  |  |  |  |  |  |
| **WHO histological classification**  Keratinizing SCC (K-NPC), n (%)  Non-keratinizing SCC (NK-NPC), n (%) | 2(5.71) 33(94.29) |  |  | 2(40) 3(60) |  |  | 0(0) 30(100) |  |  |
| **Primary tumor (T) category**  T1, n (%)  T2, n (%)  T3, n (%)  T4, n (%) | 3(8.57) 3(8.57) 15(42.86) 14(40) |  |  | 0(0)  0(0) 3(60) 2(40) |  |  | 3(10) 3(10) 12(40) 12(40) |  |  |
| **Regional lymph nodes (N) category**  N0, n (%)  N1, n (%)  N2, n (%)  N3, n (%) | 4(11.43) 8(22.86) 19(54.29) 4(11.43) |  |  | 1(20) 1(20) 2(40) 1(20) |  |  | 3(10) 7(23.33) 17(56.67) 3(10) |  |  |
| **Distant metastasis (M1) category**  **No distant metastasis (M0) category**  M0, n (%)  M1, n (%) | 30(85.71) 5(14.29) |  |  | 5(100) 0(0) |  |  | 25(83.33) 5(16.67) |  |  |
| **AJCC staging**  II, n (%)  III, n (%)  IVA, n (%)  IVB, n (%) | 2(5.71) 15(42.86) 13(37.14) 5(14.29) |  |  | 0(0) 2(40) 3(60) 0(0) |  |  | 2(6.67) 13(43.33) 10(33.33) 5(16.67) |  |  |
| **SEER stage**  Localized, n (%)  Regional, n (%)  Distant, n (%) | 4(11.43) 26(74.29) 5(14.29) |  |  | 1(20) 4(80)  0(0) |  |  | 3(10) 22(73.33) 5(16.67) |  |  |
| **Recurrence**  Yes, n (%)  No, n (%) | 5(14.29) 30(85.71) |  |  | 0(0) 5(100) |  |  | 5(16.67 25(83.33) |  |  |
| **RFS (months)**  Yes, n (%)  No, n (%) | 30(85.71) 5(14.29) |  |  | 5(100)  0(0) |  |  | 25(83.33) 5(16.67) |  |  |
| **DMFS (months)**  Yes, n (%)  No, n (%) | 30(85.71) 5(14.29) |  |  | 5(100) 0(0) |  |  | 25(83.33) 5(16.67) |  |  |
| **OS (months)**  Alive, n (%)  Dead, n (%) | 30(85.71) 5(14.29) |  |  | 5(100) 0(0) |  |  | 25(83.33) 5(16.67) |  |  |

Immunoreactivity (immunostaining) of autophagy-related proteins was scored based on the staining intensity level as follows: 0 (no staining, negative), 1 (light brown staining, weak positive), 2 (medium brown staining, moderate positive), 3 (dark brown staining, strong positive). Data are represented as the number of cases (%). * indicates a statistical significant difference.

**Table S12.** Comparison of demographic and clinical characteristics with ATG2B protein immunoreactivity in NPC patients

| **Characteristic** | **ATG2B protein immunoreactivity** | | | | | | | | |
| --- | --- | --- | --- | --- | --- | --- | --- | --- | --- |
|  | **Entire series of NPC patients**  **(n=35)** | | | **NPC EBV−**  **(n=5)** | | | **NPC EBV+**  **(n=30)** | | |
|  | **Negative†**  **(n=12)** | **Positive†**  **(n=23)** | ***P* value** | **Negative†**  **(n=3)** | **Positive†**  **(n=2)** | ***P* value** | **Negative†**  **(n= 9)** | **Positive†**  **(n=21)** | ***P* value** |
| **Sex**  Male, n (%)  Female, n (%) | 8(66.67) 4(33.33) | 11(47.83)  12(52.17) | 0.288 | 2(66.7) 1(33.3) | 0(0)  2(100) | 0.136 | 6(66.7) 3(33.3) | 11(52.4) 10(47.6) | 0.469 |
| **Age (years)** | 44±15.09 | 54.83±17.89 | 0.083 | 45.67±19.73 | 58.5±6.36 | 0.457 | 43.44±14.64 | 54.48±18.67 | 0.127 |
| **BMI (kg/m²)** | 25±5.99 | 27.75±5.58 | 0.185 | 24.11±5.95 | 29.35±1.34 | 0.328 | 25.29±6.34 | 27.6±5.83 | 0.340 |
| **EBV status**  EBV−, n (%)  EBV+, n (%) | 3(25) 9(75) | 2(8.7) 21(91.3) | 0.191 |  |  |  |  |  |  |
| **WHO histological classification**  Keratinizing SCC (K-NPC), n (%)  Non-keratinizing SCC (NK-NPC), n (%) | 1(8.33) 11(91.67) | 1(4.35) 22(95.65) | 0.630 | 1(33.33) 2(66.67) | 1(50) 1(50) | 0.709 | 0(0) 9(100) | 0(0) 21(100) | **-** |
| **Primary tumor (T) category**  T1, n (%)  T2, n (%)  T3, n (%)  T4, n (%) | 1(8.33) 1(8.33) 6(50) 4(33.33) | 2(8.7) 2(8.7) 9(39.13) 10(43.48) | 0.936 | 0(0)  0(0) 1(33.33) 2(66.67) | 0(0) 0(0) 2(100) 0(0) | 0.136 | 1(11.11) 1(11.11) 5(55.56) 2(22.22) | 2(9.52) 2(9.52) 7(33.33) 10(47.62) | 0.609 |
| **Regional lymph nodes (N) category**  N0, n (%)  N1, n (%)  N2, n (%)  N3, n (%) | 2(16.67) 3(25) 5(41.67) 2(16.67) | 2(8.7) 5(21.74) 14(60.87) 2(8.7) | 0.694 | 1(33.33) 1(33.33) 1(33.33) 0(0) | 0(0) 0(0) 1(50) 1(50) | 0.405 | 1(11.11) 2(22.22) 4(44.44) 2(22.22) | 2(9.52) 5(23.81) 13(61.9) 1(4.76) | 0.516 |
| **Distant metastasis (M1) category**  **No distant metastasis (M0) category**  M0, n (%)  M1, n (%) | 11(91.67) 1(8.33) | 19(82.61) 4(17.39) | 0.467 | 3(100)  0(0) | 2(100)  0(0) | **-** | 8(88.89) 1(11.11) | 17(80.95) 4(19.05) | 0.593 |
| **AJCC staging**  II, n (%)  III, n (%)  IVA, n (%)  IVB, n (%) | 1(8.33) 5(41.67) 5(41.67) 1(8.33) | 1(4.35) 10(43.48) 8(34.78) 4(17.39) | 0.855 | 0(0) 1(33.33) 2(66.67) 0(0) | 0(0) 1(50) 1(50) 0(0) | 0.709 | 1(11.11) 4(44.44) 3(33.33) 1(11.11) | 1(4.76) 9(42.86) 7(33.33) 4(19.05) | 0.891 |
| **SEER stage**  Localized, n (%)  Regional, n (%)  Distant, n (%) | 2(16.67) 9(75) 1(8.33) | 2(8.7) 17(73.91) 4(17.39) | 0.640 | 1(33.33) 2(66.67) 0(0) | 0(0)  2(100) 0(0) | 0.361 | 1(11.11) 7(77.78) 1(11.11) | 2(9.52) 15(71.43) 4(19.05) | 0.866 |
| **Recurrence**  Yes, n (%)  No, n (%) | 1(8.33) 11(91.67) | 4(17.39) 19(82.61) | 0.467 | 0(0) 3(100) | 0(0) 2(100) | **-** | 1(11.11) 8(88.89) | 4(19.05) 17(80.95) | 0.593 |
| **RFS (months)**  Yes, n (%)  No, n (%) | 11(91.67) 1(8.33) | 19(82.61) 4(17.39) | 0.467 | 3(100) 0(0) | 2(100) 0(0) | **-** | 8(88.89) 1(11.11) | 17(80.95) 4(19.05) | 0.593 |
| **DMFS (months)**  Yes, n (%)  No, n (%) | 11(91.67) 1(8.33) | 19(82.61) 4(17.39) | 0.467 | 3(100) 0(0) | 2(100) 0(0) | **-** | 8(88.89) 1(11.11) | 17(80.95) 4(19.05) | 0.593 |
| **OS (months)**  Alive, n (%)  Dead, n (%) | 12(100) 0(0) | 18(78.26) 5(21.74) | 0.081 | 3(100) 0(0) | 2(100) 0(0) | **-** | 9(100) 0(0) | 16(76.19) 5(23.81) | 0.109 |

Immunoreactivity (immunostaining) of autophagy-related proteins was scored based on the staining intensity level as follows: 0 (no staining, negative), 1 (light brown staining, weak positive), 2 (medium brown staining, moderate positive), 3 (dark brown staining, strong positive). Data are represented as the number of cases (%). * indicates a statistical significant difference.

**Table S13.** Comparison of demographic and clinical characteristics with ATG3 protein immunoreactivity in NPC patients

| **Characteristic** | **ATG3 protein immunoreactivity** | | | | | | | | |
| --- | --- | --- | --- | --- | --- | --- | --- | --- | --- |
|  | **Entire series of NPC patients**  **(n=35)** | | | **NPC EBV−**  **(n=5)** | | | **NPC EBV+**  **(n=30)** | | |
|  | **Negative†**  **(n=5)** | **Positive†**  **(n=30)** | ***P***  **value** | **Negative†**  **(n=3)** | **Positive†**  **(n=2)** | ***P***  **value** | **Negative†**  **(n=2)** | **Positive†**  **(n=28)** | ***P***  **value** |
| **Sex**  Male, n (%)  Female, n (%) | 3(60) 2(40) | 16(53.33) 14(46.67) | 0.782 | 2(66.7) 1(33.3) | 0(0) 2(100) | 0.136 | 1(50) 1(50) | 16(57.1) 12(42.9) | 0.844 |
| **Age (years)** | 42.6±14.59 | 52.53±17.8 | 0.247 | 45.67±19.73 | 58.5±6.36 | 0.457 | 38±1.41 | 52.11±18.34 | 0.294 |
| **BMI (kg/m²)** | 27.36±7.05 | 26.72±5.7 | 0.822 | 24.11±5.95 | 29.35±1.34 | 0.328 | 32.23±6.97 | 26.53±5.85 | 0.197 |
| **EBV status**  EBV−, n (%)  EBV+, n (%) | 3(60) 2(40) | 2(6.67) 28(93.33) | 0.002* |  |  |  |  |  |  |
| **WHO histological classification**  Keratinizing SCC (K-NPC), n (%)  Non-keratinizing SCC (NK-NPC), n (%) | 1(20) 4(80) | 1(3.33) 29(96.67) | 0.137 | 1(33.33) 2(66.67) | 1(50) 1(50) | 0.709 | 0(0) 2(100) | 0(0) 28(100) | **-** |
| **Primary tumor (T) category**  T1, n (%)  T2, n (%)  T3, n (%)  T4, n (%) | 0(0) 1(20) 2(40) 2(40) | 3(10) 2(6.67) 13(43.33) 12(40) | 0.706 | 0(0)  0(0) 1(33.33) 2(66.67) | 0(0)  0(0) 2(100) 0(0) | 0.136 | 0(0) 1(50) 1(50) 0(0) | 3(10.71) 2(7.14) 11(39.29) 12(42.86) | 0.208 |
| **Regional lymph nodes (N) category**  N0, n (%)  N1, n (%)  N2, n (%)  N3, n (%) | 1(20) 2(40) 1(20) 1(20) | 3(10) 6(20) 18(60) 3(10) | 0.430 | 1(33.33) 1(33.33) 1(33.33) 0(0) | 0(0) 0(0) 1(50) 1(50) | 0.405 | 0(0) 1(50) 0(0) 1(50) | 3(10.71) 6(21.43) 17(60.71) 2(7.14) | 0.138 |
| **Distant metastasis (M1) category**  **No distant metastasis (M0) category**  M0, n (%)  M1, n (%) | 5(100) 0(0) | 25(83.33) 5(16.67) | 0.324 | 3(100) 0(0) | 2(100) 0(0) | **-** | 2(100) 0(0) | 23(82.14) 5(17.86) | 0.513 |
| **AJCC staging**  II, n (%)  III, n (%)  IVA, n (%)  IVB, n (%) | 1(20) 1(20) 3(60) 0(0) | 1(3.33)  14(46.67) 10(33.33) 5(16.67) | 0.217 | 0(0) 1(33.33) 2(66.67)  0(0) | 0(0) 1(50) 1(50) 0(0) | 0.709 | 1(50) 0(0) 1(50) 0(0) | 1(3.57) 13(46.43) 9(32.14) 5(17.86) | 0.058 |
| **SEER stage**  Localized, n (%)  Regional, n (%)  Distant, n (%) | 1(20) 4(80) 0(0) | 3(10) 22(73.33) 5(16.67) | 0.540 | 1(33.33) 2(66.67) 0(0) | 0(0) 2(100) 0(0) | 0.361 | 0(0) 2(100) 0(0) | 3(10.71) 20(71.43) 5(17.86) | 0.677 |
| **Recurrence**  Yes, n (%)  No, n (%) | 0(0) 5(100) | 5(16.67) 25(83.33) | 0.324 | 0(0) 3(100) | 0(0) 2(100) | **-** | 0(0) 2(100) | 5(17.86) 23(82.14) | 0.513 |
| **RFS (months)**  Yes, n (%)  No, n (%) | 5(100) 0(0) | 25(83.33) 5(16.67) | 0.324 | 3(100) 0(0) | 2(100) 0(0) | **-** | 2(100) 0(0) | 23(82.14) 5(17.86) | 0.513 |
| **DMFS (months)**  Yes, n (%)  No, n (%) | 5(100) 0(0) | 25(83.33) 5(16.67) | 0.324 | 3(100) 0(0) | 2(100) 0(0) | **-** | 2(100) 0(0) | 23(82.14) 5(17.86) | 0.513 |
| **OS (months)**  Alive, n (%)  Dead, n (%) | 5(100) 0(0) | 25(83.33) 5(16.67) | 0.324 | 3(100) 0(0) | 2(100) 0(0) | **-** | 2(100) 0(0) | 23(82.14) 5(17.86) | 0.513 |

Immunoreactivity (immunostaining) of autophagy-related proteins was scored based on the staining intensity level as follows: 0 (no staining, negative), 1 (light brown staining, weak positive), 2 (medium brown staining, moderate positive), 3 (dark brown staining, strong positive). Data are represented as the number of cases (%). * indicates a statistical significant difference.

**Table S14.** Comparison of demographic and clinical characteristics with ATG4A protein immunoreactivity in NPC patients

| **Characteristic** | **ATG4A protein immunoreactivity** | | | | | | | | |
| --- | --- | --- | --- | --- | --- | --- | --- | --- | --- |
|  | **Entire series of NPC patients**  **(n=35)** | | | **NPC EBV−**  **(n=5)** | | | **NPC EBV+**  **(n=30)** | | |
|  | **Negative†**  **(n=2)** | **Positive†**  **(n=33)** | ***P***  **value** | **Negative†**  **(n=1)** | **Positive†**  **(n=4)** | ***P***  **value** | **Negative†**  **(n=1)** | **Positive†**  **(n=29)** | ***P***  **value** |
| **Sex**  Male, n (%)  Female, n (%) | 2(100) 0(0) | 17(51.52) 16(48.48) | 0.181 | 1(100) 0(0) | 1(25) 3(75) | 0.171 | 1(100) 0(0) | 16(55.2) 13(44.8) | 0.374 |
| **Age (years)** | 49±14.14 | 51.24±17.9 | 0.864 | 59±0 | 48.75±17.63 | 0.639 | 39±0 | 51.59±18.22 | 0.503 |
| **BMI (kg/m²)** | 32.44±6.68 | 26.47±5.67 | 0.160 | 27.72±0 | 25.83±5.85 | 0.792 | 37.16±0 | 26.56±5.75 | 0.080 |
| **EBV status**  EBV−, n (%)  EBV+, n (%) | 1(50) 1(50) | 4(12.12) 29(87.88) | 0.137 |  |  |  |  |  |  |
| **WHO histological classification**  Keratinizing SCC (K-NPC), n (%)  Non-keratinizing SCC (NK-NPC), n (%) | 1(50) 1(50) | 1(3.03) 32(96.97) | 0.005* | 1(100) 0(0) | 1(25) 3(75) | 0.171 | 0(0) 1(100) | 0(0) 29(100) | **-** |
| **Primary tumor (T) category**  T1, n (%)  T2, n (%)  T3, n (%)  T4, n (%) | 0(0) 0(0) 1(50) 1(50) | 3(9.09) 3(9.09) 14(42.42) 13(39.39) | 0.931 | 0(0)  0(0) 0(0) 1(100) | 0(0)  0(0) 3(75) 1(25) | 0.171 | 0(0) 0(0) 1(100) 0(0) | 3(10.34) 3(10.34) 11(37.93) 12(41.38) | 0.670 |
| **Regional lymph nodes (N) category**  N0, n (%)  N1, n (%)  N2, n (%)  N3, n (%) | 0(0) 0(0) 1(50) 1(50) | 4(12.12) 8(24.24) 18(54.55) 3(9.09) | 0.321 | 0(0) 0(0) 1(100) 0(0) | 1(25) 1(25) 1(25) 1(25) | 0.599 | 0(0) 0(0) 0(0) 1(100) | 3(10.34) 7(24.14) 17(58.62) 2(6.9) | 0.025* |
| **Distant metastasis (M1) category**  **No distant metastasis (M0) category**  M0, n (%)  M1, n (%) | 2(100) 0(0) | 28(84.85) 5(15.15) | 0.552 | 1(100) 0(0) | 4(100) 0(0) | **-** | 1(100) 0(0) | 24(82.76) 5(17.24) | 0.649 |
| **AJCC staging**  II, n (%)  III, n (%)  IVA, n (%)  IVB, n (%) | 0(0) 0(0) 2(100) 0(0) | 2(6.06) 15(45.45) 11(33.33) 5(15.15) | 0.309 | 0(0) 0(0)  1(100) 0(0) | 0(0) 2(50) 2(50) 0(0) | 0.361 | 0(0) 0(0) 1(100) 0(0) | 2(6.9) 13(44.83) 9(31.03) 5(17.24) | 0.558 |
| **SEER stage**  Localized, n (%)  Regional, n (%)  Distant, n (%) | 0(0) 2(100) 0(0) | 4(12.12) 24(72.73) 5(15.15) | 0.693 | 0(0) 1(100) 0(0) | 1(25) 3(75) 0(0) | 0.576 | 0(0) 1(100) 0(0) | 3(10.34) 21(72.41) 5(17.24) | 0.829 |
| **Recurrence**  Yes, n (%)  No, n (%) | 0(0) 2(100) | 5(15.15) 28(84.85) | 0.552 | 0(0) 1(100) | 0(0) 4(100) | **-** | 0(0) 1(100) | 5(17.24) 24(82.76) | 0.649 |
| **RFS (months)**  Yes, n (%)  No, n (%) | 2(100) 0(0) | 28(84.85) 5(15.15) | 0.552 | 1(100) 0(0) | 4(100) 0(0) | **-** | 1(100) 0(0) | 24(82.76) 5(17.24) | 0.649 |
| **DMFS (months)**  Yes, n (%)  No, n (%) | 2(100) 0(0) | 28(84.85) 5(15.15) | 0.552 | 1(100) 0(0) | 4(100) 0(0) | **-** | 1(100) 0(0) | 24(82.76) 5(17.24) | 0.649 |
| **OS (months)**  Alive, n (%)  Dead, n (%) | 2(100) 0(0) | 28(84.85) 5(15.15) | 0.552 | 1(100) 0(0) | 4(100) 0(0) | **-** | 1(100) 0(0) | 24(82.76) 5(17.24) | 0.649 |

Immunoreactivity (immunostaining) of autophagy-related proteins was scored based on the staining intensity level as follows: 0 (no staining, negative), 1 (light brown staining, weak positive), 2 (medium brown staining, moderate positive), 3 (dark brown staining, strong positive). Data are represented as the number of cases (%). * indicates a statistical significant difference.

**Table S15.** Comparison of demographic and clinical characteristics with ATG4B protein immunoreactivity in NPC patients

| **Characteristic** | **ATG4B protein immunoreactivity** | | | | | | | | |
| --- | --- | --- | --- | --- | --- | --- | --- | --- | --- |
|  | **Entire series of NPC patients**  **(n=35)** | | | **NPC EBV−**  **(n=5)** | | | **NPC EBV+**  **(n=30)** | | |
|  | **Negative†**  **(n=15)** | **Positive†**  **(n=20)** | ***P***  **value** | **Negative†**  **(n=4)** | **Positive†**  **(n=1)** | ***P***  **value** | **Negative†**  **(n=11)** | **Positive†**  **(n= 19)** | ***P***  **value** |
| **Sex**  Male, n (%)  Female, n (%) | 6(40) 9(60) | 13(65) 7(35) | 0.142 | 1(25) 3(75) | 1(100) 0(0) | 0.171 | 5(45.5)  6(54.5) | 12(63.2)  7(36.8) | 0.346 |
| **Age (years)** | 52.53±16.44 | 50.05±18.68 | 0.685 | 48.75±17.63 | 59±0 | 0.639 | 53.91±16.65 | 49.58±19.07 | 0.536 |
| **BMI (kg/m²)** | 27.43±6.16 | 26.35±5.62 | 0.592 | 25.83±5.85 | 27.72±0 | 0.792 | 28.01±6.44 | 26.27±5.76 | 0.453 |
| **EBV status**  EBV−, n (%)  EBV+, n (%) | 4(26.67) 11(73.33) | 1(5) 19(95) | 0.070 |  |  |  |  |  |  |
| **WHO histological classification**  Keratinizing SCC (K-NPC), n (%)  Non-keratinizing SCC (NK-NPC), n (%) | 1(6.67) 14(93.33) | 1(5) 19(95) | 0.833 | 1(25) 3(75) | 1(100) 0(0) | 0.171 | 0(0) 11(100) | 0(0) 19(100) | **-** |
| **Primary tumor (T) category**  T1, n (%)  T2, n (%)  T3, n (%)  T4, n (%) | 0(0) 1(6.67) 8(53.33) 6(40) | 3(15) 2(10) 7(35) 8(40) | 0.387 | 0(0) 0(0) 3(75) 1(25) | 0(0) 0(0) 0(0) 1(100) | 0.171 | 0(0) 1(9.09) 5(45.45) 5(45.45) | 3(15.79) 2(10.53) 7(36.84) 7(36.84) | 0.570 |
| **Regional lymph nodes (N) category**  N0, n (%)  N1, n (%)  N2, n (%)  N3, n (%) | 3(20) 3(20) 7(46.67) 2(13.33) | 1(5) 5(25) 12(60) 2(10) | 0.543 | 1(25) 1(25) 1(25) 1(25) | 0(0) 0(0) 1(100) 0(0) | 0.599 | 2(18.18) 2(18.18) 6(54.55) 1(9.09) | 1(5.26) 5(26.32) 11(57.89) 2(10.53) | 0.708 |
| **Distant metastasis (M1) category**  **No distant metastasis (M0) category**  M0, n (%)  M1, n (%) | 14(93.33) 1(6.67) | 16(80) 4(20) | 0.265 | 4(100) 0(0) | 1(100) 0(0) | **-** | 10(90.91) 1(9.09) | 15(78.95) 4(21.05) | 0.397 |
| **AJCC staging**  II, n (%)  III, n (%)  IVA, n (%)  IVB, n (%) | 1(6.67) 6(40) 7(46.67) 1(6.67) | 1(5) 9(45) 6(30) 4(20) | 0.615 | 0(0) 2(50) 2(50) 0(0) | 0(0) 0(0) 1(100) 0(0) | 0.361 | 1(9.09) 4(36.36) 5(45.45) 1(9.09) | 1(5.26)  9(47.37) 5(26.32) 4(21.05) | 0.634 |
| **SEER stage**  Localized, n (%)  Regional, n (%)  Distant, n (%) | 3(20) 11(73.33) 1(6.67) | 1(5) 15(75) 4(20) | 0.252 | 1(25) 3(75) 0(0) | 0(0) 1(100) 0(0) | 0.576 | 2(18.18) 8(72.73)  1(9.09) | 1(5.26) 14(73.68) 4(21.05) | 0.414 |
| **Recurrence**  Yes, n (%)  No, n (%) | 1(6.67) 14(93.33) | 4(20) 16(80) | 0.265 | 0(0) 4(100) | 0(0) 1(100) | **-** | 1(9.09) 10(90.91) | 4(21.05) 15(78.95) | 0.397 |
| **RFS (months)**  Yes, n (%)  No, n (%) | 14(93.33) 1(6.67) | 16(80) 4(20) | 0.265 | 4(100)  0(0) | 1(100) 0(0) | **-** | 10(90.91) 1(9.09) | 15(78.95) 4(21.05) | 0.397 |
| **DMFS (months)**  Yes, n (%)  No, n (%) | 14(93.33) 1(6.67) | 16(80) 4(20) | 0.265 | 4(100) 0(0) | 1(100) 0(0) | **-** | 10(90.91) 1(9.09) | 15(78.95) 4(21.05) | 0.397 |
| **OS (months)**  Alive, n (%)  Dead, n (%) | 13(86.67) 2(13.33) | 17(85) 3(15) | 0.889 | 4(100) 0(0) | 1(100) 0(0) | **-** | 9(81.82) 2(18.18) | 16(84.21) 3(15.79) | 0.865 |

Immunoreactivity (immunostaining) of autophagy-related proteins was scored based on the staining intensity level as follows: 0 (no staining, negative), 1 (light brown staining, weak positive), 2 (medium brown staining, moderate positive), 3 (dark brown staining, strong positive). Data are represented as the number of cases (%). * indicates a statistical significant difference.

**Table S16.** Comparison of demographic and clinical characteristics with ATG4C protein immunoreactivity in NPC patients

| **Characteristic** | **ATG4C protein immunoreactivity** | | | | | | | | |
| --- | --- | --- | --- | --- | --- | --- | --- | --- | --- |
|  | **Entire series of NPC patients**  **(n=35)** | | | **NPC EBV−**  **(n=5)** | | | **NPC EBV+**  **(n=30)** | | |
|  | **Negative†**  **(n= 13)** | **Positive†**  **(n=22)** | ***P* value** | **Negative†**  **(n=5)** | **Positive†**  **(n=0)** | ***P* value** | **Negative†**  **(n=8)** | **Positive†**  **(n=22)** | ***P* value** |
| **Sex**  Male, n (%)  Female, n (%) | 6(46.15) 7(53.85) | 13(59.09) 9(40.91) | 0.458 | 2(40) 3(60) |  |  | 4(50) 4(50) | 13(59.1) 9(40.9) | 0.657 |
| **Age (years)** | 53.85±19.63 | 49.5±16.46 | 0.487 | 50.8±15.94 |  |  | 55.75±22.46 | 49.5±16.46 | 0.411 |
| **BMI (kg/m²)** | 26.44±6.78 | 27.03±5.28 | 0.778 | 26.21±5.14 |  |  | 26.59±7.98 | 27.03±5.28 | 0.863 |
| **EBV status**  EBV−, n (%)  EBV+, n (%) | 5(38.46) 8(61.54) | 0(0) 22(100) | 0.002* |  |  |  |  |  |  |
| **WHO histological classification**  Keratinizing SCC (K-NPC), n (%)  Non-keratinizing SCC (NK-NPC), n (%) | 2(15.38) 11(84.62) | 0(0) 22(100) | 0.058 | 2(40) 3(60) |  |  | 0(0) 8(100) | 0(0) 22(100) | **-** |
| **Primary tumor (T) category**  T1, n (%)  T2, n (%)  T3, n (%)  T4, n (%) | 0(0) 1(7.69) 8(61.54) 4(30.77) | 3(13.64) 2(9.09) 7(31.82) 10(45.45) | 0.271 | (0) (0) 3(60) 2(40) |  |  | 0(0) 1(12.5) 5(62.5) 2(25) | 3(13.64) 2(9.09) 7(31.82) 10(45.45) | 0.369 |
| **Regional lymph nodes (N) category**  N0, n (%)  N1, n (%)  N2, n (%)  N3, n (%) | 2(15.38) 3(23.08) 6(46.15)  2(15.38) | 2(9.09) 5(22.73)  13(59.09) 2(9.09) | 0.845 | 1(20) 1(20) 2(40) 1(20) |  |  | 1(12.5) 2(25) 4(50) 1(12.5) | 2(9.09) 5(22.73) 13(59.09) 2(9.09) | 0.972 |
| **Distant metastasis (M) category**  M0, n (%)  M1, n (%) | 13(100) 0(0) | 17(77.27) 5(22.73) | 0.063 | 5(100) 0(0) |  |  | 8(100) 0(0) | 17(77.27) 5(22.73) | 0.140 |
| **AJCC staging**  II, n (%)  III, n (%)  IVA, n (%)  IVB, n (%) | 1(7.69) 6(46.15) 6(46.15) 0(0) | 1(4.55) 9(40.91) 7(31.82) 5(22.73) | 0.308 | 0(0) 2(40) 3(60) 0(0) |  |  | 1(12.5) 4(50) 3(37.5) 0(0) | 1(4.55) 9(40.91) 7(31.82) 5(22.73) | 0.467 |
| **SEER stage**  Localized, n (%)  Regional, n (%)  Distant, n (%) | 2(15.38) 11(84.62)  0(0) | 2(9.09) 15(68.18) 5(22.73) | 0.171 | 1(20) 4(80) 0(0) |  |  | 1(12.5) 7(87.5)  0(0) | 2(9.09) 15(68.18) 5(22.73) | 0.335 |
| **Recurrence**  Yes, n (%)  No, n (%) | 1(7.69) 12(92.31) | 4(18.18)  18(81.82) | 0.392 | 0(0) 5(100) |  |  | 1(12.5) 7(87.5) | 4(18.18) 18(81.82) | 0.712 |
| **RFS (months)**  Yes, n (%)  No, n (%) | 12(92.31) 1(7.69) | 18(81.82)  4(18.18) | 0.392 | 5(100) 0(0) |  |  | 7(87.5) 1(12.5) | 18(81.82) 4(18.18) | 0.712 |
| **DMFS (months)**  Yes, n (%)  No, n (%) | 13(100) 0(0) | 17(77.27) 5(22.73) | 0.063 | 5(100) 0(0) |  |  | 8(100) 0(0) | 17(77.27) 5(22.73) | 0.140 |
| **OS (months)**  Alive, n (%)  Dead, n (%) | 12(92.31) 1(7.69) | 18(81.82) 4(18.18) | 0.392 | 5(100) 0(0) |  |  | 7(87.5) 1(12.5) | 18(81.82) 4(18.18) | 0.712 |

Immunoreactivity (immunostaining) of autophagy-related proteins was scored based on the staining intensity level as follows: 0 (no staining, negative), 1 (light brown staining, weak positive), 2 (medium brown staining, moderate positive), 3 (dark brown staining, strong positive). Data are represented as the number of cases (%). * indicates a statistical significant difference.

**Table S17.** Comparison of demographic and clinical characteristics with ATG4D protein immunoreactivity in NPC patients

| **Characteristic** | **ATG4D protein immunoreactivity** | | | | | | | | |
| --- | --- | --- | --- | --- | --- | --- | --- | --- | --- |
|  | **Entire series of NPC patients**  **(n=35)** | | | **NPC EBV−**  **(n=5)** | | | **NPC EBV+**  **(n=30)** | | |
|  | **Negative†**  **(n=33)** | **Positive†**  **(n=2)** | ***P***  **value** | **Negative†**  **(n= 5)** | **Positive†**  **(n=0)** | ***P* value** | **Negative†**  **(n=28)** | **Positive†**  **(n=2)** | ***P***  **value** |
| **Sex**  Male, n (%)  Female, n (%) | 18(54.55) 15(45.45) | 1(50) 1(50) | 0.900 | 2(40)  3(60) |  |  | 16(57.1) 12(42.9) | 1(50) 1(50) | 0.844 |
| **Age (years)** | 51.15±17.99 | 50.5±10.61 | 0.960 | 50.8±15.94 |  |  | 51.21±18.6 | 50.5±10.61 | 0.958 |
| **BMI (kg/m²)** | 26.53±5.6 | 31.33±9.74 | 0.262 | 26.21±5.14 |  |  | 26.59±5.76 | 31.33±9.74 | 0.286 |
| **EBV status**  EBV−, n (%)  EBV+, n (%) | 5(15.15) 28(84.85) | 0(0) 2(100) | 0.552 |  |  |  |  |  |  |
| **WHO histological classification**  Keratinizing SCC (K-NPC), n (%)  Non-keratinizing SCC (NK-NPC), n (%) | 2(6.06) 31(93.94) | 0(0) 2(100) | 0.720 | 2(40) 3(60) |  |  | 0(0) 28(100) | 0(0)  2(100) | **-** |
| **Primary tumor (T) category**  T1, n (%)  T2, n (%)  T3, n (%)  T4, n (%) | 2(6.06) 2(6.06) 15(45.45) 14(42.42) | 1(50) 1(50) 0(0) 0(0) | 0.017* | (0) (0) 3(60) 2(40) |  |  | 2(7.14) 2(7.14) 12(42.86) 12(42.86) | 1(50) 1(50) 0(0) 0(0) | 0.036* |
| **Regional lymph nodes (N) category**  N0, n (%)  N1, n (%)  N2, n (%)  N3, n (%) | 4(12.12) 8(24.24) 17(51.52) 4(12.12) | 0(0) 0(0) 2(100) 0(0) | 0.618 | 1(20) 1(20) 2(40) 1(20) |  |  | 3(10.71) 7(25) 15(53.57) 3(10.71) | 0(0) 0(0) 2(100) 0(0) | 0.651 |
| **Distant metastasis (M1) category**  **No distant metastasis (M0) category**  M0, n (%)  M1, n (%) | 28(84.85) 5(15.15) | 2(100) 0(0) | 0.552 | 5(100) 0(0) |  |  | 23(82.14) 5(17.86) | 2(100) 0(0) | 0.513 |
| **AJCC staging**  II, n (%)  III, n (%)  IVA, n (%)  IVB, n (%) | 2(6.06) 13(39.39) 13(39.39) 5(15.15) | 0(0) 2(100) 0(0) 0(0) | 0.419 | 0(0) 2(40) 3(60) 0(0) |  |  | 2(7.14) 11(39.29) 10(35.71) 5(17.86) | 0(0) 2(100) 0(0) 0(0) | 0.423 |
| **SEER stage**  Localized, n (%)  Regional, n (%)  Distant, n (%) | 4(12.12) 24(72.73) 5(15.15) | 0(0) 2(100) 0(0) | 0.693 | 1(20) 4(80) 0(0) |  |  | 3(10.71) 20(71.43) 5(17.86) | 0(0) 2(100) 0(0) | 0.677 |
| **Recurrence**  Yes, n (%)  No, n (%) | 5(15.15) 28(84.85) | 0(0) 2(100) | 0.552 | 0(0) 5(100) |  |  | 5(17.86) 23(82.14) | 0(0) 2(100) | 0.513 |
| **RFS (months)**  Yes, n (%)  No, n (%) | 28(84.85) 5(15.15) | 2(100) 0(0) | 0.552 | 5(100) 0(0) |  |  | 23(82.14) 5(17.86) | 2(100) 0(0) | 0.513 |
| **DMFS (months)**  Yes, n (%)  No, n (%) | 28(84.85) 5(15.15) | 2(100) 0(0) | 0.552 | 5(100) 0(0) |  |  | 23(82.14) 5(17.86) | 2(100) 0(0) | 0.513 |
| **OS (months)**  Alive, n (%)  Dead, n (%) | 29(87.88) 4(12.12) | 1(50) 1(50) | 0.137 | 5(100) 0(0) |  |  | 24(85.71) 4(14.29) | 1(50) 1(50) | 0.190 |

Immunoreactivity (immunostaining) of autophagy-related proteins was scored based on the staining intensity level as follows: 0 (no staining, negative), 1 (light brown staining, weak positive), 2 (medium brown staining, moderate positive), 3 (dark brown staining, strong positive). Data are represented as the number of cases (%). * indicates a statistical significant difference.

**Table S18.** Comparison of demographic and clinical characteristics with ATG5 protein immunoreactivity in NPC patients

| **Characteristic** | **ATG5 protein immunoreactivity** | | | | | | | | |
| --- | --- | --- | --- | --- | --- | --- | --- | --- | --- |
|  | **Entire series of NPC patients**  **(n=35)** | | | **NPC EBV−**  **(n=5)** | | | **NPC EBV+**  **(n=30)** | | |
|  | **Negative†**  **(n=0 )** | **Positive†**  **(n=35)** | ***P* value** | **Negative†**  **(n=0)** | **Positive†**  **(n=5)** | ***P* value** | **Negative†**  **(n=0)** | **Positive†**  **(n=30)** | ***P* value** |
| **Sex**  Male, n (%)  Female, n (%) |  | 19(54.29) 16(45.71) |  |  | 2(40) 3(60) |  |  | 17(56.7) 13(43.3) |  |
| **Age (years)** |  | 51.11±17.55 |  |  | 50.8±15.94 |  |  | 51.17±18.05 |  |
| **BMI (kg/m²)** |  | 26.81±5.79 |  |  | 26.21±5.14 |  |  | 26.91±5.97 |  |
| **EBV status**  EBV−, n (%)  EBV+, n (%) |  | 5(14.29) 30(85.71) |  |  |  |  |  |  |  |
| **WHO histological classification**  Keratinizing SCC (K-NPC), n (%)  Non-keratinizing SCC (NK-NPC), n (%) |  | 2(5.71) 33(94.29) |  |  | 2(40) 3(60) |  |  | 0(0) 30(100) |  |
| **Primary tumor (T) category**  T1, n (%)  T2, n (%)  T3, n (%)  T4, n (%) |  | 3(8.57) 3(8.57) 15(42.86) 14(40) |  |  | 0(0) 0(0) 3(60) 2(40) |  |  | 3(10) 3(10) 12(40) 12(40) |  |
| **Regional lymph nodes (N) category**  N0, n (%)  N1, n (%)  N2, n (%)  N3, n (%) |  | 4(11.43) 8(22.86) 19(54.29) 4(11.43) |  |  | 1(20) 1(20) 2(40) 1(20) |  |  | 3(10) 7(23.33) 17(56.67) 3(10) |  |
| **Distant metastasis (M1) category**  **No distant metastasis (M0) category**  M0, n (%)  M1, n (%) |  | 30(85.71) 5(14.29) |  |  | 5(100) 0(0) |  |  | 25(83.33) 5(16.67) |  |
| **AJCC staging**  II, n (%)  III, n (%)  IVA, n (%)  IVB, n (%) |  | 2(5.71) 15(42.86) 13(37.14) 5(14.29) |  |  | 0(0) 2(40) 3(60) 0(0) |  |  | 2(6.67) 13(43.33) 10(33.33) 5(16.67) |  |
| **SEER stage**  Localized, n (%)  Regional, n (%)  Distant, n (%) |  | 4(11.43) 26(74.29) 5(14.29) |  |  | 1(20) 4(80)  0(0) |  |  | 3(10) 22(73.33) 5(16.67) |  |
| **Recurrence**  Yes, n (%)  No, n (%) |  | 5(14.29) 30(85.71) |  |  | 0(0) 5(100) |  |  | 5(16.67) 25(83.33) |  |
| **RFS (months)**  Yes, n (%)  No, n (%) |  | 30(85.71) 5(14.29) |  |  | 5(100) 0(0) |  |  | 25(83.33) 5(16.67) |  |
| **DMFS (months)**  Yes, n (%)  No, n (%) |  | 30(85.71) 5(14.29) |  |  | 5(100) 0(0) |  |  | 25(83.33) 5(16.67) |  |
| **OS (months)**  Alive, n (%)  Dead, n (%) |  | 30(85.71) 5(14.29) |  |  | 5(100) 0(0) |  |  | 25(83.33) 5(16.67) |  |

Immunoreactivity (immunostaining) of autophagy-related proteins was scored based on the staining intensity level as follows: 0 (no staining, negative), 1 (light brown staining, weak positive), 2 (medium brown staining, moderate positive), 3 (dark brown staining, strong positive). Data are represented as the number of cases (%). * indicates a statistical significant difference.
